# Non-Linear Quantitative Structure–Activity Relationships Modelling, Mechanistic Study and In-Silico Design of Flavonoids as Potent Antioxidants

**DOI:** 10.3390/ijms20092328

**Published:** 2019-05-10

**Authors:** Petar Žuvela, Jonathan David, Xin Yang, Dejian Huang, Ming Wah Wong

**Affiliations:** 1 Department of Chemistry, National University of Singapore, 3 Science Drive 3, Singapore 117543, Singapore; petar.zuvela@nus.edu.sg (P.Ž.); jonathan.david14@sps.nus.edu.sg (J.D.); 2 Food Science and Technology Program, Department of Chemistry, National University of Singapore, 3 Science Drive 3, Singapore 117543, Singapore; xinyang@u.nus.edu (X.Y.); chmhdj@nus.edu.sg (D.H.)

**Keywords:** antioxidant activity, ORAC, flavonoids, QSAR, ANNs, mechanism, PaD method

## Abstract

In this work, we developed quantitative structure–activity relationships (QSAR) models for prediction of oxygen radical absorbance capacity (ORAC) of flavonoids. Both linear (partial least squares—PLS) and non-linear models (artificial neural networks—ANNs) were built using parameters of two well-established antioxidant activity mechanisms, namely, the hydrogen atom transfer (HAT) mechanism defined with the minimum bond dissociation enthalpy, and the sequential proton-loss electron transfer (SPLET) mechanism defined with proton affinity and electron transfer enthalpy. Due to pronounced solvent effects within the ORAC assay, the hydration energy was also considered. The four-parameter PLS-QSAR model yielded relatively high root mean square errors (RMSECV = 0.783, RMSEE = 0.668, RMSEP = 0.900). Conversely, the ANN-QSAR model yielded considerably lower errors (RMSEE = 0.180 ± 0.059, RMSEP1 = 0.164 ± 0.128, and RMSEP2 = 0.151 ± 0.114) due to the inherent non-linear relationships between molecular structures of flavonoids and ORAC values. Five-fold cross-validation was found to be unsuitable for the internal validation of the ANN-QSAR model with a high RMSECV of 0.999 ± 0.253; which is due to limited sample size where resampling with replacement is a considerably better alternative. Chemical domains of applicability were defined for both models confirming their reliability and robustness. Based on the PLS coefficients and partial derivatives, both models were interpreted in terms of the HAT and SPLET mechanisms. Theoretical computations based on density functional theory at ωb97XD/6-311++G(d,p) level of theory were also carried out to further shed light on the plausible mechanism of anti-peroxy radical activity. Calculated energetics for simplified models (genistein and quercetin) with peroxyl radical derived from 2,2′-azobis (2-amidino-propane) dihydrochloride suggested that both SPLET and single electron transfer followed by proton loss (SETPL) mechanisms are competitive and more favorable than HAT in aqueous medium. The finding is in good accord with the ANN-based QSAR modelling results. Finally, the strongly predictive ANN-QSAR model was used to predict antioxidant activities for a series of 115 flavonoids designed combinatorially with flavone as a template. Structural trends were analyzed, and general guidelines for synthesis of new flavonoid derivatives with potentially potent antioxidant activities were given.

## 1. Introduction

Flavonoids belong to a class of naturally-occurring polyphenols ubiquitous to plant life, especially in vegetables, fruits, flowers, seeds, and grains [1]. Generally, flavonoids can act as antioxidants with a variety of known mechanisms: (i) free radical scavenging, (ii) proton donation, (iii) singlet oxygen quenching, and (iv) transition metal ion chelation [2]. Reactive oxygen species are regularly generated in biological systems as by-products of several metabolism processes, such as energy production, cell growth regulation, and intercellular signaling [3]. Reactive oxygen species scavenging activity and antioxidants imbalance can, however, lead to oxidative damage of cell membranes, important proteins and enzymes, and DNA strands. Consequently, reactive oxygen species are believed to be the causative factor of a multitude of chronic illnesses, such as cancer, heart disease, and accelerated aging [4]. In biological systems, there are several antioxidant physiological defenses, and flavonoids in particular with large antioxidant capacities contribute to them greatly (e.g., via anti-inflammatory action) [5].

Quantitative structure–activity relationships (QSAR) models are extensively used for antioxidant activity prediction, understanding the governing mechanisms, and design of more potent compounds [6]. When it comes to flavonoids, several QSAR models were developed throughout the years for prediction of their antioxidant activity. For instance, Lien et al. have developed a QSAR model correlating Trolox-equivalent antioxidant capacity (TEAC) with the number and position of hydroxyl groups within the flavonoid ring system [7]. Building upon their work, Amić et al. developed a QSAR model for free radical scavenging activity prediction using the position of the hydroxyl groups as a molecular descriptor [8]. These models have exhibited reasonable predictive ability and were statistically significant. Moreover, in the study of Amić et al., an activity cliff has formed clustering the compounds into two distinct groups on the plot of predictive ability of the model, which points to potential overfitting [8].

Besides the number and positions of hydroxyl groups, Rasulev et al. and Ray et al. have used theoretical (e.g., quantum chemical), and empirical molecular descriptors for QSAR modelling [9,10]. The resulting QSAR models were of superior predictive ability. However, no external validation was performed, and the chemical domain of applicability was not defined. To the authors’ knowledge, QSAR studies on prediction of flavonoids antioxidant activity that would completely satisfy all the OECD QSAR principles are virtually non-existent [11].

Due to the inherent complexity of the relationships between molecular structure of antioxidants and their activity, linear models are often insufficient for its prediction [12]. Therefore, in this work, besides a linear model (based on partial least squares (PLS) with the statistically inspired modification of PLS (SIMPLS) algorithm) typically employed in such studies, we have employed an approach using artificial neural networks (ANNs) for prediction of oxygen radical absorbance capacity (ORAC expressed in mM TE) of flavonoids [13,14]. Distinguishable from other studies, in our work we have not only extensively validated the QSAR models, but also interpreted both the developed models. ANN-based QSAR models were interpreted through the use of the partial derivative (PaD) method as opposed to the typical black box approach. Finally, the chemical domain of applicability was defined [12,15].

Ease of interpretability of the developed QSAR models is due to the fact that they were built using quantum mechanical (QM) descriptors calculated using density functional theory (DFT) [16,17]. Typically, parameters of the hydrogen atom transfer (HAT) mechanism such as the number of hydroxyl groups (*n*(OH)), and minimum bond dissociation enthalpy (BDE_min_) are used, which were thought to be predominant in ORAC assays [18]. As such, the antioxidant activity is thought to be accounted for with the minimum BDE which estimates the least energy required to homolytically cleave the O-H bond at 298 K; where higher BDE corresponds to a slower HAT process. However, studies (including our own) have shown that there is a poor correlation between ORAC values and BDE_min_, with *r* = −0.202, *p* > 0.05 [19]. As opposed to the TEAC assay, there could exist more prevalent mechanisms (see Figure S1) other than the conventional HAT mechanism in the ORAC assay. The sequential proton-loss electron transfer (SPLET) and single electron transfer followed by proton loss (SETPL) mechanisms are often found to be competing. The SPLET mechanism defined with electron transfer enthalpy (ETE) and proton affinity (PA) [20] can be preferred to HAT, especially when the assay is performed in a hydrophilic medium instead of a lipophilic one [19,21]. Since only the SPLET mechanism has been thoroughly studied in the literature, we have focused only on HAT and SPLET mechanisms in current QSAR modelling [19,20,21]. As a result, in the scope of this study, we have used parameters of both the HAT and SPLET mechanisms in implicit water solvent for building the PLS- and ANN-QSAR models. Based on the developed ANN-QSAR model, ORAC values were predicted for a set of flavonoids designed with an in-silico combinatorial approach. Consequently, general guidelines and perspectives for synthesis of better and potentially more potent antioxidants were given. To further support our hypothesis, we have examined the energetics of all possible mechanisms (HAT, SPLET, and SETPL) of the interaction of two flavonoids (namely genistein and quercetin) with the radical initiator 2,2′-azobis (2-amidino-propane) dihydrochloride (AAPH) in both the gas phase and implicit aqueous solvent.

## 2. Results and Discussion

### 2.1. Linear PLS-QSAR Model

**Figure 1:**
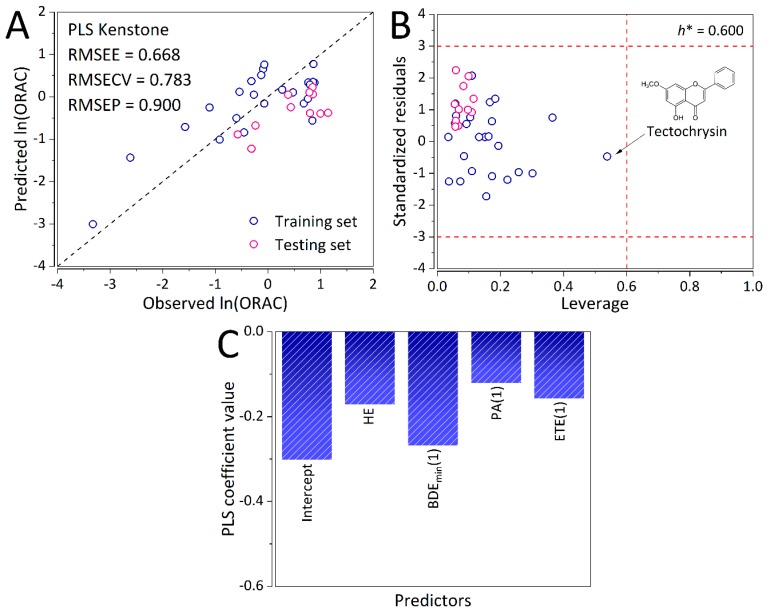
(**A**) Predictive ability of the partial least squares (PLS)-based quantitative structure–activity relationships (QSAR) model. Navy blue circles denote the training, while pink circles denote the testing set observations (*n* = 36). (**B**) Applicability domain of the PLS-based QSAR model. Navy blue circles denote the training, while pink circles denote the testing set observations. Critical leverage (h*) value is 0.600 (*n* = 36). (**C**) Distribution of the coefficients of the PLS-based QSAR model.

The PLS-based QSAR model (Equation (1)) has been extensively validated through both LOOCV and external validation. The optimal number of latent variables (LVs) was also determined through LOOCV. As it can be observed from Figure S2, the optimal number of LVs was found to be 2 with the minimum RMSECV of 0.783. Predictive ability of the cross-validated PLS-QSAR model is visualized in Figure S3, while its predictive ability on the external validation set is depicted in Figure 1A. Even though all the points are well dispersed along the ideal *y* = *x* line, the corresponding errors are still quite high. More precisely, the model has exhibited a root mean square error of cross-validation (RMSECV) of 0.783, root mean square error of estimation (RMSEE) of 0.668, and root mean square error of prediction (RMSEP) of 0.900. Furthermore, the PLS-based QSAR model was found to be statistically significant with an *F* value of 18.53, and a *p* value of 5.70 × 10^−3^. The PLS-based QSAR model has also shown to be reliable and robust as all the compounds were found to lay within the chemical domain of applicability of the model (Figure 1B).

(1) $$\begin{array}{ccc} \ln\left(ORAC\right) & = & -0.049\left(\pm 0.022\right)-0.219\left(\pm 0.019\right)HE-\\ & & \;0.264\left(\pm 0.017\right)BDE_{\min}(1)-0.193\left(\pm 0.017\right)PA(1)-\\ & & \;0.090\left(\pm 0.010\right)ETE(1)\\ F & = & 18.53;p=5.7\times 10^{-3} \end{array}$$

### 2.2. Interpretation of the PLS-QSAR Model

All the coefficients of the developed PLS-based QSAR model have exhibited negative values. This is consistent with the assumed SPLET and HAT mechanisms (Figure 1C). Specifically, the lower the proton affinity (PA (1)) and electron transfer enthalpy (ETE (1)) barriers are, the higher the ORAC. Accordingly, the lower the minimum bond dissociation enthalpy (BDE_min_ (1)) is, ORAC of a compounds will be higher. Finally, since all the species involved in the SPLET mechanism are well solvated, the lower hydration energy (HE) increases the antioxidant capacity. Interestingly, the linear model has shown that the BDE_min_ (1) parameter is the most important, followed by the HE, PA (1), and ETE (1) parameters. This inconsistency stems from the inherent complex non-linear relationships between QM parameters of molecular structure of flavonoids and their corresponding ORAC values.

### 2.3. Non-Linear ANN-QSAR Model

First, the ANN architecture was optimized using the protocol of Žuvela et al. consisted of eight hidden neurons, using the Levenberg-Marquardt algorithm with mean squared error (MSE) as a pointwise loss function for training [12,22,23]. As opposed to the PLS-QSAR model, for ANN-QSAR models the dataset was randomly separated into 26 training, 5 testing, and 5 validation entries. Randomized training was repeated 100 times in order to obtain a standard deviation for both the predictions and the corresponding errors.

### 2.4. Validation of the ANN-QSAR Model

**Figure 2:**
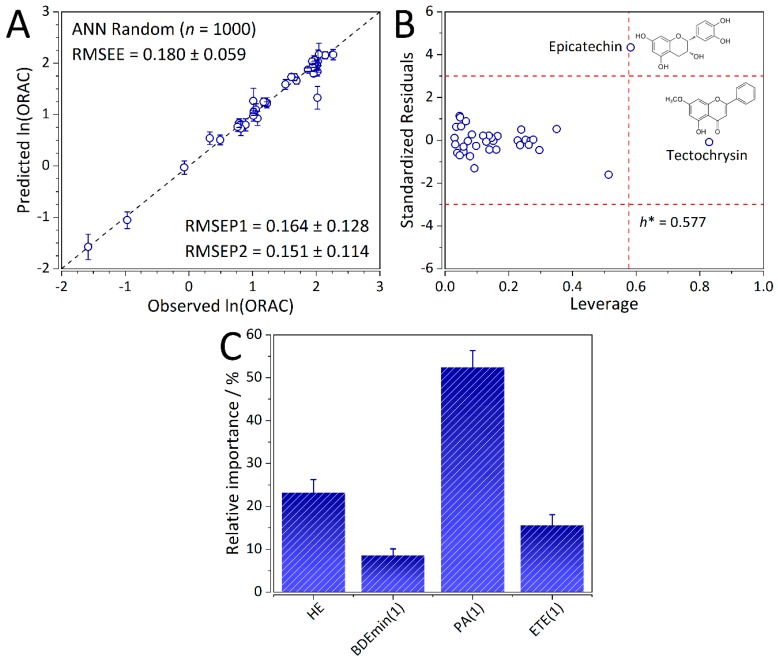
(**A**) Predictive ability of the artificial neural network (ANN)-based QSAR model. (**B**) Applicability domain of the ANN-based QSAR model (*n* = 36). (**C**) Relative contributions of the four molecular descriptors towards the targets (ORAC) as calculated using the PaD method. Legend; hydration energy (HE), minimum bond dissociation enthalpy (BDE_min_ (1)), electron transfer enthalpy (ETE (1)), and proton affinity (PA (1)). For the first three descriptors, the index 1 represents the first oxidation step. Error bars represent the standard deviation of predictions based on 1000 ANN training cycles.

The ANN-QSAR model has been trained in 1000 cycles (resampling with replacement) in which the training, testing, and validation sets were randomized. In fact, the number of hidden neurons has been determined based on errors calculated for each set (i.e., mean error ± standard deviation). Five-fold cross-validation (CV) has also been performed. However, with such a limited number of samples it is unsuitable for internal validation (with an RMSECV of 0.999 ± 0.253; Figure S4) and resampling with replacement is a more viable option. Moreover, splitting the dataset into five folds for CV has considerably decreased the amount of samples for training the ANN model resulting in considerably higher error. Thereby, the developed ANN-based QSAR model was found to be strongly predictive with 0.180 ± 0.059, 0.164 ± 0.128, and 0.151 ± 0.114 for RMSEE, RMSEP on the testing, and RMSEP on the validation sets, respectively (number of training cycles; *n* = 1000). Strong predictive ability of the ANN-QSAR model can be observed from the plot of experimental ln (ORAC) values in dependence with the predicted values (Figure 2A).

Chemical domain of applicability was also defined for the ANN-based QSAR model with average values of standardized residuals and leverages (*n* = 1000). It was found that most of the compounds lie within the warning limits: three multiples of standard deviations of standardized residuals and the critical leverage value of 0.577 (Figure 2B). Two outlying compounds (epicatechin and tectochrysin) can be observed. One of them (tectochrysin) exhibited average leverage values larger than the threshold making them structurally important for the ANN model. They are predicted quite well, confirming stability and robustness of the model. It is worth noting that and tectochrysin exhibited the smallest experimental ORAC values out of all the compounds (0.206 ± 0.017). On the other hand, epicatechin was predicted quite poorly and is an outlier from the dataset.

### 2.5. Interpretation of the ANN-QSAR Model

Typically, in QSAR studies involving ANNs, correlations are either omitted altogether or inferred from a linear model. It is a very misleading and detrimental practice to do so [12]. In this work, we employed the PaD method to independently analyze and interpret the correlations and the underlying causal relationships between the input QM parameters and the output ORAC of the ANN-based QSAR model. Analysis of the normalized sum of squared derivatives (SSD) (Figure 2C) has shown that the proton affinity of the first oxidation step (PA (1)) has shown to be the governing factor towards ORAC, followed by hydration energy (HE), electron transfer enthalpy of the first oxidation step (ETE (1)), and bond dissociation enthalpy of the first oxidation step (BDE_min_ (1)). This is consistent with the assumed SPLET mechanism, where the initial barriers are the *pK*_a_ values of the compounds themselves which in turn were shown to be strongly correlated to PA (1) (*r* = 0.80, *p* < 0.01) [19]. Based on the PaD analysis of the developed ANN-QSAR model, the HE is the second most important parameter contributing to ORAC. As in the case of the PLS-QSAR model, the importance of solvent effects on ORAC are elucidated. The second barrier of the SPLET mechanism, the ETE (1) was ranked the third most important by magnitude of its normalized SSD. Finally, the contribution of BDE_min_ (1) towards ORAC was the lowest.

**Figure 3:**
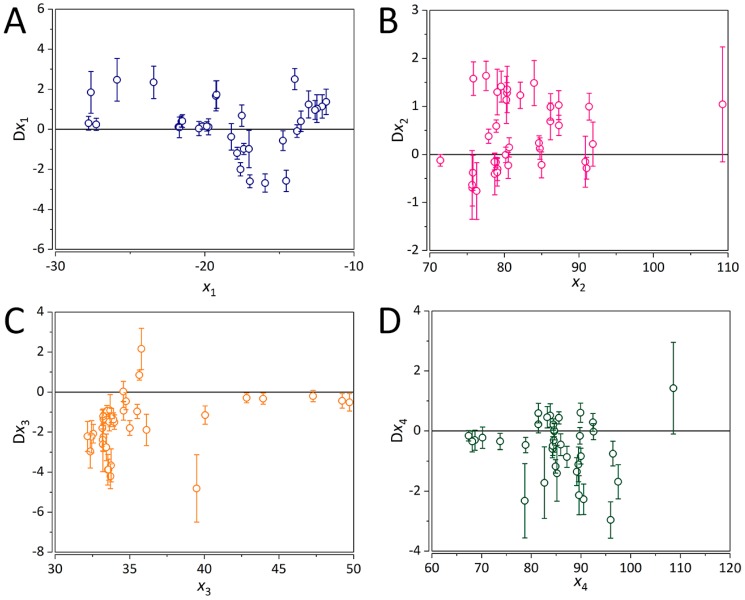
Partial derivatives for each input calculated using the PaD method. For (**A**) hydration energy (HE), (**B**) minimum bond dissociation enthalpy (BDE_min_ (1)), (**C**) proton affinity (PA (1)), and (**D**) electron transfer enthalpy (ETE (1)). For the first three descriptors, the index 1 represents the first oxidation step. Error bars represent the standard deviation of predictions based on 1000 ANN training cycles.

Besides their relative importance towards ORAC, scatter plots of partial derivatives of the four QM parameters against themselves were constructed (Figure 3). It can be observed that for HE (Figure 3A) the majority of partial derivatives are either negative, zero, or close to zero. For BDE_min_ (1), many of the partial derivatives being <0 (Figure 3B). Although there are many positive values, the trend is generally negative. As for the PA (1) and ETE (1), for both most of the partial derivatives are either negative or zero (Figure 3C,D), with a general negative trend. For higher ETE (1) values (~110 kcal/mol), their further increase has a detrimental effect on ORAC. From the analysis of the trends in partial derivatives of the QM descriptors, a strong non-linearity is revealed. Accounting for the complex non-linear trend between the molecular structure of flavonoids and ORAC resulted in a considerable decrease of model error as compared to the PLS-based QSAR model.

### 2.6. Mechanism of Hydrogen Abstraction Transfer by Peroxyl Radical

Various plausible mechanisms have been proposed for understanding the interaction of flavonoid antioxidants with the free radical derived from the assay of oxygen radical absorbance capacity (ORAC).17 However, to date, the actual mechanism has not been determined conclusively. In the ORAC assay, upon thermal decomposition, the reactive oxygen species (ROS) generator, 2,2′-azobis (2-amidino-propane) dihydrochloride (AAPH) produces a peroxyl free radical.17 The redox reactivity of flavonoids (ROH) can follow three possible chemical pathways: (1) HAT, (2) SPLET, and (3) SETPL mechanisms (see Table 1). Understanding the anti-peroxyl radical activity of flavonoids is essential for design of more potent antioxidants.

To gain some insight into the mechanistic aspect of antioxidant activity of flavonoids with the ORAC essay, we examined the HAT, SPLET, and SETPT reaction pathways between two model flavonoids, genistein, and quercetin (ROH), and the peroxyl radical derived from AAPH (PO^•^) by locating the key intermediates on the potential energy surface. The structures and reaction enthalpies (*ΔH_298_*) of various steps of the three reaction pathways were examined at the *ω*B97XD/6-311+G** level in the gas phase (*ε* = 1) and aqueous solution (*ε* = 78.4), using the implicit SMD solvation model. Unless otherwise noted, the relative enthalpies (*ΔH_298_*) given in the text correspond to the aqueous values. The overall hydrogen abstraction reaction is predicted to be thermal neutral (genistein) or slightly exothermic (quercetin) (Table 1). For the HAT mechanism, direct O−H homolytic dissociation enthalpy has a high value of 79−85 kcal/mol. The second SPLET mechanism involves a proton transfer from genistein/quercetin (ROH) to peroxy radical (PO^•^) as the first step, followed by an electron transfer from RO^−^ to POH^•+^. The first step is the rate-determining step with a reaction enthalpy of 32–33 kcal/mol. As one might have expected from the strong solvation effect of the ion pair intermediate (RO^−^ + POH^•+^), the solvent effect in aqueous medium is enormous, with a sizable solvent stabilization energy of ~100 kcal/mol in the first step (Table 1). The third SETPL mechanism, reverse sequence of SPLET, involves an electron transfer in the first step to form ROH^•+^ and PO^−^ as key intermediates, followed by a proton transfer. The first electron transfer step is the rate-limiting step with a calculated enthalpy (33 and 28 kcal/mol, for genistein and quercetin, respectively) comparable to that of the SPLET pathway. Based on the calculated thermodynamics (Table 1), both SPLET and SETPL mechanisms are predicted to be competitive and significantly more favorable than the HAT mechanism. Thus, we conclude that the dominant mechanism for the hydrogen abstraction reaction is likely SPLET or SETPL in the aqueous medium of the ORAC essay. This theoretical finding is in good accord with the ANN-QSAR modelling results, which indicate the important roles of electron transfer enthalpy and hydration energy molecular parameters of the SPLET mechanism. Our thermodynamic result also suggests that SETPL QM descriptors, namely ionization potential (IP) and proton dissociation enthalpy (PDE), could serve as primary descriptors in the development of the QSAR of flavonoid. Finally, we note that the HAT mechanism cannot be ruled out in a nonpolar medium as solvent stabilization of the ion pair intermediate (in SPLET and SETPL) is expected to be small in such an environment.

### 2.7. In-Silico Design of Potent Flavonoid-Based Antioxidants

**Figure 4:**
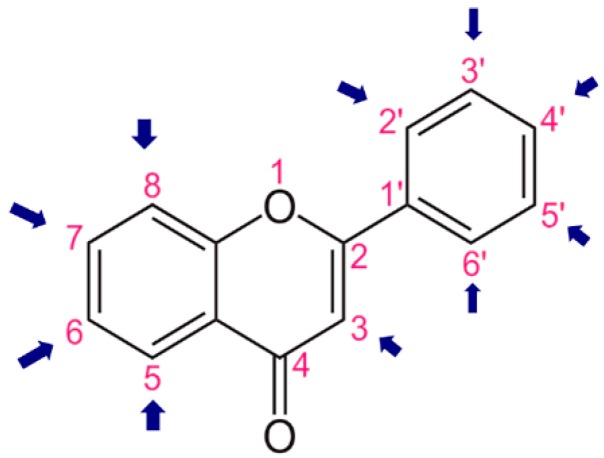
Flavone template structure used for the combinatorial antioxidant design.

Using flavone as a template (Figure 4), aromatic hydrogens bound to aromatic carbons of A-, B- and C-rings were exchanged with hydroxyl, at up to three of its ten possible sites. This yielded 115 possible structures. Based on the values of the four previously mentioned QM parameters (PA (1), ETE (1), BDE_min_ (1), and HE), their leverage values were computed and analyzed. As it can be observed from Figure S5, all the 115 designed compounds fall well within the chemical domain of applicability of the developed models. Using the developed ANN-based QSAR model, the ORAC values of the 115 designed compounds were predicted and can be found in Table S1.

Analyzing the data presented in Table S1, it can be observed that the top 55 combinatorially designed compounds exhibited predicted ORAC values larger than 5, while the bottom 18 have exhibited ORAC values lower than 2. On average for the first top 55 compounds (Table 2), the R3, R6, R7, R8, R2′, R3′, and R5′ groups had the highest frequency. For the bottom 18 compounds (Table 2), R4, R6, R7, and R3′ are the most prominent positions. The overlap between the top 55 and bottom 18 seem to be the R6, R7, and R3′ positions. This points to a fact that OH groups on these positions in combination with OH groups on one or more of the other identified positions (e.g., R4) result in a considerable decrease in ORAC value. Indeed, as it can be observed from Table S1, for instance, compound #115 with a predicted ORAC value of 0.291 has OH groups at only at the R6 group, while e.g., compound #105 has an OH group on the R4 position, besides the R6 and R2′ positions. Thereby, four important positions to place OH groups were identified. R6, R2′, and R3′ positions, with 20, and R7 with 19 occurrences within the first 55. The identified positions are crucial in the design of novel flavonoids as potentially more potent antioxidants.

## 3. Materials and Methods

### 3.1. Chemicals and Instruments

Fluorescein disodium and 6-hydroxy-2,5,7,8-tetramethyl-2-carboxylic acid (Trolox) were purchased from Aldrich (Milwaukee, WI, USA), while 2,2‘-Azobis (2-amidino-propane) dihydrochloride (AAPH) was obtained from Manchester Organics Ltd (Runcorn, UK). Thirty six flavonoids (namely, genistein, naringenin, scutellarin, 3,5,7,8,3′,4′-hexahydroxyflavone, epicatechin, kaempferol, eriodictyol, apigenin, quercetin, liquiritigenin, fisetin, taxifolin, hesperetin, 3,3′,4′-trihydroxyflavone, 7,3′,4′-trihydroxyflavone, diosmetin, luteolin, morin, epigallocatechin, 5,3′,4′-trihydroxyflavone, ampelopsin, myricetin, wogonin, 7,8-dihydroxyflavone, ampelopsin, myricetin, wogonin, 7,8-dihydroxyflavone, chrysin, pinocembrin, catechin, eupatilin, baicaLien, pectolinaringenin, 3,5-dyhydroxyflavone, alpinetin, galangin, genkwanin, primuletin, tectochrysin) were purchased from Nanjing Plant Origin Biological Technology Co., Ltd. (Nanjing, China). All other standards were purchased from Sigma Aldrich (St. Louis, MO, USA). The ORAC assay was performed using a FL600 microplate fluorescence reader (Bio-Tek Instruments, Inc., Winooski, VT, USA). The fluorescence filters with an excitation wavelength of 485 ± 20 nm and an emission wavelength of 530 ± 25 nm were used. KC4 3.0 (rev 29) software was used to control the plate reader, while the samples were diluted using the Precision XS automating pipetting system controlled using the Precision power software 1.0 (Bio-Tek Instruments, Inc.). The 96-well polystyrene microplates and the corresponding covers were obtained from VWR International Inc (Bridgeport, NJ, USA).

### 3.2. Preparation of Standards and Solutions

0.414g of 2,2′-Azobis(2-amidinopropane) dihydrochloride (AAPH) was entirely dissolved in a volume of 10 mL of phosphate buffer (75 mM, pH 7.4). A final concentration of 153 mM was achieved and the AAPH was kept in an ice bath. Unused AAPH solutions are discarded within a period of 8 h. Stock solutions of fluorescein in a concentration of 4.19 × 10^−3^ mM were buffered with a 75 mM phosphate buffer to achieve a pH of 7.4 and were kept at 4 °C in the dark. Fresh fluorescein was further diluted in 75 mM phosphate buffer (pH value of 7.4) to prepare working fluorescein concentrations of 8.16 × 10^−5^ mM. For the Trolox standard; 0.250 g of Trolox was dissolved in 50 mL of 75 mM phosphate buffer (pH 7.4) for a final concentration of 0.02 M. Using the same phosphate buffer, the Trolox stock solutions in concentrations of 50, 25, 12.5, and 6.25 μM were prepared. All the analyzed flavonoids were dissolved in an acetone/water (50:50, *v*/*v*) mixture for an initial stock concentration of 1.0 mM and was stored at −30 °C in the dark. Out of the stock solution, working solutions were prepared by dilution in phosphate buffer.

### 3.3. Automatic ORAC Assay

Precision XS automating pipetting system was used for automated sample preparation, while the ORAC assay itself was performed based on the protocol reported by Huang et al. [24]. Trolox was used as a control standard. The experiments were performed at the temperature of 37 °C at the pH value of 7.4 with a blank sample in parallel. The FL600 microplate fluorescence reader was programmed to measure the fluorescence of fluorescein every minute upon adding the AAPH. All the measurements were expressed relative to the initial reading and blank and were performed in triplicate. Table 3 contains the structures of all the flavonoids used in this antioxidant study as well as their respective ORAC values.

### 3.4. QSAR model Development

#### 3.4.1. Molecular Descriptors

For development of quantitative structure–activity relationships (QSAR) models for prediction of antioxidant activity of flavonoids, an initial matrix of predictors was generated using quantum mechanical (QM) molecular descriptors. Two mechanisms of antioxidant activity were considered: (i) hydrogen atom transfer (HAT) [18], and (ii) sequential proton-loss electron-transfer (SPLET) mechanisms [20]. Three descriptors of the HAT mechanism, the minimum bond dissociation enthalpy (BDE_min_ of the first and second oxidation step) and the number of hydroxyl groups (*n*(OH)) were considered. Four descriptors of the SPLET mechanism, proton affinity (PA) and the electron transfer enthalpy (ETE) for two oxidation steps were further considered. Due to major differences in the values of these parameters in the gas phase and solvent, besides these descriptors, hydration energy (HE) was also considered. The enthalpies were defined with equations detailed in the Supporting Information of this paper. For the gas-phase calculations, at *ω*b97XD/6-311++G(d,p) level, the following enthalpy values (*H*) of proton and electron were used: *H*(H^+^) = 1.481 kcal mol^−1^ and *H*(*e*^−^) = 0.752 kcal mol^−1^ [25]. Similarly, for the case of water (based on SMD solvation method), the following values: *H*(H^+^) = −250.574 kcal mol^−1^ and *H*(e^−^) = −17.816 kcal mol^−1^ were employed. To calculate the solvated values of these constants, a model was built by “attaching” the proton or electron to one molecule of solvent (i.e., H_2_O monomer) [26]. The relative permittivity of water (*ε*_r_ = 78.4) was used for calculation [27].

#### 3.4.2. Conformational Analysis and Geometry Optimization

Conformational analysis was first performed for geometries of 36 flavonoids using molecular mechanics with the MMFF force field in Spartan 14 (Wavefunction, Inc., Irvine, CA, USA) [28,29]. Three steps were used in the conformational analysis: (i) torsion rotation, (ii) two correlated rotations to keep the rings closed, and (iii) a six-membered ring flip. Each of the steps was followed by energy minimization. Subsequently, all the generated conformers were optimized using the semi-empirical Austin model 1 (AM1) method [30]. Out of these conformers, 20 lowest-energy structures were optimized at HF/3-21G level of theory. Finally, five resulting lowest energy structures were fully optimized using density functional theory (DFT) with the *ω*b97XD functional and 6-311++G(d,p) basis set [17,31,32]. These calculations were first performed in the gas phase.

Conformational analyses in this approach were performed on the neutral, anionic and mono-radical species of all flavonoids, while the radical anion and diradical species were directly optimized at the highest level of theory. In addition to the gas-phase calculations, calculations were also performed in aqueous phase using the implicit SMD continuum solvation model [33]. Geometry optimizations were performed for radical and radical cation forms based on the lowest-enthalpy conformers of the flavonoids. All the DFT calculations were carried out using Gaussian 16 (Gaussian, Inc., Wallingford, CT, USA, Ref. S1 in SI) software.

#### 3.4.3. Selection of Molecular Descriptors for QSAR Modelling

Out of all the computed QM molecular descriptors, four (namely, BDE_min_ (1), ETE (1), PA (1), and HE) were a-priori selected to build ANN-based QSAR models. Although exhibiting quite strong correlation to Trolox-equivalent antioxidant capacity (TEAC), the number of hydroxyl groups, as expected, correlate poorly with the ORAC values [12]. According to Zhang et al., including this descriptor into the ORAC modelling process did not improve its predictive ability [19]. Similarly, poor correlations were exhibited between BDE_min_ (2), ETE (2), PA (2) and ORAC as well. Generally, diradical and diradical anion species are known to be energetically unfavorable, and thus unstable in solution. Given this nature, we have excluded the descriptors of the second oxidation step from the final QSAR model. Values of the four (BDE_min_ (1), ETE (1), PA (1), and HE) descriptors are summarized in Table 4.

### 3.5. QSAR Model Development

Prior to building all the models, the dependent variable, ORAC [34] was transformed to the natural logarithmic scale to reduce skewness. Then, partial least squares (PLS) was employed to build initial QSAR models. The dataset was uniformly divided into 25 training and 11 validation samples using the Kennard and Stone stratification algorithm [35]. The PLS model coefficients were fitted using the method of least squares.

Since relationships between molecular structure and antioxidant activity are complex, the linear PLS model does not account for the whole variability of ORAC values for the 36 investigated flavonoids. To tackle this problem, we developed another QSAR model using a non-linear machine learning method, namely artificial neural networks (ANNs) [36]. The ANN architecture was thoroughly optimized employing a grid search, i.e., the number of hidden neurons was varied in [2:1:100] and the training ratio in [60:1:75%]. Levenberg-Marquardt back-propagation algorithm was employed for training the ANNs. Recently, we have shown in a comprehensive study that for interpretation of ANN-QSAR models the partial derivative (PaD) method could be safely used without an experienced (bio)chemist present [12,15]. Thereby, the PaD method was utilized for analysis of the contribution of the input variables towards the output (ORAC).

### 3.6. QSAR Model Validation

In this study, leave-one-out cross-validation (LOO-CV) was employed for the internal validation of the PLS model, while the stratified validation set was used for its external validation. In that manner, predictions were made for observations that were not used within any segment of the modelling process. In the case of the ANN-based QSAR model, the dataset was randomly divided into a training set of 26, testing set of 5, and validation set of 5 compounds. The resulting neural networks were trained in 1000 replicated cycles (resampling with replacement), giving standard deviation values for all the predictions and errors. Five-fold CV (resampling without replacement) was also performed. However, it was found to be unsuitable for an ANN model with such a limited number of samples. Model performance metrics (root mean square error of estimation or prediction (RMSEE or RMSEP, Equation (4)) were subsequently computed. This allowed for obtaining genuinely reliable information about the predictive ability of the developed models [37,38].

(2) $$RMSEE/RMSEP=\sqrt{\frac{{\left(y\left(pred.\right)-y\left(obsd.\right)\right)}^{2}}{n}}$$

Chemical domain of applicability was also defined with two warning limits: (i) critical leverage (h*), and (ii) three multiples of standard deviation of standardized residuals. Leverage values represent the diagonal of the “hat” matrix and were obtained using the following equation:

(3) $$h=diag\left[X_{2}{}^{T}{\left(X_{1}{}^{T}X_{1}\right)}^{-1}X_{2}\right]$$

where X_1_ represents the predictor matrix for the training set, while X_2_ represents the predictor matrices for either training, testing, or validation sets [39]. Critical leverage value (h*) was computed using the following expression:

(4) h*=3(K+1)/N

where *K* represents the number of variables, while *N* represents the number of observations. All the chemometric computations were performed using MATLAB 2018b (MathWorks, Sherborn, MA, USA) software [39].

### 3.7. In-Silico Design of Potent Flavonoid-Based Antioxidants

Based on the ANN-QSAR model, potentially potent flavonoid-based antioxidants were designed. Using flavone as a template, hydrogens bound to aromatic carbons of A-, B-, and C-rings were exchanged with hydroxyl groups, at any three of its ten possible sites. This yielded 115 possible structures. Designed flavonoids were fully optimized using the DFT method at *ω*b97XD/6-311++G(d,p) level of theory, after which the four QM descriptors (BDE_min_ (1), ETE (1), PA (1), and HE) were calculated. The developed ANN-QSAR model was applied to predict their antioxidant activity values, and the corresponding structural trends were analysed.

### 3.8. Theoretical Methods

#### 3.8.1. Partial Least Squares (PLS)

PLS [13] is a form of linear regression between a dependent variable y and *k*-independent variables: *x*_1_, *x*_2_, …, *x*_k_. In PLS, the original X- and y- variables are compressed into latent variables (LVs) which represent their linear combinations. They are constructed in the direction of maximum variance of X- and y- and the maximum correlation between X- and y-. Since the few constructed LVs are then regressed against y, PLS performs simultaneous linear regression and dimension reduction. There are several algorithms to construct/extract LVs, the most widely used being the NIPALS and SIMPLS algorithms [13,40]. In this work, the SIMPLS algorithm was employed to extract the LVs used to build the models. Leave-one-out cross-validation (LOO-CV) was used to optimize the number of latent variables, while CV—analysis of variance (CV-ANOVA) was used for evaluation of the statistical significance of the resulting model [41]. Confidence intervals of the individual coefficients were computed using the *t*-statistic [37].

#### 3.8.2. Artificial Neural Networks (ANNs)

**Figure 5:**
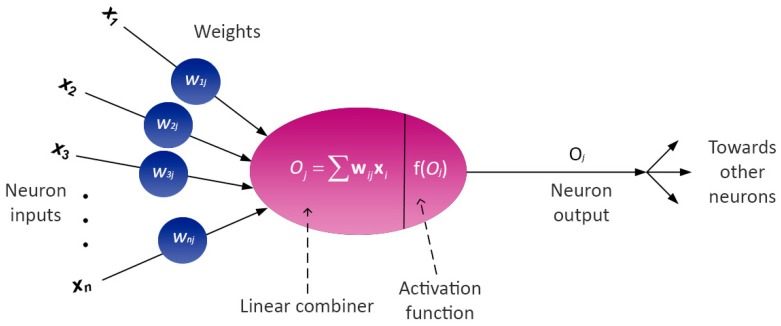
Schematic representation of an artificial neuron.

ANNs constitute machine learning methods by simulating the neural network of a living brain [36]. They consist of layers of neurons inter-connected by synapses. Here, a feed-forward multilayer perceptron (MLP) neural network with a back-propagation learning algorithm was used [42,43]. The MLP neural network (Figure 5) consists of an input layer, a hidden layer and an output layer, and this form was selected for regression because it is considered sufficient to be a universal function approximator [44].

Output of each of the neurons *j*, *O_j_*, is defined as:

(5) $$O_{j}=f\left(\sum w_{ij}O_{i}+b_{j}\right)$$

where *w_ij_* represent weights of the synapses, *O_i_* represents output of the previous neuron, *i*, while *b_j_* represents the bias term. These neuron outputs are inputs for the transfer function, and in the case of regression the input layer contains X-variables (predictors), the hidden layer contains neurons with a hyperbolic tangent sigmoid transfer function:

(6) $$\sigma \left(x\right)=\tanh x=\frac{e^{x}-e^{-x}}{e^{x}+e^{-x}}$$

while the output layer has neurons with a linear transfer combiner function. 

##### Partial Derivative (PaD) Method

Partial derivative (PaD) method was based on viewing the multilayer perceptron (MLP) ANN as a multivariate function consisting of vector inputs and scalar outputs. Developed by Dimopoulos, et al. it is used to analyse the changes of the output when infinitesimal changes are introduced to the inputs [15,45]. MLP function in the form of $f:{\mathbb{R}}^{2}\to \mathbb{R}$ was considered:

(7) $$y=f\left(X\right)=\Phi_{2}\left(W_{ho}\Phi_{1}\left(W_{ih}{X+b}_{1}\right){+b}_{2}\right)$$

where $\phi_{2}$ refers to the function in the output node, $\phi_{1}$ the activation function at hidden layer nodes, W*_ho_* and W*_ih_* the weight matrices between output and hidden layers, and between hidden and input layers, respectively, b_1_ and b_2_ the bias for hidden and output layers, and X referred to the inputs. Thereby, the first partial derivative of the output with respect to a particular input/descriptor was expressed as the following:

(8) $$d_{j}{}_{k}={\Phi_{2}}^{\prime }\left(O_{k}\right)\sum_{i=1}^{nh}\left(w_{io}{\Phi_{1}}^{\prime }H_{ik}w_{ji}\right)$$

where *O_k_* are the outputs of the hidden layer, *w_io_* are the weights for connections between the input and hidden layers, *w_ji_* are the weights for connections between the hidden and output layers, *i* = 1, …, *nh* represents the index of hidden layer neurons, *j* = 1, …, *M* the index of input variables, while *k* = 1, …, *N* represents the number of observations. *H*_ik_, the outputs of each hidden layer neuron are defined as:

(9) $$H_{ik}=\sum_{j=1}^{M}w_{ji}X_{jk}$$

where *X*_jk_ represents the matrix of input variables.

Obtained partial derivatives with respect to the input variables were analysed to detect the minute trends in the change of molecular descriptors. Their relative contributions with respect to the ORAC (output variable) were calculated as a sum of squared partial derivatives (SSD).

## 4. Conclusions

In conclusion, we developed PLS- and ANN-QSAR models which can be used for prediction of antioxidant activity of novel flavonoids or their derivatives. It has been extensively validated using an external validation set, with the ANN training repeated 1000 times obtaining standard deviations of both predictions and errors. The results have shown that as opposed to the linear PLS model, the ANN-based QSAR model was highly predictive, reliable, and robust. Since it is based on quantum mechanical molecular descriptors, it was easy to interpret using the PaD method. The interpretations revealed the strong non-linearity which resulted in a decrease in model error as compared to the PLS model. It confirmed the assumption that for flavonoids evaluated using the ORAC assay in solution, the prevalent mechanism is the SPLET mechanism, strongly preferred over the typical HAT mechanism. This finding is further supported by DFT calculations on the three possible reaction pathways of two model compounds, genistein and quercetin, with the peroxy radical generated by the ORAC essay. Both SPLET and SETPL mechanisms are found to be competitive and favored in aqueous medium. As the calculated solvent stabilization effect of the ion pair intermediate of both SPLET and SETPL mechanisms in aqueous medium is enormous, we believe hydration energy is also a critical factor in designing novel compounds with potent antioxidant activity. The ANN-QSAR calculations have also revealed the importance of the SETPL mechanism. However, the parameters of the SETPL mechanism were not considered for current QSAR modelling, which will be the object of future work. Based on the ANN results of this work, 115 flavone-based structures were further evaluated and their ORAC values were predicted. Consequently, some general guidelines were given for synthesis of novel flavonoid derivatives with potent antioxidant activities with an emphasis on R6, R7, R2′, and R3′ positions of the flavone moiety. However, a concern has been raised about this approach by an academic editor of IJMS on the non-suitability of capacity assays such as the ORAC assay for construction of Structure Activity Relationships. The rationale for this concern was that the ORAC assay provides the total number of radicals that can be scavenged by a molecule, but this does not only involve the parent compound, but also the oxidation products of the parent compound that are formed [46,47]. Although the ORAC assay does not measure the initial rates, the initial rate of the antioxidant reaction with ROO itself is a critical component in the ORAC values. To have a high ORAC value, the initial rates have to be high in the first place. ORAC assay measures the kinetics of the reaction until the fluorescence is completely quenched, if the reaction rate of the antioxidant with the RO2 radical is slow in the first place, the ORAC values will be low. Ultimately, validation of the predictions through experiments shall be conducted to assess power of the current model for prediction of ORAC values of newly designed compounds. However, to construct a model that can take into account of the secondary and tertiary reactions between ROS and flavonoids is nearly impossible because we do not know the primary/secondary reaction products of the ROS and the flavonoids.

## Figures and Tables

**Table 1:** Calculated (*ω*B97XD/6-311+G** level of theory) gas-phase and aqueous-phase reaction enthalpies (*ΔH_298_*, kcal/mol) of the hydrogen atom transfer (HAT), sequential proton-loss electron transfer (SPLET), and single electron transfer followed by proton loss (SETPL) reaction pathways for interaction of genistein and quercetin with peroxyl radical derived from 2,2′-azobis (2-amidino-propane) dihydrochloride (AAPH).

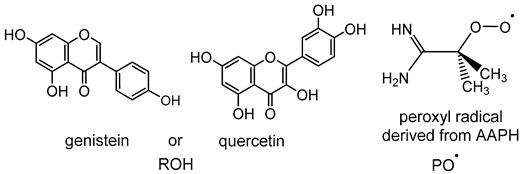				
	Genistein		Quercetin	
	Gas Phase	Water	Gas Phase	Water
**Overall reaction**				
ROH + PO^•^→RO^•^ + POH	5.6	0.8	−3.1	−5.6
**HAT mechanism**				
ROH→RO^•^ + H^•^	83.8	85.3	75.1	78.9
PO^•^ + H^•^→POH	−78.2	−84.5	−78.2	−84.5
**SPLET mechanism**				
ROH + PO^•^→RO^−^ + POH^•+^	134.2	32.9	126.8	31.9
RO^−^ + POH^•^^+^→RO^•^ + POH	−128.6	−32.1	−129.8	−37.5
**SETPL mechanism**				
ROH + PO^•^→ROH^•+^ + PO^−^	144.6	32.6	137.6	28.0
ROH^•+^ + PO^−^→RO^•^ + POH	−138.9	−31.7	−140.7	−33.6

**Table 2:** Frequency of occurrence of OH groups for the top 55 (ORAC > 5) and bottom 18 (ORAC < 2) designed compounds.

R1	R2	R3	R4	R5	R6	R7	R8	R1′	R2′	R3′	R4′	R5′	R6′
Top 55 compounds (ORAC > 5)													
0	0	**11**	1	7	**20**	**19**	**17**	0	**20**	**20**	**18**	**16**	7
Bottom 18 compounds (ORAC < 2)													
0	0	0	**5**	0	**9**	**5**	0	2	0	**5**	2	3	2

The most prominent OH groups are denoted in bold.

**Table 3:** List of flavonoids, their molecular structures ^a^ and determined oxygen radical absorbance capacity (ORAC) values. ANN: artificial neural network.

#	Name	Molecular Structure	ln (ORAC)	PLS	ANN
1	Genistein	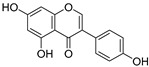	2.267 ± 0.008	0.953	2.165 ± 0.101
2	Naringenin	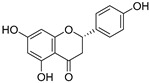	2.141 ± 0.014	0.939	2.152 ± 0.072
3	Scutellarin	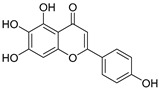	2.042 ± 0.014	1.567	2.171 ± 0.216
4	3,5,7,8,3′,4′-Hexahydroxyflavone	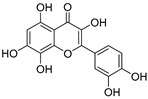	2.026 ± 0.001	1.944	2.038 ± 0.216
5	Epicatechin	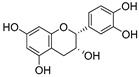	2.018 ± 0.004	1.579	1.326 ± 0.222
6	Kaempferol	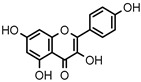	2.018 ± 0.018	1.331	1.836 ± 0.085
7	Eriodictyol	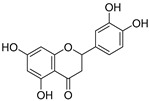	2.013 ± 0.006	1.473	1.962 ± 0.098
8	Apigenin	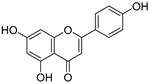	2.010 ± 0.000	0.796	1.968 ± 0.097
9	Quercetin	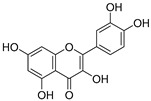	1.970 ± 0.003	1.525	1.979 ± 0.059
10	Liquiritigenin	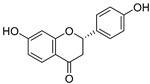	1.970 ± 0.003	0.948	2.062 ± 0.081
11	Fisetin	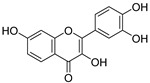	1.959 ± 0.022	1.368	1.798 ± 0.060
12	Taxifolin	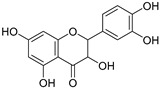	1.942 ± 0.004	1.570	1.922 ± 0.081
13	Hesperetin	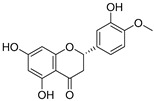	1.938 ± 0.014	1.239	2.035 ± 0.062
14	3,3′,4′-Trihydroxyflavone	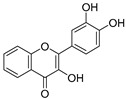	1.869 ± 0.019	1.143	1.873 ± 0.033
15	7,3′,4′-Trihydroxyflavone	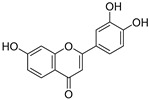	1.691 ± 0.016	1.368	1.658 ± 0.068
16	Diosmetin	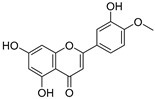	1.656 ± 0.001	1.069	1.728 ± 0.066
17	Luteolin	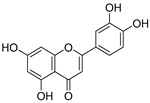	1.611 ± 0.013	1.322	1.730 ± 0.065
18	Morin	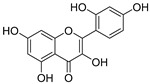	1.517 ± 0.006	1.424	1.588 ± 0.095
19	Epigallocatechin	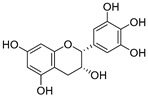	1.225 ± 0.020	1.932	1.227 ± 0.101
20	5,3′,4′-Trihydroxyflavone	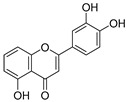	1.223 ± 0.038	1.141	1.206 ± 0.041
21	Ampelopsin	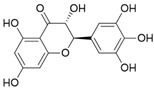	1.204 ± 0.038	1.842	1.245 ± 0.080
22	Myricetin	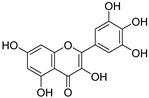	1.173 ± 0.000	1.721	1.248 ± 0.077
23	Wogonin	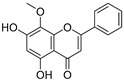	1.077 ± 0.000	0.697	0.924 ± 0.137
24	7,8-Dihydroxyflavone	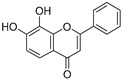	1.051 ± 0.002	1.322	1.097 ± 0.112
25	Chrysin	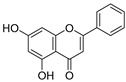	1.016 ± 0.001	0.226	1.051 ± 0.068
26	Pinocembrin	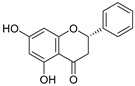	1.013 ± 0.011	0.225	0.978 ± 0.105
27	Catechin	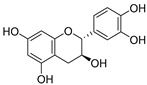	1.012 ± 0.018	1.597	1.266 ± 0.243
28	Eupatilin	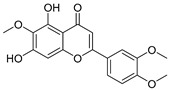	0.891 ± 0.013	0.556	0.799 ± 0.119
29	Baicalein	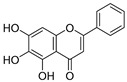	0.816 ± 0.003	1.382	0.730 ± 0.139
30	Pectolinarigenin	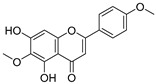	0.788 ± 0.023	0.515	0.832 ± 0.084
31	3,5-Dihydroxyflavone	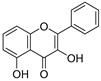	0.767 ± 0.046	0.846	0.761 ± 0.095
32	Alpinetin	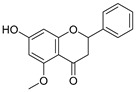	0.492 ± 0.009	0.410	0.505 ± 0.095
33	Galangin	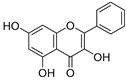	0.328 ± 0.030	1.063	0.539 ± 0.123
34	Genkwanin	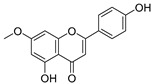	−0.072 ± 0.058	0.667	−0.031 ± 0.131
35	Primuletin	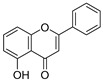	−0.969 ± 0.004	0.044	−1.055 ± 0.164
36	Tectochrysin	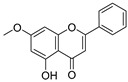	−1.581 ± 0.079	−1.306	−1.575 ± 0.247

^a^ Optimized geometries (*ω*B97XD/6-311++G**) are given in Figure S6.

**Table 4:** Summary of calculated quantum mechanical parameters of the two considered antioxidant mechanisms.

#	Compound Name	n (OH)	ETE (1)	PA (1)	BDE_min_ (1)	HE
1	Genistein	3	89.175	33.630	83.987	−17.606
2	Naringenin	3	90.557	33.240	84.979	−19.251
3	Scutellarin	4	78.864	35.648	75.694	−17.513
4	3,5,7,8,3′,4′-Hexahydroxyflavone	6	67.389	42.831	71.402	−20.148
5	Epicatechin	5	68.664	49.233	79.080	−27.271
6	Kaempferol	4	84.259	33.472	78.913	−17.382
7	Eriodictyol	4	84.665	33.190	79.037	−21.559
8	Apigenin	3	89.514	35.499	86.196	−18.219
9	Quercetin	5	83.812	32.554	77.549	−19.749
10	Liquiritigenin	2	89.619	33.975	84.776	−19.203
11	Fisetin	4	84.471	33.907	79.560	−19.860
12	Taxifolin	5	85.166	32.360	78.708	−23.422
13	Hesperetin	3	85.930	33.240	80.352	−17.828
14	3,3′,4′-Trihydroxyflavone	3	73.742	43.939	78.863	−13.813
15	7,3′,4′-Trihydroxyflavone	3	84.339	34.749	80.271	−21.503
16	Diosmetin	3	87.176	33.782	82.141	−16.976
17	Luteolin	4	84.601	34.584	80.367	−20.391
18	Morin	5	70.199	47.284	78.665	−21.759
19	Epigallocatechin	6	78.699	35.785	75.666	−27.626
20	5,3′,4′-Trihydroxyflavone	3	84.893	34.572	80.647	−15.957
21	Ampelopsin	6	82.652	32.448	76.283	−25.878
22	Myricetin	6	81.442	33.218	75.843	−21.695
23	Wogonin	2	89.929	33.535	84.646	−11.865
24	7,8-Dihydroxyflavone	2	84.537	32.175	77.894	−14.759
25	Chrysin	2	96.442	33.750	91.375	−13.037
26	Pinocembrin	2	97.500	33.197	91.879	−13.970
27	Catechin	5	68.136	49.725	79.043	−27.785
28	Eupatilin	2	92.534	33.579	87.295	−13.554
29	Baicalein	3	81.439	33.164	75.786	−12.133
30	Pectolinarigenin	2	92.425	33.712	87.319	−12.482
31	3,5-Dihydroxyflavone	2	83.211	36.125	80.518	−7.727
32	Alpinetin	1	95.985	33.690	90.857	−17.037
33	Galangin	3	85.569	33.430	80.181	−12.605
34	Genkwanin	2	89.997	35.002	86.181	−14.537
35	Primuletin	1	89.804	40.053	91.039	−8.315
36	Tectochrysin	1	108.611	39.485	109.278	−9.220

Thermodynamic quantum mechanical (QM) parameters (ETE (1), PA (1), BDE_min_ (1), and HE) are expressed in kcal mol^−1^. The index one refers to the first oxidation step. All the abbreviations explained in the text.

## Supplementary Materials

Supplementary materials can be found at https://www.mdpi.com/1422-0067/20/9/2328/s1.
