# Supplementary material for: Non-Linear Quantitative Structure–Activity Relationships Modelling, Mechanistic Study and In-Silico Design of Flavonoids as Potent Antioxidants
Source: Int J Mol Sci. 2019 May 10;20(9):2328. doi: 10.3390/ijms20092328 (PMC6539043; doi:10.3390/ijms20092328)
Supplement: Supplementary file 1 [file ijms-20-02328-s001.pdf]

Article

Non-Linear Quantitative Structure–Activity Relationships Modelling, Mechanistic Study and In-Silico Design of Flavonoids as Potent Antioxidants

Petar Žuvela [1], Jonathan David [1], Xin Yang [2], Dejian Huang [2] and Ming Wah Wong [1,*]

[1] Department of Chemistry, National University of Singapore, 3 Science Drive 3, Singapore 117543, Singapore; petar.zuvela@nus.edu.sg (P.Ž.); jonathan.david14@sps.nus.edu.sg (J.D.)

[2] Food Science and Technology Program, Department of Chemistry, National University of Singapore, 3 Science Drive 3, Singapore 117543, Singapore; xinyang@u.nus.edu (X.Y.); chmhdj@nus.edu.sg (D.J.)

* Correspondence: chmwmw@nus.edu.sg; Tel.: +65-651-64-320; Fax: +65-677-91-691

Contents:

1. **Supplementary reference 1 (S1).** Full Gaussian 16 reference.
2. Equations describing the parameters of the HAT and SPLET mechanisms of antioxidant activity.
3. **Figure S1.** Schematic representation of three prominent antioxidant activity mechanisms of the ORAC assay. HAT stands for the hydrogen atom transfer, SPLET for the sequential proton-loss electron transfer, while SETPL stands for single electron transfer followed by proton loss mechanism.
4. **Figure S2.** Optimization of the number of PLS latent variables. The optimal number (two) is denoted with an orange cross.
5. **Figure S3.** Predictive ability of the cross-validated (using leave-one-out cross-validation) PLS-based QSAR model.
6. **Figure S4.** Predictive ability of the cross-validated (using five-fold cross-validation) ANN-based QSAR model.
7. **Figure S5.** Leverage values of the 115 flavonoids designed using a combinatorial approach.
8. **Figure S6.** Optimized geometries (ωB97XD/6-311+G**) of 36 flavonoids.
9. **Table S1.** List of 115 flavonoids designed using a combinatorial approach and calculated quantum mechanical parameters of the two considered antioxidant mechanisms.
10. **Table S2.** Cartesian coordinates of optimized geometries (ωB97XD/6-311+G**) for the reactions of genistein/quercetin (ROH) with peroxyl radical derived from AAPH (PO•) as depicted in **Table 3** of the main text.
11. **Table S3.** Cartesian coordinates of optimized geometries (ωB97XD/6-311+G**) of 36 flavonoids involved in the QSAR modelling. Total energy (E) in Hartrees. N_i is the number of imaginary frequencies.
12. **Table S4.** Cartesian coordinates of optimized geometries (ωB97XD/6-311+G**) of 115 combinatorially-designed flavonoids. Total energy (E) in Hartrees. N_i is the number of imaginary frequencies.

Ref S1. Gaussian 16, Revision B.01, Frisch, M. J.; Trucks, G. W.; Schlegel, H. B.; Scuseria, G. E.; Robb, M. A.; Cheeseman, J. R.; Scalmani, G.; Barone, V.; Petersson, G. A.; Nakatsuji, H.; Li, X.; Caricato, M.; Marenich, A. V.; Bloino, J.; Janesko, B. G.; Gomperts, R.; Mennucci, B.; Hratchian, H. P.; Ortiz, J. V.; Izmaylov, A. F.; Sonnenberg, J. L.; Williams-Young, D.; Ding, F.; Lipparini, F.; Egidi, F.; Goings, J.; Peng, B.; Petrone, A.; Henderson, T.; Ranasinghe, D.; Zakrzewski, V. G.; Gao, J.; Rega, N.; Zheng, G.; Liang, W.; Hada, M.; Ehara, M.; Toyota, K.; Fukuda, R.; Hasegawa, J.; Ishida, M.; Nakajima, T.; Honda, Y.; Kitao, O.; Nakai, H.; Vreven, T.; Throssell, K.; Montgomery, J. A., Jr.; Peralta, J. E.; Ogliaro, F.; Bearpark, M. J.; Heyd, J. J.; Brothers, E. N.; Kudin, K. N.; Staroverov, V. N.; Keith, T. A.; Kobayashi, R.; Normand, J.; Raghavachari, K.; Rendell, A. P.; Burant, J. C.; Iyengar, S. S.; Tomasi, J.; Cossi, M.; Millam, J. M.; Klene, M.; Adamo, C.; Cammi, R.; Ochterski, J. W.; Martin, R. L.; Morokuma, K.; Farkas, O.; Foresman, J. B.; Fox, D. J. Gaussian, Inc., Wallingford CT, 2016.

Equations describing the parameters of the HAT and SPLET mechanisms of antioxidant activity. The enthalpies (bond dissociation enthalpy – BDE, electron transfer enthalpy – ETE, and proton affinity – PA) were defined as follows (n = number of hydroxyl groups, $1 \le k \le n$):

$$BDE_k = H(Ar(OH)_{n-k}(O^{\bullet})_k) + H(H^{\bullet}) - H(Ar(OH)_{n-k+1}(O^{\bullet})_{k-1}) \quad (1)$$

$$ETE_k = H(Ar(OH)_{n-k}(O^{\bullet})_k) + H(e^-) - H([Ar(OH)_{n-k}(O^{\bullet})_{k-1}O:]^-) \quad (2)$$

$$PA_k = H([Ar(OH)_{n-k}(O^{\bullet})_{k-1}O:)]^- + H(H^+) - H(Ar(OH)_{n-k+1}) \quad (3)$$

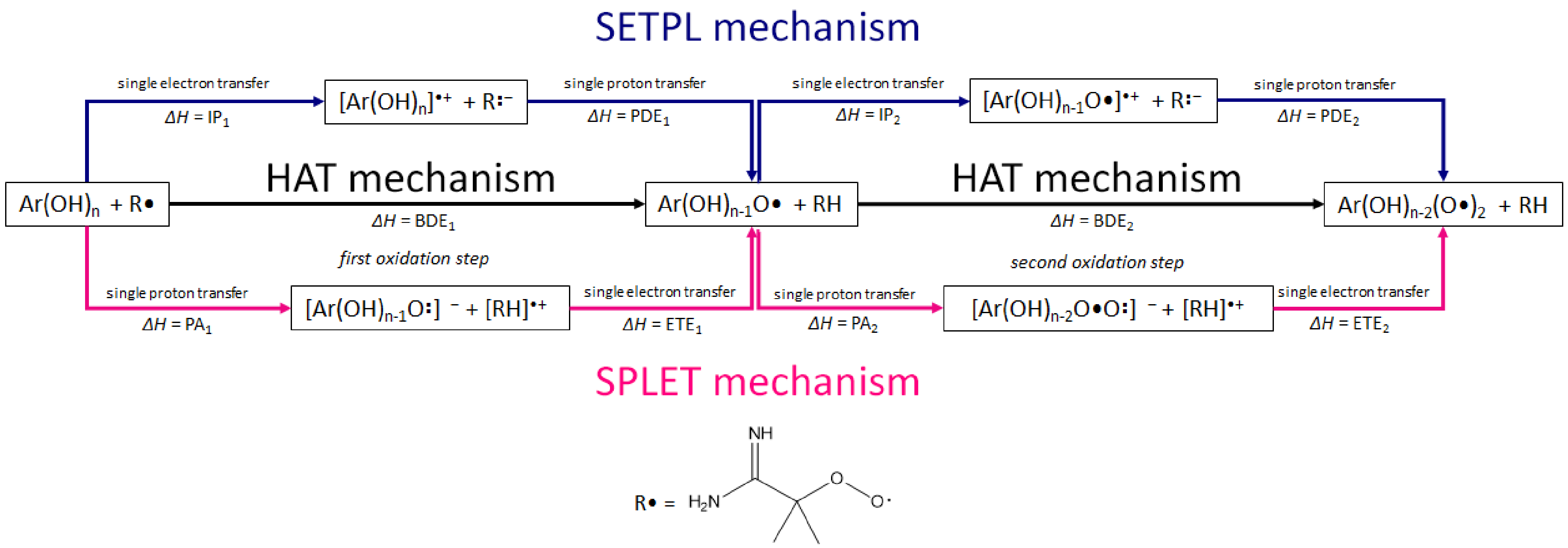

Figure S1. Schematic representation of three prominent antioxidant activity mechanisms of the ORAC assay. HAT stands for the hydrogen atom transfer, SPLET for the sequential proton-loss electron transfer, while SETPL stands for single electron transfer followed by proton loss mechanism.

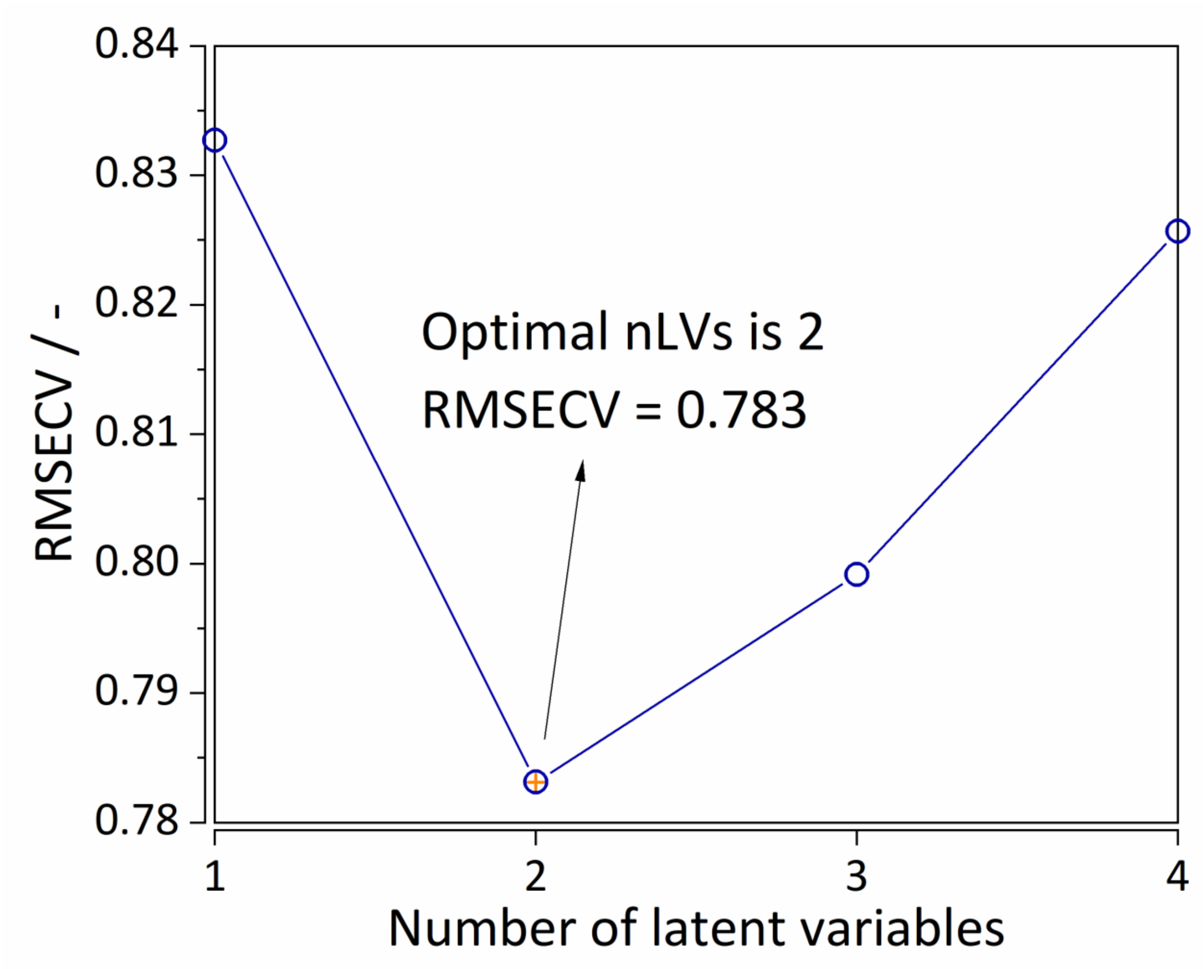

Figure S2. Optimization of the number of partial least squares latent variables. The optimal number (two) is denoted with an orange cross.

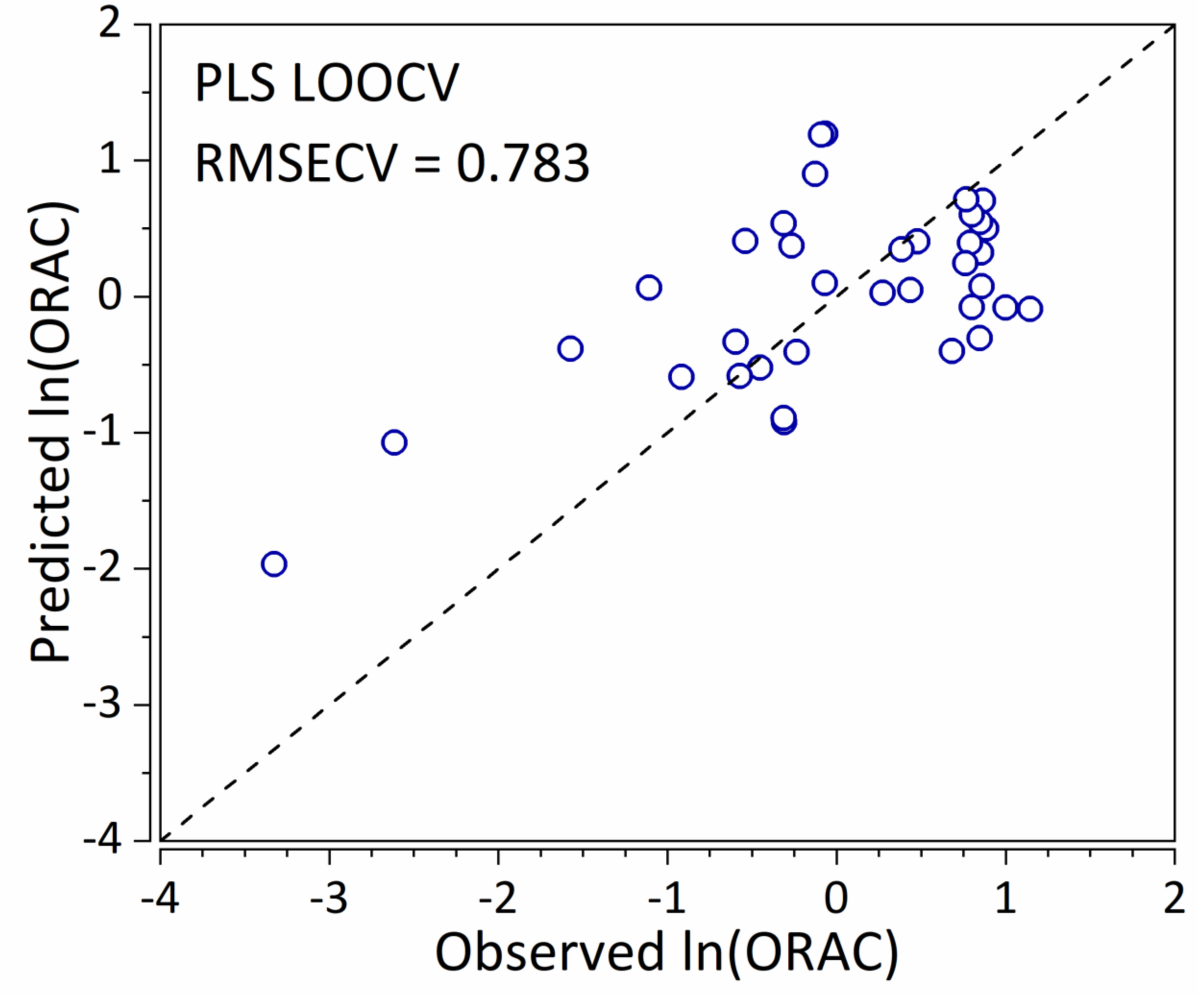

Figure S3. Predictive ability of the cross-validated (using leave-one-out cross-validation) PLS-based quantitative structure–activity relationships (QSAR) model.

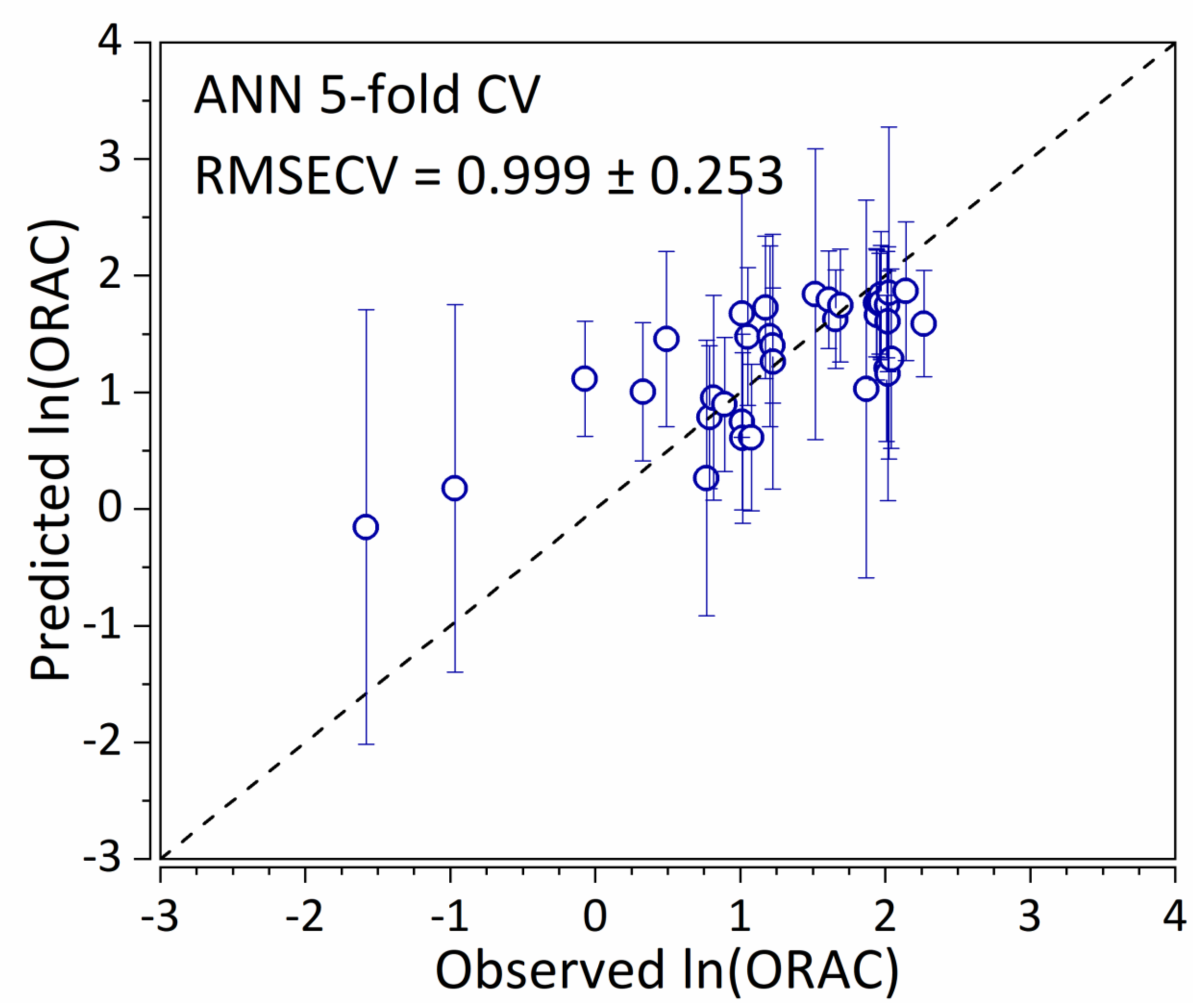

Figure S4. Predictive ability of the cross-validated (using five-fold cross-validation) artificial neural network (ANN)-based QSAR model.

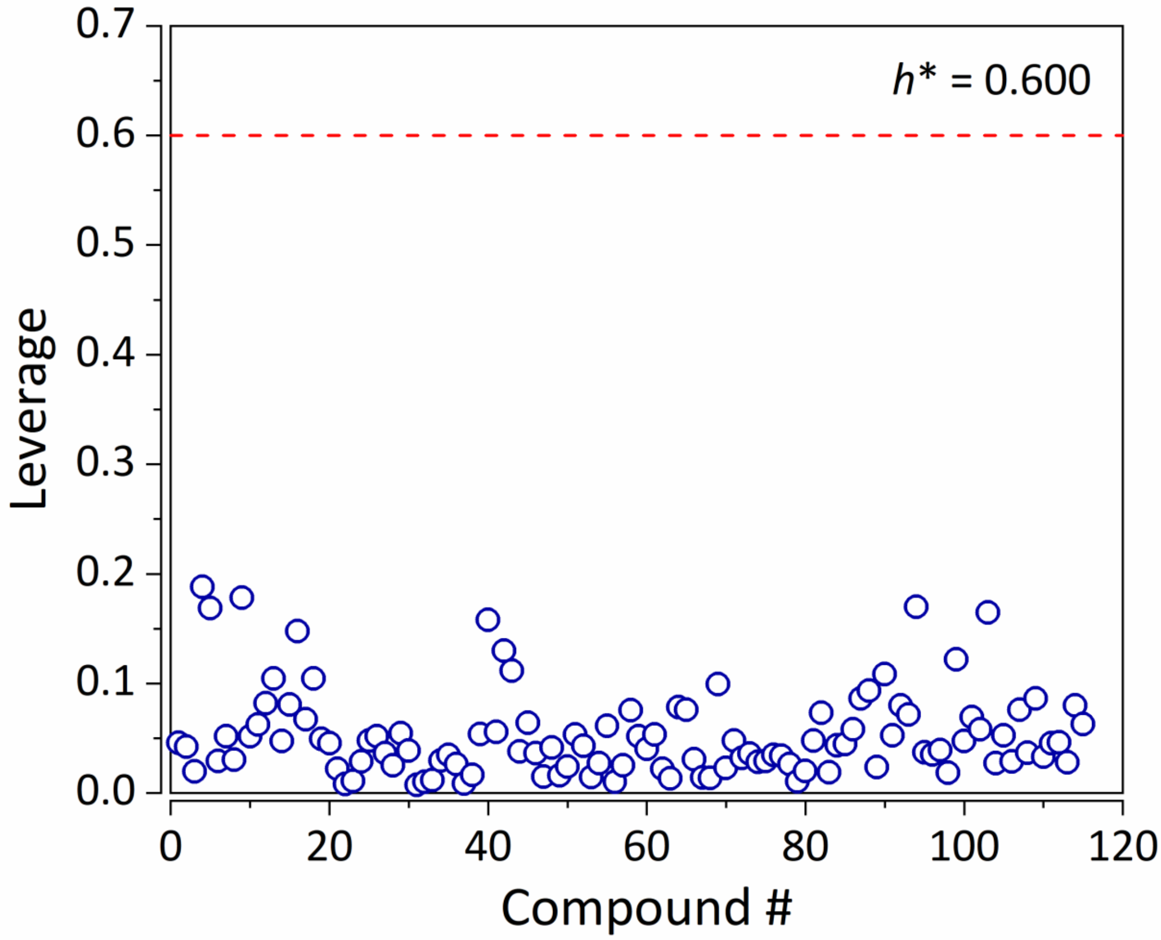

Figure S5. Leverage values of the 115 flavonoids designed using a combinatorial approach.

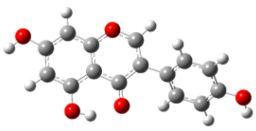

1

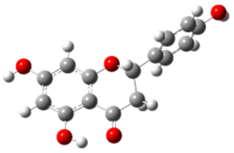

2

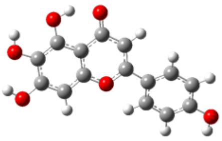

3

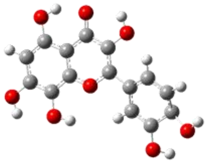

4

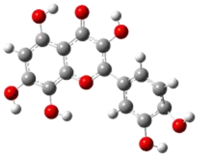

5

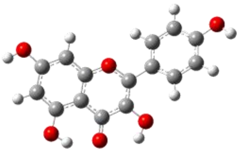

6

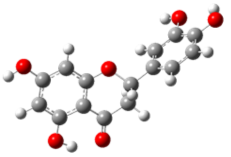

7

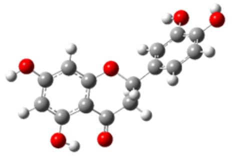

8

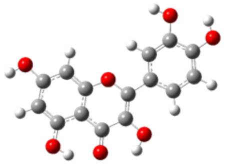

9

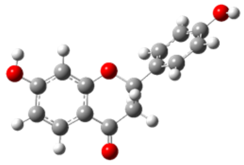

10

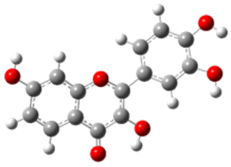

11

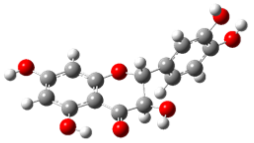

12

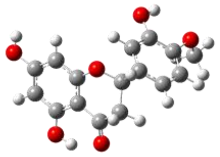

13

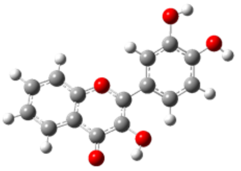

14

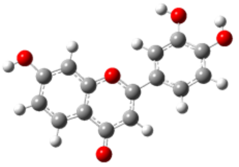

15

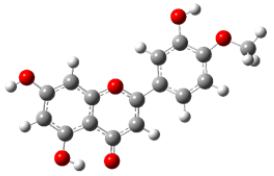

16

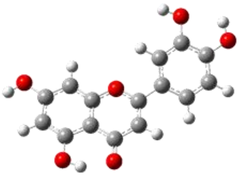

17

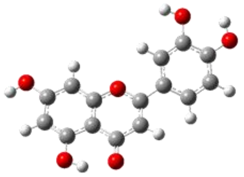

18

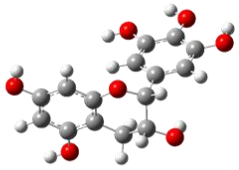

19

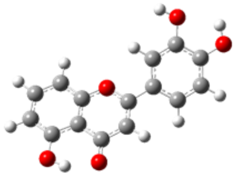

20

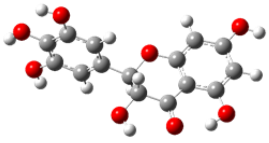

21

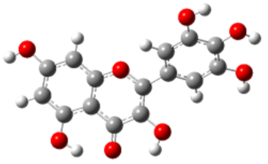

22

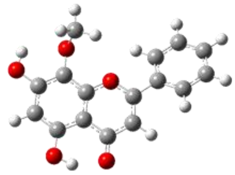

23

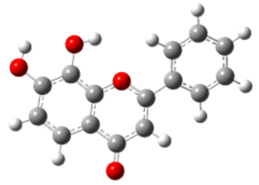

24

Figure S6. Optimized geometries (ωB97XD/6-311+G**) of 36 flavonoids.

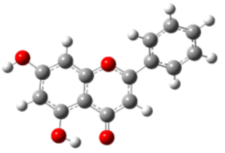

25

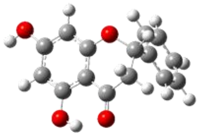

26

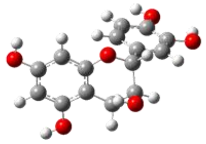

27

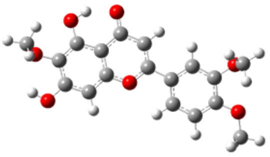

28

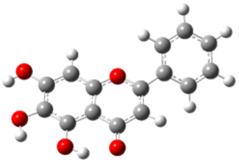

29

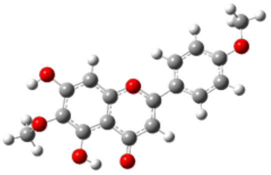

30

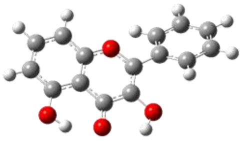

31

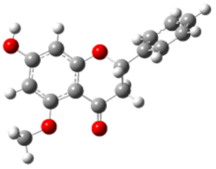

32

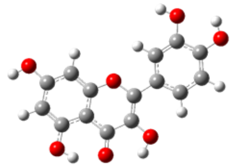

33

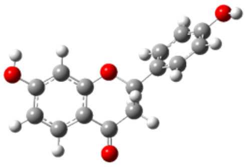

34

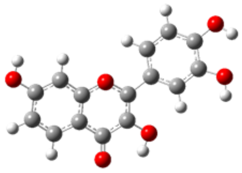

35

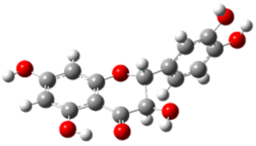

36

Figure S6. (cont.)

Table S1. List of 115 flavonoids designed using a combinatorial approach and calculated quantum mechanical parameters of the two considered antioxidant mechanisms.

#	R1	R2	R3	R4	R5	R6	R7	R8	R1'	R2'	R3'	R4'	R5'	R6'	*n*(OH)	ETE(1)	PA(1)	$BDE_{min}(1)$	HE	ln(ORAC)	ORAC
1	H	H	H	H	H	H	H	OH	H	H	OH	OH	H	H	3	86.059	32.448	81.032	-19.988	2.454	11.637
2	H	H	H	H	H	H	OH	OH	H	OH	H	H	H	H	3	85.372	31.407	79.303	-18.638	2.451	11.603
3	H	H	H	H	H	OH	H	H	H	OH	H	OH	H	H	3	87.056	37.591	87.171	-22.490	2.439	11.467
4	H	H	H	H	H	H	H	H	H	H	OH	OH	OH	H	3	83.880	30.484	76.888	-18.857	2.386	10.865
5	H	H	H	H	H	H	H	OH	H	OH	OH	H	H	H	3	85.465	32.135	80.124	-18.622	2.378	10.787
6	H	H	H	H	H	H	OH	OH	H	H	OH	H	H	H	3	84.458	32.382	79.364	-19.773	2.281	9.784
7	H	H	H	H	H	OH	OH	H	H	OH	H	H	H	H	3	86.983	33.352	82.858	-18.691	2.259	9.574
8	H	H	H	H	H	OH	H	OH	H	OH	H	H	H	H	3	88.851	32.785	84.160	-20.605	2.219	9.202
9	H	H	H	H	H	H	OH	OH	H	H	H	OH	H	H	3	84.210	32.455	79.188	-20.236	2.204	9.057
10	H	H	H	H	H	OH	H	H	H	OH	OH	H	H	H	3	85.096	33.237	80.857	-19.449	2.154	8.619
11	H	H	H	H	H	OH	H	H	H	H	OH	H	OH	H	3	86.784	37.637	86.945	-24.768	2.144	8.530
12	H	H	H	H	H	H	OH	H	H	H	OH	H	OH	H	3	87.599	37.512	87.636	-24.208	2.092	8.099
13	H	H	H	H	H	H	OH	H	H	H	OH	OH	H	H	3	85.448	32.717	80.689	-21.691	2.089	8.076
14	H	H	H	H	OH	H	H	OH	H	H	H	OH	H	H	3	78.758	37.392	78.674	-17.839	2.060	7.848
15	H	H	H	H	H	H	OH	H	H	OH	OH	H	H	H	3	84.837	33.450	80.811	-19.010	2.057	7.820
16	H	H	H	H	OH	H	H	OH	H	H	OH	H	H	H	3	79.216	37.143	78.884	-17.262	2.056	7.813
17	H	H	H	H	H	H	H	H	H	OH	H	H	OH	OH	3	77.230	35.919	75.673	-18.741	2.037	7.667
18	H	H	H	H	H	OH	OH	H	H	H	OH	H	H	H	3	86.409	33.587	82.520	-20.876	2.020	7.539
19	H	H	H	H	H	H	H	OH	H	OH	H	OH	H	H	3	88.479	33.642	84.644	-20.532	2.006	7.430
20	H	H	H	H	H	OH	H	H	H	H	OH	OH	H	H	3	85.763	32.702	80.989	-22.326	1.980	7.239
21	H	H	H	H	H	OH	OH	H	H	H	H	OH	H	H	3	86.091	33.680	82.295	-21.060	1.950	7.031
22	H	H	H	H	OH	H	H	OH	H	OH	H	H	H	H	3	79.951	36.234	78.709	-15.724	1.938	6.944
23	H	H	H	H	H	H	H	H	H	OH	H	H	OH	H	2	80.370	38.289	81.183	-16.872	1.914	6.783
24	H	H	H	H	H	H	H	H	H	H	OH	H	OH	H	2	87.660	37.605	87.789	-19.166	1.914	6.780

*QM parameters (ETE(1), PA(1), BDEmin(1), and HE) are expressed in kcal mol^{-1}. The index one refers to the first oxidation step.
**All the abbreviations explained in the main text.

Table S1. (cont.)

#	R1	R2	R3	R4	R5	R6	R7	R8	R1'	R2'	R3'	R4'	R5'	R6'	*n*(OH)	ETE(1)	PA(1)	$BDE_{min}(1)$	HE	ln(ORAC)	ORAC
26	H	H	H	H	H	H	OH	H	H	H	H	OH	H	H	2	88.671	35.611	86.806	-19.013	1.911	6.761
27	H	H	H	H	H	OH	H	H	H	H	H	OH	H	H	2	86.377	37.862	86.763	-19.780	1.906	6.727
28	H	H	H	H	H	OH	OH	OH	H	H	H	H	H	H	3	88.302	28.686	79.513	-17.657	1.899	6.681
29	H	H	H	H	OH	H	H	H	H	OH	H	H	OH	H	3	80.680	38.167	81.371	-16.482	1.897	6.664
30	H	H	OH	H	H	H	H	OH	H	H	H	OH	H	H	3	80.969	37.585	81.078	-16.021	1.890	6.621
31	H	H	OH	H	H	OH	H	H	H	H	H	OH	H	H	3	80.200	38.392	81.116	-18.337	1.888	6.605
32	H	H	H	H	H	H	H	OH	H	OH	H	H	OH	H	3	80.701	37.656	80.881	-20.615	1.879	6.544
33	H	H	H	H	H	OH	H	H	H	OH	H	H	OH	H	3	80.246	38.321	81.091	-22.349	1.860	6.424
34	H	H	H	H	H	OH	H	H	H	H	OH	H	H	H	2	86.689	37.724	86.937	-19.404	1.857	6.403
35	H	H	H	H	H	H	OH	H	H	OH	H	H	OH	H	3	80.130	38.400	81.054	-21.940	1.841	6.303
36	H	H	H	H	OH	OH	H	OH	H	H	H	H	H	H	3	78.695	36.270	77.489	-14.056	1.832	6.245
37	H	H	OH	H	H	H	OH	H	H	H	H	H	OH	H	3	82.200	37.501	82.225	-18.144	1.812	6.120
38	H	H	H	H	H	OH	H	OH	H	H	H	H	H	H	2	87.926	34.028	84.478	-17.375	1.803	6.070
39	H	H	H	H	H	H	H	H	H	OH	OH	H	OH	H	3	78.485	34.605	75.614	-18.595	1.802	6.062
40	H	H	H	H	OH	H	H	H	H	H	OH	H	OH	H	3	87.892	37.438	87.854	-18.694	1.798	6.037
41	H	H	H	H	H	H	H	H	H	OH	H	OH	OH	H	3	79.469	35.415	77.408	-19.433	1.797	6.029
42	H	H	OH	H	H	OH	H	H	H	H	H	H	OH	H	3	82.441	37.598	82.562	-18.524	1.785	5.962
43	H	H	H	OH	H	H	OH	OH	H	H	H	H	H	H	3	78.133	33.916	74.573	-14.781	1.767	5.856
44	H	H	H	H	OH	H	OH	H	H	H	H	OH	H	H	3	89.428	35.301	87.253	-18.055	1.758	5.801
45	H	H	H	H	H	H	OH	H	H	OH	H	H	H	OH	3	88.074	35.484	86.082	-21.229	1.753	5.771
46	H	H	OH	H	H	H	H	H	H	H	OH	H	OH	H	3	83.177	37.158	82.859	-18.335	1.748	5.744
47	H	H	H	H	H	H	OH	H	H	H	OH	H	H	H	2	87.055	38.328	87.908	-18.833	1.726	5.619
48	H	H	OH	H	H	OH	H	OH	H	H	H	H	H	H	3	82.607	37.332	82.463	-16.338	1.723	5.599
49	H	H	OH	H	H	OH	OH	H	H	H	H	H	H	H	3	81.359	38.176	82.059	-14.926	1.712	5.541
26	H	H	H	H	H	H	OH	H	H	H	H	OH	H	H	2	88.671	35.611	86.806	-19.013	1.911	6.761

*QM parameters (ETE(1), PA(1), BDEmin(1), and HE) are expressed in kcal mol^{-1}. The index one refers to the first oxidation step.
**All the abbreviations explained in the main text.

Table S1. (cont.)

#	R1	R2	R3	R4	R5	R6	R7	R8	R1'	R2'	R3'	R4'	R5'	R6'	*n*(OH)	ETE(1)	PA(1)	$BDE_{min}(1)$	HE	ln(ORAC)	ORAC
50	H	H	H	H	H	H	H	H	H	H	OH	OH	H	H	2	85.782	32.494	80.800	-16.511	1.710	5.531
51	H	H	H	H	H	H	H	H	H	OH	H	OH	H	OH	3	89.872	35.342	87.737	-20.666	1.694	5.441
52	H	H	OH	H	H	H	H	H	H	H	H	H	H	OH	2	91.104	29.154	82.782	-13.667	1.640	5.155
53	H	H	OH	H	H	OH	H	H	H	H	H	H	H	OH	3	88.902	29.919	81.344	-15.598	1.631	5.107
54	H	H	H	H	H	OH	H	H	H	OH	H	H	H	OH	3	88.145	35.486	86.155	-21.685	1.623	5.068
55	H	H	OH	H	H	H	H	H	H	H	OH	H	H	OH	3	89.102	29.987	81.613	-15.214	1.622	5.062
56	H	H	H	H	OH	H	H	H	H	H	OH	OH	H	H	3	86.385	32.008	80.916	-15.993	1.598	4.943
57	H	H	H	H	H	H	H	H	H	OH	OH	OH	H	H	3	82.008	33.355	77.887	-16.316	1.578	4.845
58	H	H	H	H	H	H	H	OH	H	OH	H	H	H	OH	3	88.218	35.098	85.839	-21.599	1.568	4.796
59	H	H	OH	H	H	H	OH	H	H	H	H	H	H	OH	3	88.514	29.945	80.983	-15.199	1.561	4.766
60	H	H	OH	H	H	H	H	H	H	H	H	H	OH	OH	3	90.509	28.816	81.849	-12.070	1.553	4.724
61	H	H	OH	H	H	H	H	OH	H	H	H	H	H	OH	3	89.648	29.363	81.535	-12.955	1.535	4.642
62	H	H	H	H	OH	H	OH	H	H	H	OH	H	H	H	3	87.322	38.247	88.093	-18.012	1.535	4.640
63	H	H	H	H	H	H	H	OH	H	H	H	OH	H	H	2	87.450	35.206	85.180	-17.504	1.532	4.629
64	H	H	OH	H	H	H	H	OH	H	H	H	H	OH	H	3	83.671	36.524	82.719	-16.041	1.497	4.467
65	H	H	OH	H	OH	H	H	H	H	H	H	H	H	OH	3	89.875	28.582	80.982	-10.372	1.405	4.077
66	H	H	H	H	OH	OH	H	H	H	H	H	OH	H	H	3	83.755	36.793	83.071	-15.515	1.401	4.058
67	H	H	H	H	H	H	H	OH	H	H	OH	H	OH	H	3	87.717	35.136	85.377	-22.299	1.399	4.049
68	H	H	OH	H	H	H	H	H	H	H	H	OH	H	OH	3	87.212	29.891	79.627	-15.129	1.396	4.040
69	H	H	OH	H	H	H	H	H	H	H	H	H	H	OH	2	89.155	29.678	81.357	-9.953	1.390	4.014
70	H	H	H	H	H	OH	H	OH	H	H	OH	H	H	H	3	87.980	34.116	84.620	-22.341	1.374	3.949
71	H	H	H	H	OH	H	H	OH	H	H	H	H	H	H	2	78.908	37.225	78.657	-12.375	1.361	3.899
72	H	H	H	H	H	H	H	OH	H	H	OH	H	H	H	2	87.670	35.172	85.365	-17.018	1.355	3.877
73	H	H	OH	H	H	OH	H	H	H	H	H	H	H	H	2	81.779	38.073	82.377	-13.477	1.313	3.716
74	H	H	H	H	H	H	H	H	H	OH	H	OH	H	H	2	90.735	35.359	88.618	-16.904	1.302	3.678

*QM parameters (ETE(1), PA(1), BDEmin(1), and HE) are expressed in kcal mol^{-1}. The index one refers to the first oxidation step.
**All the abbreviations explained in the main text.

Table S1. (cont.)

#	R1	R2	R3	R4	R5	R6	R7	R8	R1'	R2'	R3'	R4'	R5'	R6'	*n*(OH)	ETE(1)	PA(1)	$BDE_{min}(1)$	HE	ln(ORAC)	ORAC
75	H	H	H	H	H	OH	H	H	H	OH	H	H	H	H	2	87.362	37.396	87.281	-17.180	1.301	3.674
76	H	H	OH	H	H	H	H	H	H	H	H	OH	OH	H	3	84.029	33.177	79.730	-15.632	1.277	3.586
77	H	H	H	H	H	OH	H	OH	H	H	H	OH	H	H	3	87.751	34.080	84.355	-22.744	1.266	3.548
78	H	H	OH	H	H	H	H	H	H	H	H	OH	H	H	2	80.484	38.050	81.057	-12.540	1.222	3.393
79	H	H	H	H	H	OH	OH	H	H	H	H	H	H	H	2	86.402	33.495	82.421	-15.692	1.210	3.354
80	H	H	H	H	OH	OH	H	H	H	H	OH	H	H	H	3	84.125	36.495	83.144	-15.054	1.178	3.249
81	H	H	OH	H	H	H	OH	H	H	H	H	H	H	H	2	81.511	37.894	81.928	-12.924	1.169	3.220
82	H	H	H	H	OH	H	OH	H	H	H	H	H	H	H	2	96.432	33.747	92.703	-12.982	1.168	3.217
83	H	H	H	H	H	H	OH	OH	H	H	H	H	H	H	2	84.518	32.200	79.243	-14.804	1.131	3.098
84	H	H	H	H	H	H	OH	H	H	OH	H	OH	H	H	3	90.495	35.349	88.368	-21.989	1.104	3.016
85	H	H	OH	H	OH	H	H	OH	H	H	H	H	H	H	3	78.670	37.046	78.239	-11.573	1.095	2.989
86	H	H	H	H	H	H	H	OH	H	OH	H	H	H	H	2	88.676	33.799	84.998	-15.445	1.094	2.986
87	H	H	H	H	H	H	OH	H	H	OH	H	H	H	H	2	88.243	36.744	87.510	-16.767	1.090	2.976
88	H	H	OH	H	H	OH	H	H	H	H	H	OH	H	H	3	81.349	36.778	80.651	-12.777	1.064	2.898
89	H	H	H	H	H	H	OH	H	H	H	H	H	H	H	1	94.781	34.135	91.440	-13.838	1.043	2.837
90	H	H	H	H	H	H	H	H	H	OH	H	H	H	OH	2	87.988	35.149	85.661	-16.085	0.929	2.531
91	H	H	H	H	OH	H	H	H	H	OH	H	OH	H	H	3	90.130	36.181	88.835	-16.457	0.915	2.497
92	H	H	OH	H	H	H	OH	OH	H	H	H	H	H	H	3	83.899	32.343	78.766	-13.698	0.890	2.435
93	H	H	OH	H	H	H	H	H	H	H	H	H	OH	H	2	82.899	37.260	82.683	-12.977	0.869	2.386
94	H	H	H	H	OH	OH	OH	H	H	H	H	H	H	H	3	81.332	37.052	80.908	-12.087	0.859	2.361
95	H	H	H	H	H	H	H	H	H	OH	OH	H	H	H	2	85.128	33.115	80.767	-13.871	0.754	2.125
96	H	H	OH	H	OH	H	OH	H	H	H	H	H	H	H	3	82.513	36.478	81.515	-12.572	0.754	2.125
97	H	H	H	H	OH	H	H	H	H	OH	OH	H	H	H	3	85.434	32.874	80.832	-13.430	0.749	2.114

*QM parameters (ETE(1), PA(1), BDEmin(1), and HE) are expressed in kcal mol^{-1}. The index one refers to the first oxidation step.
**All the abbreviations explained in the main text.

Table S1. (cont.)

#	R1	R2	R3	R4	R5	R6	R7	R8	R1'	R2'	R3'	R4'	R5'	R6'	*n*(OH)	ETE(1)	PA(1)	BDE_{min}(1)	HE	ln(ORAC)	ORAC
98	H	H	H	H	OH	H	H	H	H	OH	H	H	H	OH	3	88.379	35.349	86.251	-15.618	0.639	1.895
99	H	H	H	H	OH	H	H	H	H	H	H	H	H	H	1	83.029	36.288	81.841	-7.764	0.633	1.884
100	H	H	OH	H	OH	H	H	H	H	OH	H	H	H	H	3	88.592	36.584	87.700	-15.873	0.631	1.880
101	H	H	OH	H	H	OH	H	H	H	H	H	H	OH	H	3	83.693	35.979	82.196	-13.036	0.596	1.814
102	H	H	OH	H	OH	OH	H	H	H	H	H	OH	H	H	4	79.917	38.405	80.846	-10.250	0.491	1.634
103	H	H	OH	H	OH	OH	H	H	H	H	H	H	H	H	3	79.694	38.341	80.559	-10.013	0.451	1.570
104	H	H	H	H	H	OH	H	H	H	H	H	H	H	H	1	86.589	37.717	86.830	-14.319	0.447	1.564
105	H	H	H	H	OH	OH	H	H	H	OH	H	H	H	H	3	84.863	36.042	83.429	-13.042	0.367	1.444
106	H	H	H	H	H	H	H	H	H	H	H	H	OH	H	1	82.546	37.345	82.415	-7.713	0.362	1.436
107	H	H	OH	H	H	H	H	OH	H	H	H	H	H	H	2	83.105	36.984	82.613	-11.073	0.357	1.429
108	H	H	H	H	H	H	H	H	H	H	OH	H	H	H	1	87.243	38.215	87.982	-13.668	0.075	1.078
109	H	H	H	H	H	H	H	OH	H	H	H	H	H	H	1	87.713	35.091	85.328	-12.124	-0.032	0.969
110	H	H	H	H	H	H	H	H	H	H	H	OH	H	H	1	89.163	35.855	87.542	-14.168	-0.129	0.879
111	H	H	H	H	OH	H	H	H	H	H	OH	H	H	H	2	87.482	38.131	88.137	-13.228	-0.142	0.868
112	H	H	H	H	OH	H	H	H	H	H	H	OH	H	H	2	89.832	35.557	87.913	-13.711	-0.213	0.808
113	H	H	H	H	OH	H	H	H	H	OH	H	H	H	H	2	88.813	36.373	87.710	-11.199	-0.459	0.632
114	H	H	H	H	H	H	H	H	H	OH	H	H	H	H	1	88.469	36.738	87.731	-11.707	-0.499	0.607
115	H	H	H	H	H	H	H	H	H	H	H	H	H	OH	1	89.792	40.096	92.412	-8.368	-1.233	0.291

*QM parameters (ETE(1), PA(1), BDEmin(1), and HE) are expressed in kcal mol^{-1}. The index one refers to the first oxidation step.
**All the abbreviations explained in the main text.

Table S2. Cartesian coordinates of optimized geometries (ωB97XD/6-311+G**) for the reactions of genistein/quercetin (ROH) with peroxyl radical derived from AAPH ($PO^•$) as depicted in **Table 3** of the main text. Total energy (E) in Hartrees. N_i is the number of imaginary frequencies.

$PO^•$ (gas phase) E = -417.655794, N_i = 0				$PO^•$ (aqueous phase) E = -417.672069, N_i = 0			
C	-0.290267	0.724586	-0.860905	C	-0.260301	0.702239	-0.923418
H	-1.244617	1.854299	-2.059450	H	-0.949697	1.793777	-2.305185
N	-1.234037	0.902399	-1.697201	N	-0.969667	0.825576	-1.984143
N	0.613761	1.656958	-0.415208	N	0.406689	1.702721	-0.286959
H	0.682332	2.522230	-0.925077	H	0.519736	2.584267	-0.764545
H	1.462281	1.330873	0.018356	H	1.085955	1.483357	0.424518
C	-0.218998	-0.637345	-0.165952	C	-0.212699	-0.652785	-0.217193
C	-0.550541	-1.781076	-1.105300	C	-0.646934	-1.810885	-1.090354
H	0.126723	-1.785657	-1.961150	H	-0.079633	-1.839877	-2.021475
H	-1.566596	-1.651842	-1.475576	H	-1.706287	-1.711456	-1.326213
H	-0.467365	-2.728207	-0.570033	H	-0.500450	-2.744723	-0.544599
C	-1.087019	-0.622674	1.085921	C	-0.972020	-0.595841	1.100369
H	-0.803229	0.198768	1.747089	H	-0.548162	0.152717	1.771917
H	-0.983092	-1.568330	1.619750	H	-0.956479	-1.570879	1.588535
H	-2.127424	-0.489387	0.786305	H	-2.009766	-0.329691	0.888982
O	1.199181	-0.766692	0.247001	O	1.244499	-0.825013	0.069197
O	1.446291	-1.844735	0.924175	O	1.512600	-1.849334	0.813312

$PO^–$ (gas phase) E = -417.705167, N_i = 0				$PO^–$ (aqueous phase) E = -417.833891, N_i = 0			
C	0.116371	0.629058	-0.948064	C	0.127969	0.640560	-0.912234
H	1.753047	0.200819	-1.686004	H	1.931491	0.369321	-1.339764
N	1.099243	0.984738	-1.674135	N	1.217054	1.087487	-1.424084
N	-0.969818	1.491340	-0.795224	N	-1.006158	1.400157	-0.943840
H	-0.742798	2.439487	-1.057330	H	-0.884692	2.379816	-1.152723
H	-1.451801	1.419722	0.087038	H	-1.768285	1.175998	-0.324425
C	-0.014067	-0.725391	-0.244973	C	-0.018802	-0.740808	-0.254506
C	-1.172286	-1.521593	-0.869495	C	-1.239198	-1.481887	-0.805950
H	-1.032937	-1.584541	-1.952354	H	-1.196893	-1.535448	-1.897037
H	-2.146619	-1.066667	-0.663122	H	-2.172001	-0.994012	-0.515618
H	-1.113803	-2.528333	-0.450604	H	-1.254925	-2.496135	-0.403817
C	-0.238173	-0.534430	1.267343	C	-0.117691	-0.552026	1.263780
H	0.466652	0.199741	1.670553	H	0.777205	-0.052049	1.644006
H	-0.020522	-1.511773	1.707137	H	-0.216574	-1.521243	1.754414
H	-1.262840	-0.233838	1.519707	H	-0.990662	0.050410	1.525797
O	1.201711	-1.381430	-0.458084	O	1.180127	-1.423426	-0.609116
O	1.171954	-2.699095	0.166696	O	1.275347	-2.728900	0.014204

Table S2. (cont.)

POH (gas phase) E = -418.293640, N_i = 0				POH (aqueous phase) E = -418.317993, N_i = 0			
C	0.148481	0.680255	-0.837826	C	0.154720	0.666881	-0.868560
H	2.013180	0.571843	-1.078539	H	1.775587	0.247098	-1.740736
N	1.234843	1.205544	-1.234523	N	1.099412	1.000975	-1.669400
N	-1.021367	1.414725	-0.866313	N	-0.848875	1.543265	-0.583044
H	-0.931714	2.281797	-1.374688	H	-0.931822	2.344188	-1.191212
H	-1.886118	0.925827	-1.025298	H	-1.708576	1.203850	-0.181935
C	0.010302	-0.707901	-0.191422	C	0.044670	-0.688117	-0.155750
C	-1.196146	-1.487092	-0.714515	C	-1.023424	-1.537757	-0.841472
H	-1.210515	-1.504087	-1.807045	H	-0.779239	-1.681358	-1.896840
H	-2.134544	-1.064306	-0.347633	H	-1.997123	-1.049433	-0.770420
H	-1.146053	-2.512384	-0.343572	H	-1.101851	-2.511372	-0.354383
C	-0.028256	-0.551084	1.328852	C	-0.209671	-0.530313	1.339593
H	0.887160	-0.069549	1.677135	H	0.532976	0.131871	1.790139
H	-0.121348	-1.526509	1.808201	H	-0.155911	-1.508152	1.820936
H	-0.878770	0.070530	1.614469	H	-1.204921	-0.123995	1.527680
O	1.220352	-1.366295	-0.600605	O	1.356518	-1.266471	-0.360396
O	1.329788	-2.611312	0.080758	O	1.407405	-2.579512	0.168766
H	1.372408	-3.219201	-0.664242	H	1.251810	-3.130850	-0.609772

$POH^{•+}$ (gas phase) E = -417.978247, N_i = 0				$POH^{•+}$ (aqueous phase) E = -418.086010, N_i = 0			
C	0.032953	0.594468	-0.930532	C	0.050583	0.582294	-0.948990
H	1.720413	0.355702	-2.007566	H	1.952722	0.728783	-1.515100
N	0.742598	0.652598	-2.049360	N	0.995637	0.786420	-1.880902
N	-0.674059	1.616425	-0.532732	N	-0.839873	1.488641	-0.708836
H	-0.656788	2.493208	-1.039106	H	-0.800219	2.390471	-1.172006
H	-1.235582	1.561979	0.306042	H	-1.581210	1.326524	-0.036264
C	-0.065806	-0.762240	-0.215840	C	-0.038120	-0.785068	-0.266594
C	-1.163919	-1.574873	-0.909334	C	-1.236453	-1.534818	-0.840235
H	-0.938917	-1.712655	-1.967535	H	-1.137678	-1.652713	-1.920804
H	-2.131363	-1.078720	-0.806476	H	-2.157187	-0.992358	-0.618942
H	-1.225244	-2.551867	-0.430551	H	-1.296972	-2.515975	-0.369958
C	-0.289665	-0.620362	1.284033	C	-0.095178	-0.626064	1.247886
H	0.446772	0.039727	1.746366	H	0.736334	-0.017272	1.607244
H	-0.196137	-1.612587	1.727301	H	-0.038554	-1.618533	1.698524
H	-1.300853	-0.273807	1.513704	H	-1.039218	-0.176244	1.559781
O	1.239419	-1.275805	-0.507360	O	1.207273	-1.387482	-0.671724
O	1.269086	-2.640554	-0.133332	O	1.235026	-2.743727	-0.280943
H	2.088779	-2.679838	0.375470	H	1.744773	-2.722079	0.541058

Table S2. (cont.)

ROH (gas phase) E = -1104.12955, N_i = 0				ROH (aqueous phase) E = -1104.16782, N_i = 0			
H	0.137270	1.319117	-3.031008	H	0.155010	1.313485	-3.004989
C	-0.495780	0.447916	-2.939162	C	-0.478530	0.440032	-2.927245
C	-2.132770	-1.832971	-2.719430	C	-2.123276	-1.830134	-2.726425
C	-0.608845	-0.233774	-1.739077	C	-0.606976	-0.242034	-1.729043
C	-1.215990	-0.033157	-4.025443	C	-1.189587	-0.037489	-4.020212
C	-2.030805	-1.163055	-3.928076	C	-2.010379	-1.168039	-3.931831
C	-1.409576	-1.371136	-1.588879	C	-1.413414	-1.377069	-1.584453
H	-2.590821	-1.531970	-4.780006	H	-2.557020	-1.522468	-4.798061
O	0.105283	0.245928	-0.694341	O	0.100182	0.234039	-0.677104
C	0.056058	-0.362288	0.521990	C	0.037478	-0.359428	0.543800
C	-0.726213	-1.440255	0.766105	C	-0.753177	-1.437244	0.771634
C	-1.512908	-2.043033	-0.309963	C	-1.523001	-2.029885	-0.305100
O	-2.217589	-3.038725	-0.130867	O	-2.242143	-3.033167	-0.118016
C	0.894435	0.319522	1.522530	C	0.883188	0.322833	1.535789
C	2.468004	1.640153	3.419014	C	2.492507	1.646253	3.384915
C	1.717925	-0.399872	2.387890	C	1.731809	-0.399349	2.373588
C	0.875981	1.716835	1.602033	C	0.849306	1.719332	1.619911
C	1.653690	2.366680	2.538628	C	1.644664	2.376211	2.539657
C	2.496706	0.258433	3.332836	C	2.532117	0.264421	3.295083
H	1.785307	-1.478607	2.302340	H	1.788758	-1.478856	2.296747
H	0.249846	2.287170	0.923545	H	0.195911	2.296819	0.974845
H	3.146341	-0.290883	4.002971	H	3.201426	-0.285524	3.945980
O	-0.862292	-2.028310	1.975861	O	-0.914815	-2.041745	1.982621
H	-0.462106	-1.475312	2.653227	H	-0.530359	-1.508648	2.688072
O	-1.089664	0.646879	-5.187288	O	-1.053783	0.643226	-5.184093
H	-1.617258	0.228563	-5.870122	H	-1.594256	0.244462	-5.874901
O	1.702151	3.721436	2.698492	O	1.665937	3.732159	2.699015
H	1.146826	4.163483	2.054960	H	1.075837	4.156535	2.067612
O	3.225144	2.276422	4.336654	O	3.283308	2.278645	4.295339
H	3.089482	3.224975	4.246386	H	3.151459	3.231953	4.230211
O	-2.914398	-2.906317	-2.634832	O	-2.919103	-2.914259	-2.646065
H	-2.863434	-3.233845	-1.706968	H	-2.879078	-3.235066	-1.717282

Table S2. (cont.)

RO• (gas phase) E = -1103.508461, N_i = 0				RO• (aqueous phase) E = -1103.532714, N_i = 0			
H	-0.238901	1.634658	-2.731142	H	-0.228861	1.634860	-2.707473
C	-0.730969	0.672634	-2.712300	C	-0.725976	0.674145	-2.702280
C	-2.026270	-1.833176	-2.695640	C	-2.023969	-1.823360	-2.702316
C	-0.682866	-0.138180	-1.592973	C	-0.683615	-0.143921	-1.587651
C	-1.435877	0.208439	-3.817735	C	-1.428514	0.217845	-3.811848
C	-2.082338	-1.032534	-3.821355	C	-2.078268	-1.023172	-3.823256
C	-1.315239	-1.388823	-1.551021	C	-1.317167	-1.394137	-1.549032
H	-2.628448	-1.382341	-4.689926	H	-2.618922	-1.354001	-4.702336
O	0.003194	0.321783	-0.527229	O	0.002322	0.313463	-0.519005
C	0.126848	-0.380230	0.637411	C	0.123275	-0.391689	0.643092
C	-0.465790	-1.607007	0.744523	C	-0.471039	-1.619434	0.747418
C	-1.225416	-2.175231	-0.362471	C	-1.229536	-2.182841	-0.362853
O	-1.742819	-3.295823	-0.207557	O	-1.753045	-3.306941	-0.218142
C	0.912887	0.331450	1.629627	C	0.908553	0.318664	1.635137
C	2.453587	1.732745	3.566588	C	2.451202	1.732197	3.561573
C	1.162842	-0.232008	2.923706	C	1.163318	-0.237333	2.931416
C	1.433846	1.595560	1.305368	C	1.428965	1.582656	1.309739
C	2.179653	2.281150	2.233992	C	2.175475	2.274563	2.232209
C	1.898750	0.433992	3.853005	C	1.899974	0.435923	3.853499
H	0.757178	-1.203629	3.160438	H	0.764561	-1.207535	3.182837
H	1.256701	2.037584	0.335341	H	1.252473	2.026066	0.339567
H	2.090328	0.012359	4.832176	H	2.091899	0.014483	4.833242
O	-0.411093	-2.382605	1.835937	O	-0.408492	-2.386635	1.847156
H	-0.921465	-3.178622	1.605447	H	-0.911814	-3.195014	1.648795
O	-1.466864	1.018222	-4.894978	O	-1.458551	1.031397	-4.891501
H	-1.973683	0.613140	-5.601387	H	-1.966823	0.629028	-5.604734
O	2.695977	3.477356	1.982819	O	2.680230	3.477975	1.944547
O	3.134334	2.412252	4.347639	O	3.135745	2.404692	4.360360
H	3.169187	3.732333	2.795087	H	3.168545	3.787709	2.724973
O	-2.641632	-3.014395	-2.699156	O	-2.648538	-3.015778	-2.713898
H	-2.497819	-3.439533	-1.829515	H	-2.505581	-3.442352	-1.844516

Table S2. (cont.)

ROH$^{•+}$ (gas phase) E = -1103.871343, N_i = 0				ROH$^{•+}$ (aqueous phase) E=-1103.964126, N_i = 0			
H	-0.366125	1.640515	-2.746438	H	-0.224834	1.581521	-2.778673
C	-0.819995	0.660105	-2.718148	C	-0.723635	0.622170	-2.753599
C	-2.030397	-1.889926	-2.716251	C	-2.035203	-1.868540	-2.711205
C	-0.741385	-0.143308	-1.614585	C	-0.697856	-0.164036	-1.626260
C	-1.519143	0.170645	-3.837201	C	-1.421265	0.140535	-3.864731
C	-2.118705	-1.087146	-3.842348	C	-2.074230	-1.094797	-3.853358
C	-1.329508	-1.422361	-1.568084	C	-1.337248	-1.413427	-1.560709
H	-2.655097	-1.455990	-4.708496	H	-2.608314	-1.445263	-4.728513
O	-0.057237	0.351416	-0.537495	O	-0.011064	0.326201	-0.559119
C	0.106568	-0.318017	0.617757	C	0.099258	-0.323470	0.617641
C	-0.455972	-1.596004	0.724410	C	-0.543187	-1.556384	0.763398
C	-1.210998	-2.203481	-0.394229	C	-1.285105	-2.164786	-0.355471
O	-1.666620	-3.335046	-0.197970	O	-1.813425	-3.270985	-0.163391
C	0.852238	0.386685	1.603201	C	0.859466	0.376579	1.593783
C	2.323323	1.815099	3.503859	C	2.385466	1.778968	3.464611
C	1.124810	-0.166964	2.887324	C	1.286183	-0.245090	2.805790
C	1.340087	1.684949	1.294422	C	1.230231	1.720077	1.337886
C	2.058227	2.383692	2.220640	C	1.964816	2.414887	2.255836
C	1.845813	0.536730	3.814763	C	2.038590	0.445054	3.714633
H	0.763660	-1.150921	3.141933	H	1.039227	-1.275613	3.005828
H	1.139498	2.116239	0.322253	H	0.917740	2.216172	0.428416
H	2.060064	0.128168	4.793801	H	2.380427	-0.018738	4.630873
O	-0.370815	-2.350455	1.787926	O	-0.545656	-2.231570	1.886804
H	-0.855690	-3.176886	1.566554	H	-1.058399	-3.050197	1.739345
O	-1.567339	0.990885	-4.886998	O	-1.433998	0.931471	-4.953706
H	-2.056540	0.602177	-5.617360	H	-1.940580	0.524621	-5.666336
O	2.579436	3.614796	2.068348	O	2.349849	3.697888	2.137506
H	2.399542	3.994301	1.204779	H	2.039047	4.082780	1.309700
O	3.014253	2.486436	4.396039	O	3.101670	2.438700	4.354217
H	3.277898	3.349136	4.045714	H	3.266626	3.347045	4.058967
O	-2.602159	-3.081713	-2.729911	O	-2.663355	-3.053136	-2.702971
H	-2.461692	-3.533759	-1.878211	H	-2.541242	-3.468636	-1.827193

Table S2. (cont.)

RO (gas phase) E = -1103.613959, N_i = 0				RO (aqueous phase) E = -1103.703205, N_i = 0			
H	-0.238810	1.635037	-2.731468	H	-0.229977	1.631432	-2.706227
C	-0.733593	0.673950	-2.720236	C	-0.728755	0.671384	-2.705728
C	-2.022628	-1.828869	-2.692284	C	-2.026361	-1.823291	-2.708078
C	-0.686152	-0.143320	-1.594461	C	-0.690013	-0.152922	-1.591883
C	-1.435527	0.211766	-3.821068	C	-1.429365	0.219467	-3.816100
C	-2.081273	-1.028809	-3.823447	C	-2.079551	-1.020796	-3.829343
C	-1.314954	-1.385139	-1.554901	C	-1.322248	-1.398316	-1.555966
H	-2.628211	-1.380122	-4.692120	H	-2.620007	-1.350671	-4.709112
O	-0.002979	0.311841	-0.529657	O	-0.005130	0.297959	-0.517724
C	0.127463	-0.384785	0.644517	C	0.113225	-0.409389	0.641067
C	-0.485787	-1.628538	0.723558	C	-0.487239	-1.636418	0.730029
C	-1.220855	-2.176199	-0.350366	C	-1.232811	-2.189921	-0.361712
O	-1.767054	-3.314238	-0.242451	O	-1.770488	-3.321910	-0.240821
C	0.898955	0.313359	1.618827	C	0.899982	0.299332	1.638633
C	2.457253	1.743863	3.561521	C	2.456805	1.747114	3.556316
C	1.158248	-0.223377	2.901967	C	1.161808	-0.226175	2.913510
C	1.440541	1.602202	1.313180	C	1.431676	1.575019	1.326031
C	2.177005	2.273902	2.236727	C	2.174738	2.259224	2.249773
C	1.904753	0.459394	3.835730	C	1.916252	0.476123	3.841957
H	0.759980	-1.194925	3.156175	H	0.774883	-1.195075	3.191181
H	1.269583	2.054715	0.344979	H	1.259509	2.026205	0.356888
H	2.090883	0.028139	4.813762	H	2.102779	0.044865	4.820622
O	-0.421558	-2.406987	1.841266	O	-0.416514	-2.406919	1.853605
H	-0.932241	-3.195332	1.600586	H	-0.919008	-3.212265	1.653653
O	-1.474722	1.021298	-4.917597	O	-1.458955	1.036412	-4.898996
H	-1.986093	0.591362	-5.604064	H	-1.967567	0.631847	-5.610275
O	2.716969	3.498282	2.007091	O	2.696906	3.497543	1.960758
O	3.150288	2.447888	4.341484	O	3.167581	2.462027	4.365079
H	3.167733	3.681647	2.854532	H	3.169894	3.754251	2.770192
O	-2.635700	-3.017813	-2.680733	O	-2.652346	-3.018598	-2.719323
H	-2.463693	-3.408666	-1.786330	H	-2.501878	-3.436014	-1.843287

Table S2. (cont.)

POH (gas phase) E = -418.293640, N_i = 0				POH (aqueous phase) E = -418.317993, N_i = 0			
C	0.148481	0.680255	-0.837826	C	0.154720	0.666881	-0.868560
H	2.013180	0.571843	-1.078539	H	1.775587	0.247098	-1.740736
N	1.234843	1.205544	-1.234523	N	1.099412	1.000975	-1.669400
N	-1.021367	1.414725	-0.866313	N	-0.848875	1.543265	-0.583044
H	-0.931714	2.281797	-1.374688	H	-0.931822	2.344188	-1.191212
H	-1.886118	0.925827	-1.025298	H	-1.708576	1.203850	-0.181935
C	0.010302	-0.707901	-0.191422	C	0.044670	-0.688117	-0.155750
C	-1.196146	-1.487092	-0.714515	C	-1.023424	-1.537757	-0.841472
H	-1.210515	-1.504087	-1.807045	H	-0.779239	-1.681358	-1.896840
H	-2.134544	-1.064306	-0.347633	H	-1.997123	-1.049433	-0.770420
H	-1.146053	-2.512384	-0.343572	H	-1.101851	-2.511372	-0.354383
C	-0.028256	-0.551084	1.328852	C	-0.209671	-0.530313	1.339593
H	0.887160	-0.069549	1.677135	H	0.532976	0.131871	1.790139
H	-0.121348	-1.526509	1.808201	H	-0.155911	-1.508152	1.820936
H	-0.878770	0.070530	1.614469	H	-1.204921	-0.123995	1.527680
O	1.220352	-1.366295	-0.600605	O	1.356518	-1.266471	-0.360396
O	1.329788	-2.611312	0.080758	O	1.407405	-2.579512	0.168766
H	1.372408	-3.219201	-0.664242	H	1.251810	-3.130850	-0.609772

$POH^{\bullet+}$ (gas phase) E = -417.978247, N_i = 0				$POH^{\bullet+}$ (aqueous phase) E = -418.086010, N_i = 0			
C	0.032953	0.594468	-0.930532	C	0.050583	0.582294	-0.948990
H	1.720413	0.355702	-2.007566	H	1.952722	0.728783	-1.515100
N	0.742598	0.652598	-2.049360	N	0.995637	0.786420	-1.880902
N	-0.674059	1.616425	-0.532732	N	-0.839873	1.488641	-0.708836
H	-0.656788	2.493208	-1.039106	H	-0.800219	2.390471	-1.172006
H	-1.235582	1.561979	0.306042	H	-1.581210	1.326524	-0.036264
C	-0.065806	-0.762240	-0.215840	C	-0.038120	-0.785068	-0.266594
C	-1.163919	-1.574873	-0.909334	C	-1.236453	-1.534818	-0.840235
H	-0.938917	-1.712655	-1.967535	H	-1.137678	-1.652713	-1.920804
H	-2.131363	-1.078720	-0.806476	H	-2.157187	-0.992358	-0.618942
H	-1.225244	-2.551867	-0.430551	H	-1.296972	-2.515975	-0.369958
C	-0.289665	-0.620362	1.284033	C	-0.095178	-0.626064	1.247886
H	0.446772	0.039727	1.746366	H	0.736334	-0.017272	1.607244
H	-0.196137	-1.612587	1.727301	H	-0.038554	-1.618533	1.698524
H	-1.300853	-0.273807	1.513704	H	-1.039218	-0.176244	1.559781
O	1.239419	-1.275805	-0.507360	O	1.207273	-1.387482	-0.671724
O	1.269086	-2.640554	-0.133332	O	1.235026	-2.743727	-0.280943
H	2.088779	-2.679838	0.375470	H	1.744773	-2.722079	0.541058

Table S2. (cont.)

ROH [quercetin] (gas phase) E = -1104.141590, N_i = 0				ROH [quercetin] (aqueous phase) E = -1104.171173, N_i = 0			
C	0.677212	-0.215461	0.145641	C	0.645258	-0.211182	0.159416
O	-0.599041	0.181579	-0.127662	O	-0.643081	0.209765	0.048163
C	1.399374	2.117553	-0.008199	C	1.420344	2.071922	-0.218915
C	-0.932536	1.470777	-0.342088	C	-0.959150	1.500382	-0.202889
C	1.670156	0.709986	0.209404	C	1.669737	0.670026	0.027823
C	0.040901	2.474623	-0.291288	C	0.046373	2.466137	-0.331247
C	-2.263177	1.746459	-0.610356	C	-2.305077	1.805426	-0.319515
C	-2.616125	3.071794	-0.832569	C	-2.646581	3.126809	-0.576054
H	-3.004546	0.960963	-0.646533	H	-3.060758	1.037904	-0.218909
C	-1.677253	4.107877	-0.791739	C	-1.679415	4.131580	-0.708698
H	-1.965019	5.138312	-0.966863	H	-1.971797	5.155973	-0.908384
C	-0.351722	3.816929	-0.522557	C	-0.345039	3.803954	-0.590302
O	2.340797	2.930458	0.060031	O	2.385713	2.860538	-0.339837
C	0.774441	-1.665744	0.337780	C	0.739831	-1.655827	0.395679
C	0.890414	-4.445256	0.693556	C	0.863212	-4.412061	0.828433
C	-0.385853	-2.449062	0.227329	C	-0.285827	-2.490832	-0.069115
C	1.986163	-2.300708	0.628454	C	1.816454	-2.216877	1.085748
C	2.037005	-3.676630	0.803419	C	1.872367	-3.587379	1.299229
C	-0.325153	-3.814683	0.402769	C	-0.224621	-3.854247	0.145158
H	-1.336204	-1.979648	0.002877	H	-1.134491	-2.082662	-0.605890
H	2.892834	-1.722696	0.718925	H	2.606983	-1.590025	1.473113
H	2.973658	-4.171663	1.028593	H	2.701638	-4.028949	1.839349
O	0.947800	-5.782919	0.865008	O	0.939957	-5.754893	1.041477
H	0.063455	-6.145503	0.752194	H	0.174171	-6.185823	0.643552
O	-1.402483	-4.653376	0.313723	O	-1.179083	-4.736429	-0.278525
H	-2.200032	-4.162740	0.112271	H	-1.902443	-4.268923	-0.708465
O	-3.919364	3.315362	-1.092109	O	-3.967076	3.405439	-0.694924
H	-4.058350	4.254196	-1.229996	H	-4.099015	4.339435	-0.891254
O	0.543215	4.803103	-0.483494	O	0.585063	4.770403	-0.724783
H	1.418562	4.409955	-0.281450	H	1.465988	4.351678	-0.622742
O	2.958319	0.411826	0.472885	O	2.970997	0.286410	0.093846
H	3.422551	1.264337	0.452045	H	3.499367	1.082328	-0.070538

Table S2. (cont.)

RO• [quercetin] (gas phase) E = -1103.508461, N_i = 0				RO• [quercetin] (aqueous phase) E = -1103.532714, N_i = 0			
H	-0.238901	1.634658	-2.731142	H	-0.228861	1.634860	-2.707473
C	-0.730969	0.672634	-2.712300	C	-0.725976	0.674145	-2.702280
C	-2.026270	-1.833176	-2.695640	C	-2.023969	-1.823360	-2.702316
C	-0.682866	-0.138180	-1.592973	C	-0.683615	-0.143921	-1.587651
C	-1.435877	0.208439	-3.817735	C	-1.428514	0.217845	-3.811848
C	-2.082338	-1.032534	-3.821355	C	-2.078268	-1.023172	-3.823256
C	-1.315239	-1.388823	-1.551021	C	-1.317167	-1.394137	-1.549032
H	-2.628448	-1.382341	-4.689926	H	-2.618922	-1.354001	-4.702336
O	0.003194	0.321783	-0.527229	O	0.002322	0.313463	-0.519005
C	0.126848	-0.380230	0.637411	C	0.123275	-0.391689	0.643092
C	-0.465790	-1.607007	0.744523	C	-0.471039	-1.619434	0.747418
C	-1.225416	-2.175231	-0.362471	C	-1.229536	-2.182841	-0.362853
O	-1.742819	-3.295823	-0.207557	O	-1.753045	-3.306941	-0.218142
C	0.912887	0.331450	1.629627	C	0.908553	0.318664	1.635137
C	2.453587	1.732745	3.566588	C	2.451202	1.732197	3.561573
C	1.162842	-0.232008	2.923706	C	1.163318	-0.237333	2.931416
C	1.433846	1.595560	1.305368	C	1.428965	1.582656	1.309739
C	2.179653	2.281150	2.233992	C	2.175475	2.274563	2.232209
C	1.898750	0.433992	3.853005	C	1.899974	0.435923	3.853499
H	0.757178	-1.203629	3.160438	H	0.764561	-1.207535	3.182837
H	1.256701	2.037584	0.335341	H	1.252473	2.026066	0.339567
H	2.090328	0.012359	4.832176	H	2.091899	0.014483	4.833242
O	-0.411093	-2.382605	1.835937	O	-0.408492	-2.386635	1.847156
H	-0.921465	-3.178622	1.605447	H	-0.911814	-3.195014	1.648795
O	-1.466864	1.018222	-4.894978	O	-1.458551	1.031397	-4.891501
H	-1.973683	0.613140	-5.601387	H	-1.966823	0.629028	-5.604734
O	2.695977	3.477356	1.982819	O	2.680230	3.477975	1.944547
O	3.134334	2.412252	4.347639	O	3.135745	2.404692	4.360360
H	3.169187	3.732333	2.795087	H	3.168545	3.787709	2.724973
O	-2.641632	-3.014395	-2.699156	O	-2.648538	-3.015778	-2.713898
H	-2.497819	-3.439533	-1.829515	H	-2.505581	-3.442352	-1.844516

Table S2. (cont.)

ROH$^{•+}$ [quercetin] (gas phase) E = -1103.871343, N_i = 0				ROH$^{•+}$ [quercetin] (aqueous phase) E= -1103.964126, N_i = 0			
H	-0.366125	1.640515	-2.746438	H	-0.224834	1.581521	-2.778673
C	-0.819995	0.660105	-2.718148	C	-0.723635	0.622170	-2.753599
C	-2.030397	-1.889926	-2.716251	C	-2.035203	-1.868540	-2.711205
C	-0.741385	-0.143308	-1.614585	C	-0.697856	-0.164036	-1.626260
C	-1.519143	0.170645	-3.837201	C	-1.421265	0.140535	-3.864731
C	-2.118705	-1.087146	-3.842348	C	-2.074230	-1.094797	-3.853358
C	-1.329508	-1.422361	-1.568084	C	-1.337248	-1.413427	-1.560709
H	-2.655097	-1.455990	-4.708496	H	-2.608314	-1.445263	-4.728513
O	-0.057237	0.351416	-0.537495	O	-0.011064	0.326201	-0.559119
C	0.106568	-0.318017	0.617757	C	0.099258	-0.323470	0.617641
C	-0.455972	-1.596004	0.724410	C	-0.543187	-1.556384	0.763398
C	-1.210998	-2.203481	-0.394229	C	-1.285105	-2.164786	-0.355471
O	-1.666620	-3.335046	-0.197970	O	-1.813425	-3.270985	-0.163391
C	0.852238	0.386685	1.603201	C	0.859466	0.376579	1.593783
C	2.323323	1.815099	3.503859	C	2.385466	1.778968	3.464611
C	1.124810	-0.166964	2.887324	C	1.286183	-0.245090	2.805790
C	1.340087	1.684949	1.294422	C	1.230231	1.720077	1.337886
C	2.058227	2.383692	2.220640	C	1.964816	2.414887	2.255836
C	1.845813	0.536730	3.814763	C	2.038590	0.445054	3.714633
H	0.763660	-1.150921	3.141933	H	1.039227	-1.275613	3.005828
H	1.139498	2.116239	0.322253	H	0.917740	2.216172	0.428416
H	2.060064	0.128168	4.793801	H	2.380427	-0.018738	4.630873
O	-0.370815	-2.350455	1.787926	O	-0.545656	-2.231570	1.886804
H	-0.855690	-3.176886	1.566554	H	-1.058399	-3.050197	1.739345
O	-1.567339	0.990885	-4.886998	O	-1.433998	0.931471	-4.953706
H	-2.056540	0.602177	-5.617360	H	-1.940580	0.524621	-5.666336
O	2.579436	3.614796	2.068348	O	2.349849	3.697888	2.137506
H	2.399542	3.994301	1.204779	H	2.039047	4.082780	1.309700
O	3.014253	2.486436	4.396039	O	3.101670	2.438700	4.354217
H	3.277898	3.349136	4.045714	H	3.266626	3.347045	4.058967
O	-2.602159	-3.081713	-2.729911	O	-2.663355	-3.053136	-2.702971
H	-2.461692	-3.533759	-1.878211	H	-2.541242	-3.468636	-1.827193

Table S2. (cont.)

RO⁻ [quercetin] (gas phase) E = -1103.613959, N_i = 0				RO⁻ [quercetin] (aqueous phase) E = -1103.703205, N_i = 0			
H	-0.238810	1.635037	-2.731468	H	-0.229977	1.631432	-2.706227
C	-0.733593	0.673950	-2.720236	C	-0.728755	0.671384	-2.705728
C	-2.022628	-1.828869	-2.692284	C	-2.026361	-1.823291	-2.708078
C	-0.686152	-0.143320	-1.594461	C	-0.690013	-0.152922	-1.591883
C	-1.435527	0.211766	-3.821068	C	-1.429365	0.219467	-3.816100
C	-2.081273	-1.028809	-3.823447	C	-2.079551	-1.020796	-3.829343
C	-1.314954	-1.385139	-1.554901	C	-1.322248	-1.398316	-1.555966
H	-2.628211	-1.380122	-4.692120	H	-2.620007	-1.350671	-4.709112
O	-0.002979	0.311841	-0.529657	O	-0.005130	0.297959	-0.517724
C	0.127463	-0.384785	0.644517	C	0.113225	-0.409389	0.641067
C	-0.485787	-1.628538	0.723558	C	-0.487239	-1.636418	0.730029
C	-1.220855	-2.176199	-0.350366	C	-1.232811	-2.189921	-0.361712
O	-1.767054	-3.314238	-0.242451	O	-1.770488	-3.321910	-0.240821
C	0.898955	0.313359	1.618827	C	0.899982	0.299332	1.638633
C	2.457253	1.743863	3.561521	C	2.456805	1.747114	3.556316
C	1.158248	-0.223377	2.901967	C	1.161808	-0.226175	2.913510
C	1.440541	1.602202	1.313180	C	1.431676	1.575019	1.326031
C	2.177005	2.273902	2.236727	C	2.174738	2.259224	2.249773
C	1.904753	0.459394	3.835730	C	1.916252	0.476123	3.841957
H	0.759980	-1.194925	3.156175	H	0.774883	-1.195075	3.191181
H	1.269583	2.054715	0.344979	H	1.259509	2.026205	0.356888
H	2.090883	0.028139	4.813762	H	2.102779	0.044865	4.820622
O	-0.421558	-2.406987	1.841266	O	-0.416514	-2.406919	1.853605
H	-0.932241	-3.195332	1.600586	H	-0.919008	-3.212265	1.653653
O	-1.474722	1.021298	-4.917597	O	-1.458955	1.036412	-4.898996
H	-1.986093	0.591362	-5.604064	H	-1.967567	0.631847	-5.610275
O	2.716969	3.498282	2.007091	O	2.696906	3.497543	1.960758
O	3.150288	2.447888	4.341484	O	3.167581	2.462027	4.365079
H	3.167733	3.681647	2.854532	H	3.169894	3.754251	2.770192
O	-2.635700	-3.017813	-2.680733	O	-2.652346	-3.018598	-2.719323
H	-2.463693	-3.408666	-1.786330	H	-2.501878	-3.436014	-1.843287

Table S2. (cont.)

ROH [genistein] (gas phase) E = -953.683910, N_i = 0				ROH [genistein] (aqueous phase) E = -953.710691, N_i = 0			
H	0.692452	-4.428508	-0.968010	H	0.778934	-4.416083	-0.941836
C	0.761648	-3.484674	-1.490014	C	0.814266	-3.476835	-1.477470
C	0.949317	-1.029644	-2.856473	C	0.901167	-1.036531	-2.866864
C	0.481579	-2.290795	-0.852126	C	0.500759	-2.286571	-0.847999
C	1.136716	-3.428529	-2.827814	C	1.174367	-3.420888	-2.818723
C	1.232821	-2.217198	-3.513684	C	1.220603	-2.211950	-3.518843
C	0.561749	-1.047288	-1.491182	C	0.532607	-1.045375	-1.496989
H	1.526466	-2.179601	-4.556717	H	1.501739	-2.191332	-4.565527
C	0.251589	0.179641	-0.779991	C	0.184356	0.166356	-0.786173
C	-0.159704	0.008066	0.617192	C	-0.172465	-0.000666	0.616276
C	-0.174102	-1.239602	1.125332	C	-0.163097	-1.244334	1.137656
H	-0.428557	-1.457359	2.154774	H	-0.404992	-1.463848	2.169631
O	0.120330	-2.364924	0.456241	O	0.147780	-2.364249	0.465631
O	0.329096	1.287031	-1.324988	O	0.181108	1.277743	-1.353959
C	-0.559925	1.159387	1.455759	C	-0.564388	1.154118	1.457827
C	-1.299159	3.300922	3.102592	C	-1.287565	3.308086	3.088944
C	-1.700941	1.090972	2.259264	C	-1.741499	1.120129	2.208964
C	0.194662	2.333000	1.480081	C	0.242367	2.291756	1.537196
C	-0.167198	3.393609	2.296428	C	-0.111409	3.362916	2.344529
C	-2.069981	2.143652	3.081386	C	-2.103936	2.183727	3.023928
H	-2.326739	0.205102	2.226358	H	-2.390097	0.252463	2.150538
H	1.070825	2.421782	0.851004	H	1.165363	2.340285	0.971260
H	0.433769	4.298310	2.302990	H	0.523949	4.240733	2.402459
H	-2.958650	2.089311	3.698197	H	-3.019812	2.151902	3.602738
O	-1.701724	4.309350	3.920162	O	-1.684928	4.338198	3.897681
H	-1.106574	5.055667	3.833182	H	-1.045205	5.056254	3.849639
O	1.045276	0.117110	-3.523834	O	0.945957	0.120622	-3.553866
H	0.807777	0.839058	-2.896410	H	0.678266	0.834755	-2.930799
O	1.405151	-4.606506	-3.432530	O	1.478663	-4.594827	-3.422086
H	1.652030	-4.457342	-4.347167	H	1.717139	-4.446555	-4.343762

Table S2. (cont.)

RO$^{\bullet}$ [genistein] (gas phase) E = -953.0363578, N_i = 0				RO$^{\bullet}$ [genistein] (aqueous phase) E = -953.0633030, N_i = 0			
H	0.585629	-4.248582	-0.840286	H	0.611737	-4.239689	-0.829365
C	0.687278	-3.305023	-1.357388	C	0.706617	-3.297952	-1.353107
C	0.958283	-0.850547	-2.712903	C	0.942822	-0.849686	-2.712682
C	0.404335	-2.109257	-0.730181	C	0.401643	-2.104336	-0.731968
C	1.110146	-3.252585	-2.683014	C	1.136472	-3.242705	-2.675509
C	1.246797	-2.042057	-3.361676	C	1.257170	-2.030625	-3.358720
C	0.524825	-0.864647	-1.361220	C	0.506579	-0.858281	-1.363097
H	1.575627	-2.006636	-4.394131	H	1.589453	-2.010092	-4.390238
C	0.210873	0.364271	-0.658326	C	0.162292	0.357699	-0.659148
C	-0.247795	0.194810	0.728685	C	-0.268046	0.185388	0.727267
C	-0.307912	-1.059923	1.228704	C	-0.337709	-1.071033	1.230124
H	-0.597772	-1.279883	2.248342	H	-0.637141	-1.295918	2.245829
O	-0.006178	-2.180172	0.567885	O	-0.025322	-2.182619	0.564354
O	0.311294	1.470273	-1.198798	O	0.207621	1.470157	-1.215833
C	-0.629817	1.339069	1.569320	C	-0.629267	1.326385	1.576346
C	-1.320712	3.502189	3.289203	C	-1.304591	3.505750	3.264807
C	-1.725758	1.229585	2.460024	C	-1.730538	1.233870	2.464845
C	0.103114	2.550959	1.525942	C	0.131762	2.523893	1.542890
C	-0.216095	3.590922	2.351971	C	-0.181181	3.574774	2.354321
C	-2.068731	2.257650	3.291978	C	-2.066480	2.274801	3.280978
H	-2.320577	0.322050	2.457112	H	-2.327590	0.328983	2.476026
H	0.925848	2.642409	0.829933	H	0.984850	2.591275	0.880881
H	0.345774	4.517562	2.338364	H	0.406794	4.485522	2.346022
H	-2.913305	2.187746	3.967536	H	-2.916530	2.213375	3.950930
O	-1.616499	4.440086	4.045654	O	-1.604440	4.469392	4.007710
O	1.093770	0.292601	-3.377500	O	1.058604	0.307943	-3.388194
H	0.843695	1.021095	-2.765328	H	0.777986	1.028275	-2.780344
O	1.380854	-4.432929	-3.277067	O	1.430532	-4.420311	-3.273161
H	1.663012	-4.291037	-4.182835	H	1.715900	-4.274236	-4.181965

Table S2. (cont.)

ROH$^{\bullet+}$ [genistein] (gas phase) E = -953.4031426, N_i = 0				ROH$^{\bullet+}$ [genistein] (aqueous phase) E= -953.497316924, N_i = 0			
H	3.690503	-2.434336	-0.154428	H	3.685238	-2.440190	-0.046796
C	3.413415	-1.391807	-0.083323	C	3.415016	-1.393298	-0.016973
C	2.713189	1.334226	0.102008	C	2.706596	1.327185	0.044849
C	2.107271	-0.991828	-0.049949	C	2.099565	-0.996398	-0.024598
C	4.396475	-0.386649	-0.021999	C	4.385649	-0.390826	0.025442
C	4.052593	0.956104	0.069534	C	4.041207	0.959494	0.058256
C	1.692907	0.345485	0.044115	C	1.688021	0.341394	0.009684
H	4.808121	1.731801	0.114028	H	4.809413	1.723590	0.085290
C	0.298822	0.701141	0.071908	C	0.285878	0.686836	-0.016307
C	-0.655608	-0.441803	-0.023706	C	-0.650271	-0.450584	-0.043847
C	-0.113287	-1.710860	-0.104525	C	-0.122672	-1.717069	-0.089504
H	-0.710105	-2.614105	-0.130851	H	-0.721476	-2.618790	-0.102754
O	1.158356	-1.992012	-0.110922	O	1.160502	-2.000955	-0.075562
O	-0.085353	1.868664	0.144495	O	-0.096210	1.865678	-0.050488
C	-2.080366	-0.279923	-0.013177	C	-2.081494	-0.283839	-0.013162
C	-4.872871	0.015528	0.036802	C	-4.859445	0.028212	0.041906
C	-2.944286	-1.287530	-0.548826	C	-2.940760	-1.252076	-0.620089
C	-2.690979	0.890387	0.537903	C	-2.679081	0.841311	0.634814
C	-4.048817	1.029348	0.571741	C	-4.031241	0.992950	0.671153
C	-4.297551	-1.147470	-0.532872	C	-4.291185	-1.100611	-0.605402
H	-2.528791	-2.165119	-1.027428	H	-2.515139	-2.098490	-1.143014
H	-2.060584	1.668409	0.940966	H	-2.049999	1.563929	1.132188
H	-4.495736	1.914344	1.012045	H	-4.490417	1.829800	1.184264
H	-4.954553	-1.895019	-0.959088	H	-4.949550	-1.811853	-1.087689
O	-6.186662	0.085005	0.028303	O	-6.167698	0.122178	0.030965
H	-6.517203	0.900170	0.422319	H	-6.483624	0.915349	0.487895
O	2.426776	2.621008	0.187042	O	2.401450	2.634229	0.065976
H	1.454309	2.728967	0.191802	H	1.424017	2.712049	0.025293
O	5.661274	-0.811077	-0.057903	O	5.673792	-0.793265	0.029130
H	6.280818	-0.078040	-0.011448	H	6.265994	-0.032933	0.053644

Table S2. (cont.)

RO⁻ [genistein] (gas phase) E= -953.1446378, N_i = 0				RO⁻ [genistein] (aqueous phase) E = -953.2426674, N_i = 0			
H	4.576103	0.707523	-1.105729	H	4.560679	0.853637	-1.068290
C	3.636099	0.795901	-1.636460	C	3.628966	0.885783	-1.620594
C	1.190104	1.037555	-3.011363	C	1.198391	0.952542	-3.021115
C	2.459427	0.519408	-1.012172	C	2.448216	0.563925	-0.998354
C	3.652607	1.218405	-3.024417	C	3.627292	1.262889	-2.996400
C	2.355642	1.320530	-3.663841	C	2.361614	1.280053	-3.668528
C	1.185270	0.616310	-1.635275	C	1.193985	0.581697	-1.642995
H	2.324069	1.634264	-4.700897	H	2.328659	1.559626	-4.716075
C	-0.012845	0.309040	-0.941609	C	-0.003119	0.232413	-0.941975
C	0.158853	-0.132383	0.460513	C	0.158913	-0.121080	0.467480
C	1.403276	-0.167194	0.971082	C	1.398738	-0.107720	0.993382
H	1.612631	-0.437067	2.000164	H	1.614748	-0.348153	2.026706
O	2.526173	0.130907	0.313863	O	2.520581	0.207417	0.324421
O	-1.156761	0.392441	-1.451588	O	-1.127588	0.217211	-1.504073
C	-0.993640	-0.521153	1.301530	C	-0.998344	-0.510199	1.307581
C	-3.145914	-1.224372	2.965411	C	-3.156716	-1.224340	2.940075
C	-0.926124	-1.628895	2.151427	C	-0.973967	-1.690974	2.053457
C	-2.182579	0.213530	1.288249	C	-2.130417	0.304203	1.393586
C	-3.245621	-0.130709	2.110955	C	-3.203441	-0.044613	2.200938
C	-1.980839	-1.978206	2.984712	C	-2.039134	-2.048974	2.869166
H	-0.034009	-2.246536	2.146587	H	-0.111743	-2.346409	1.990516
H	-2.274827	1.053411	0.612855	H	-2.172377	1.230264	0.832017
H	-4.160576	0.456092	2.082864	H	-4.076169	0.597420	2.263320
H	-1.918236	-2.841958	3.636360	H	-2.013401	-2.967841	3.443656
O	-4.169099	-1.601879	3.794623	O	-4.189356	-1.616982	3.749461
H	-4.915151	-1.018675	3.650065	H	-4.901764	-0.970898	3.704406
O	0.008435	1.148386	-3.656896	O	0.025244	0.983983	-3.703841
H	-0.687185	0.898129	-3.008448	H	-0.676273	0.710918	-3.068112
O	4.714717	1.477198	-3.622567	O	4.707786	1.574201	-3.609817

Table S2. (cont.)

POH (gas phase) E = -418.293640, N_i = 0				POH (aqueous phase) E = -418.317993, N_i = 0			
C	0.148481	0.680255	-0.837826	C	0.154720	0.666881	-0.868560
H	2.013180	0.571843	-1.078539	H	1.775587	0.247098	-1.740736
N	1.234843	1.205544	-1.234523	N	1.099412	1.000975	-1.669400
N	-1.021367	1.414725	-0.866313	N	-0.848875	1.543265	-0.583044
H	-0.931714	2.281797	-1.374688	H	-0.931822	2.344188	-1.191212
H	-1.886118	0.925827	-1.025298	H	-1.708576	1.203850	-0.181935
C	0.010302	-0.707901	-0.191422	C	0.044670	-0.688117	-0.155750
C	-1.196146	-1.487092	-0.714515	C	-1.023424	-1.537757	-0.841472
H	-1.210515	-1.504087	-1.807045	H	-0.779239	-1.681358	-1.896840
H	-2.134544	-1.064306	-0.347633	H	-1.997123	-1.049433	-0.770420
H	-1.146053	-2.512384	-0.343572	H	-1.101851	-2.511372	-0.354383
C	-0.028256	-0.551084	1.328852	C	-0.209671	-0.530313	1.339593
H	0.887160	-0.069549	1.677135	H	0.532976	0.131871	1.790139
H	-0.121348	-1.526509	1.808201	H	-0.155911	-1.508152	1.820936
H	-0.878770	0.070530	1.614469	H	-1.204921	-0.123995	1.527680
O	1.220352	-1.366295	-0.600605	O	1.356518	-1.266471	-0.360396
O	1.329788	-2.611312	0.080758	O	1.407405	-2.579512	0.168766
H	1.372408	-3.219201	-0.664242	H	1.251810	-3.130850	-0.609772

$POH^{•+}$ (gas phase) E = -417.978247, N_i = 0				$POH^{•+}$ (aqueous phase) E = -418.086010, N_i = 0			
C	0.032953	0.594468	-0.930532	C	0.050583	0.582294	-0.948990
H	1.720413	0.355702	-2.007566	H	1.952722	0.728783	-1.515100
N	0.742598	0.652598	-2.049360	N	0.995637	0.786420	-1.880902
N	-0.674059	1.616425	-0.532732	N	-0.839873	1.488641	-0.708836
H	-0.656788	2.493208	-1.039106	H	-0.800219	2.390471	-1.172006
H	-1.235582	1.561979	0.306042	H	-1.581210	1.326524	-0.036264
C	-0.065806	-0.762240	-0.215840	C	-0.038120	-0.785068	-0.266594
C	-1.163919	-1.574873	-0.909334	C	-1.236453	-1.534818	-0.840235
H	-0.938917	-1.712655	-1.967535	H	-1.137678	-1.652713	-1.920804
H	-2.131363	-1.078720	-0.806476	H	-2.157187	-0.992358	-0.618942
H	-1.225244	-2.551867	-0.430551	H	-1.296972	-2.515975	-0.369958
C	-0.289665	-0.620362	1.284033	C	-0.095178	-0.626064	1.247886
H	0.446772	0.039727	1.746366	H	0.736334	-0.017272	1.607244
H	-0.196137	-1.612587	1.727301	H	-0.038554	-1.618533	1.698524
H	-1.300853	-0.273807	1.513704	H	-1.039218	-0.176244	1.559781
O	1.239419	-1.275805	-0.507360	O	1.207273	-1.387482	-0.671724
O	1.269086	-2.640554	-0.133332	O	1.235026	-2.743727	-0.280943
H	2.088779	-2.679838	0.375470	H	1.744773	-2.722079	0.541058

Table S2. (cont.)

ROH (gas phase) E = -1104.12955, N_i = 0				ROH (aqueous phase) E = -1104.16782, N_i = 0			
H	0.137270	1.319117	-3.031008	H	0.155010	1.313485	-3.004989
C	-0.495780	0.447916	-2.939162	C	-0.478530	0.440032	-2.927245
C	-2.132770	-1.832971	-2.719430	C	-2.123276	-1.830134	-2.726425
C	-0.608845	-0.233774	-1.739077	C	-0.606976	-0.242034	-1.729043
C	-1.215990	-0.033157	-4.025443	C	-1.189587	-0.037489	-4.020212
C	-2.030805	-1.163055	-3.928076	C	-2.010379	-1.168039	-3.931831
C	-1.409576	-1.371136	-1.588879	C	-1.413414	-1.377069	-1.584453
H	-2.590821	-1.531970	-4.780006	H	-2.557020	-1.522468	-4.798061
O	0.105283	0.245928	-0.694341	O	0.100182	0.234039	-0.677104
C	0.056058	-0.362288	0.521990	C	0.037478	-0.359428	0.543800
C	-0.726213	-1.440255	0.766105	C	-0.753177	-1.437244	0.771634
C	-1.512908	-2.043033	-0.309963	C	-1.523001	-2.029885	-0.305100
O	-2.217589	-3.038725	-0.130867	O	-2.242143	-3.033167	-0.118016
C	0.894435	0.319522	1.522530	C	0.883188	0.322833	1.535789
C	2.468004	1.640153	3.419014	C	2.492507	1.646253	3.384915
C	1.717925	-0.399872	2.387890	C	1.731809	-0.399349	2.373588
C	0.875981	1.716835	1.602033	C	0.849306	1.719332	1.619911
C	1.653690	2.366680	2.538628	C	1.644664	2.376211	2.539657
C	2.496706	0.258433	3.332836	C	2.532117	0.264421	3.295083
H	1.785307	-1.478607	2.302340	H	1.788758	-1.478856	2.296747
H	0.249846	2.287170	0.923545	H	0.195911	2.296819	0.974845
H	3.146341	-0.290883	4.002971	H	3.201426	-0.285524	3.945980
O	-0.862292	-2.028310	1.975861	O	-0.914815	-2.041745	1.982621
H	-0.462106	-1.475312	2.653227	H	-0.530359	-1.508648	2.688072
O	-1.089664	0.646879	-5.187288	O	-1.053783	0.643226	-5.184093
H	-1.617258	0.228563	-5.870122	H	-1.594256	0.244462	-5.874901
O	1.702151	3.721436	2.698492	O	1.665937	3.732159	2.699015
H	1.146826	4.163483	2.054960	H	1.075837	4.156535	2.067612
O	3.225144	2.276422	4.336654	O	3.283308	2.278645	4.295339
H	3.089482	3.224975	4.246386	H	3.151459	3.231953	4.230211
O	-2.914398	-2.906317	-2.634832	O	-2.919103	-2.914259	-2.646065
H	-2.863434	-3.233845	-1.706968	H	-2.879078	-3.235066	-1.717282

Table S2. (cont.)

RO$^{\bullet}$ (gas phase) E = -1103.508461, N_i = 0				RO$^{\bullet}$ (aqueous phase) E = -1103.532714, N_i = 0			
H	-0.238901	1.634658	-2.731142	H	-0.228861	1.634860	-2.707473
C	-0.730969	0.672634	-2.712300	C	-0.725976	0.674145	-2.702280
C	-2.026270	-1.833176	-2.695640	C	-2.023969	-1.823360	-2.702316
C	-0.682866	-0.138180	-1.592973	C	-0.683615	-0.143921	-1.587651
C	-1.435877	0.208439	-3.817735	C	-1.428514	0.217845	-3.811848
C	-2.082338	-1.032534	-3.821355	C	-2.078268	-1.023172	-3.823256
C	-1.315239	-1.388823	-1.551021	C	-1.317167	-1.394137	-1.549032
H	-2.628448	-1.382341	-4.689926	H	-2.618922	-1.354001	-4.702336
O	0.003194	0.321783	-0.527229	O	0.002322	0.313463	-0.519005
C	0.126848	-0.380230	0.637411	C	0.123275	-0.391689	0.643092
C	-0.465790	-1.607007	0.744523	C	-0.471039	-1.619434	0.747418
C	-1.225416	-2.175231	-0.362471	C	-1.229536	-2.182841	-0.362853
O	-1.742819	-3.295823	-0.207557	O	-1.753045	-3.306941	-0.218142
C	0.912887	0.331450	1.629627	C	0.908553	0.318664	1.635137
C	2.453587	1.732745	3.566588	C	2.451202	1.732197	3.561573
C	1.162842	-0.232008	2.923706	C	1.163318	-0.237333	2.931416
C	1.433846	1.595560	1.305368	C	1.428965	1.582656	1.309739
C	2.179653	2.281150	2.233992	C	2.175475	2.274563	2.232209
C	1.898750	0.433992	3.853005	C	1.899974	0.435923	3.853499
H	0.757178	-1.203629	3.160438	H	0.764561	-1.207535	3.182837
H	1.256701	2.037584	0.335341	H	1.252473	2.026066	0.339567
H	2.090328	0.012359	4.832176	H	2.091899	0.014483	4.833242
O	-0.411093	-2.382605	1.835937	O	-0.408492	-2.386635	1.847156
H	-0.921465	-3.178622	1.605447	H	-0.911814	-3.195014	1.648795
O	-1.466864	1.018222	-4.894978	O	-1.458551	1.031397	-4.891501
H	-1.973683	0.613140	-5.601387	H	-1.966823	0.629028	-5.604734
O	2.695977	3.477356	1.982819	O	2.680230	3.477975	1.944547
O	3.134334	2.412252	4.347639	O	3.135745	2.404692	4.360360
H	3.169187	3.732333	2.795087	H	3.168545	3.787709	2.724973
O	-2.641632	-3.014395	-2.699156	O	-2.648538	-3.015778	-2.713898
H	-2.497819	-3.439533	-1.829515	H	-2.505581	-3.442352	-1.844516

Table S2. (cont.)

ROH$^{\bullet+}$ (gas phase) E = -1103.871343, N_i = 0				ROH$^{\bullet+}$ (aqueous phase) E=-1103.964126, N_i = 0			
H	-0.366125	1.640515	-2.746438	H	-0.224834	1.581521	-2.778673
C	-0.819995	0.660105	-2.718148	C	-0.723635	0.622170	-2.753599
C	-2.030397	-1.889926	-2.716251	C	-2.035203	-1.868540	-2.711205
C	-0.741385	-0.143308	-1.614585	C	-0.697856	-0.164036	-1.626260
C	-1.519143	0.170645	-3.837201	C	-1.421265	0.140535	-3.864731
C	-2.118705	-1.087146	-3.842348	C	-2.074230	-1.094797	-3.853358
C	-1.329508	-1.422361	-1.568084	C	-1.337248	-1.413427	-1.560709
H	-2.655097	-1.455990	-4.708496	H	-2.608314	-1.445263	-4.728513
O	-0.057237	0.351416	-0.537495	O	-0.011064	0.326201	-0.559119
C	0.106568	-0.318017	0.617757	C	0.099258	-0.323470	0.617641
C	-0.455972	-1.596004	0.724410	C	-0.543187	-1.556384	0.763398
C	-1.210998	-2.203481	-0.394229	C	-1.285105	-2.164786	-0.355471
O	-1.666620	-3.335046	-0.197970	O	-1.813425	-3.270985	-0.163391
C	0.852238	0.386685	1.603201	C	0.859466	0.376579	1.593783
C	2.323323	1.815099	3.503859	C	2.385466	1.778968	3.464611
C	1.124810	-0.166964	2.887324	C	1.286183	-0.245090	2.805790
C	1.340087	1.684949	1.294422	C	1.230231	1.720077	1.337886
C	2.058227	2.383692	2.220640	C	1.964816	2.414887	2.255836
C	1.845813	0.536730	3.814763	C	2.038590	0.445054	3.714633
H	0.763660	-1.150921	3.141933	H	1.039227	-1.275613	3.005828
H	1.139498	2.116239	0.322253	H	0.917740	2.216172	0.428416
H	2.060064	0.128168	4.793801	H	2.380427	-0.018738	4.630873
O	-0.370815	-2.350455	1.787926	O	-0.545656	-2.231570	1.886804
H	-0.855690	-3.176886	1.566554	H	-1.058399	-3.050197	1.739345
O	-1.567339	0.990885	-4.886998	O	-1.433998	0.931471	-4.953706
H	-2.056540	0.602177	-5.617360	H	-1.940580	0.524621	-5.666336
O	2.579436	3.614796	2.068348	O	2.349849	3.697888	2.137506
H	2.399542	3.994301	1.204779	H	2.039047	4.082780	1.309700
O	3.014253	2.486436	4.396039	O	3.101670	2.438700	4.354217
H	3.277898	3.349136	4.045714	H	3.266626	3.347045	4.058967
O	-2.602159	-3.081713	-2.729911	O	-2.663355	-3.053136	-2.702971
H	-2.461692	-3.533759	-1.878211	H	-2.541242	-3.468636	-1.827193

Table S2. (cont.)

RO (gas phase) E = -1103.613959, N_i = 0				RO (aqueous phase) E = -1103.703205, N_i = 0			
H	-0.238810	1.635037	-2.731468	H	-0.229977	1.631432	-2.706227
C	-0.733593	0.673950	-2.720236	C	-0.728755	0.671384	-2.705728
C	-2.022628	-1.828869	-2.692284	C	-2.026361	-1.823291	-2.708078
C	-0.686152	-0.143320	-1.594461	C	-0.690013	-0.152922	-1.591883
C	-1.435527	0.211766	-3.821068	C	-1.429365	0.219467	-3.816100
C	-2.081273	-1.028809	-3.823447	C	-2.079551	-1.020796	-3.829343
C	-1.314954	-1.385139	-1.554901	C	-1.322248	-1.398316	-1.555966
H	-2.628211	-1.380122	-4.692120	H	-2.620007	-1.350671	-4.709112
O	-0.002979	0.311841	-0.529657	O	-0.005130	0.297959	-0.517724
C	0.127463	-0.384785	0.644517	C	0.113225	-0.409389	0.641067
C	-0.485787	-1.628538	0.723558	C	-0.487239	-1.636418	0.730029
C	-1.220855	-2.176199	-0.350366	C	-1.232811	-2.189921	-0.361712
O	-1.767054	-3.314238	-0.242451	O	-1.770488	-3.321910	-0.240821
C	0.898955	0.313359	1.618827	C	0.899982	0.299332	1.638633
C	2.457253	1.743863	3.561521	C	2.456805	1.747114	3.556316
C	1.158248	-0.223377	2.901967	C	1.161808	-0.226175	2.913510
C	1.440541	1.602202	1.313180	C	1.431676	1.575019	1.326031
C	2.177005	2.273902	2.236727	C	2.174738	2.259224	2.249773
C	1.904753	0.459394	3.835730	C	1.916252	0.476123	3.841957
H	0.759980	-1.194925	3.156175	H	0.774883	-1.195075	3.191181
H	1.269583	2.054715	0.344979	H	1.259509	2.026205	0.356888
H	2.090883	0.028139	4.813762	H	2.102779	0.044865	4.820622
O	-0.421558	-2.406987	1.841266	O	-0.416514	-2.406919	1.853605
H	-0.932241	-3.195332	1.600586	H	-0.919008	-3.212265	1.653653
O	-1.474722	1.021298	-4.917597	O	-1.458955	1.036412	-4.898996
H	-1.986093	0.591362	-5.604064	H	-1.967567	0.631847	-5.610275
O	2.716969	3.498282	2.007091	O	2.696906	3.497543	1.960758
O	3.150288	2.447888	4.341484	O	3.167581	2.462027	4.365079
H	3.167733	3.681647	2.854532	H	3.169894	3.754251	2.770192
O	-2.635700	-3.017813	-2.680733	O	-2.652346	-3.018598	-2.719323
H	-2.463693	-3.408666	-1.786330	H	-2.501878	-3.436014	-1.843287

Table S3. Cartesian coordinates of optimized geometries (ωB97XD/6-311+G**) of 36 flavonoids involved in the QSAR modelling. Total energy (E) in Hartrees. N_i is the number of imaginary frequencies.

Genistein				Naringenin			
N_i = 0; E = -953.710691				N_i = 0; E = -954.93386			
C	0.2866	0.6498	0.0806	C	-0.6931	0.4042	0.4493
O	-0.1046	1.8215	0.2585	C	-0.2129	1.6599	-0.2612
C	1.6954	0.3211	0.0353	C	1.273	1.8242	-0.1183
C	-0.6319	-0.4697	-0.0786	C	2.0415	0.6069	-0.001
C	2.1126	-1.0054	-0.1355	C	3.4586	0.6211	0.0196
C	2.7048	1.3079	0.1756	C	4.1863	-0.5569	0.0226
C	-0.1165	-1.705	-0.2427	C	3.4992	-1.7691	-0.0045
C	-2.1005	-0.2775	-0.0375	C	2.1057	-1.8311	-0.0181
O	1.1927	-2.0015	-0.269	C	1.3913	-0.6492	-0.01
C	3.4428	-1.3798	-0.1776	O	0.0427	-0.7466	-0.032
O	2.3746	2.6016	0.3498	O	1.7994	2.9455	-0.1438
C	4.0413	0.9603	0.1356	C	-2.1556	0.1207	0.2546
C	-2.8978	-1.0633	0.7979	C	-3.0141	0.0798	1.3504
C	-2.7231	0.6759	-0.8467	C	-4.3713	-0.1656	1.1874
C	4.3981	-0.3797	-0.0406	C	-4.8791	-0.3846	-0.0887
C	-4.2774	-0.9127	0.8231	C	-4.0327	-0.3547	-1.1964
C	-4.1006	0.8389	-0.8271	C	-2.6812	-0.0978	-1.019
O	5.697	-0.7615	-0.0837	O	-6.2206	-0.6281	-0.2036
C	-4.8787	0.0427	0.0103	O	4.1577	-2.9512	-0.0071
O	-6.2404	0.1653	0.0711	O	4.1305	1.7871	0.0212
H	-0.7198	-2.5936	-0.3769	H	-0.4797	0.4924	1.5198
H	3.7257	-2.4153	-0.3122	H	-0.4336	1.6001	-1.3338
H	1.391	2.6475	0.3625	H	-0.7193	2.5382	0.1414
H	4.8029	1.7241	0.243	H	5.2696	-0.5299	0.0367
H	-2.4333	-1.7994	1.4454	H	1.5945	-2.7849	-0.019
H	-2.1285	1.2927	-1.5104	H	-2.6194	0.2423	2.348
H	-4.8903	-1.5243	1.4752	H	-5.0381	-0.1944	2.0415
H	-4.5754	1.5797	-1.4623	H	-4.4372	-0.5286	-2.1883
H	6.275	0.0029	0.0163	H	-2.0305	-0.0741	-1.887
H	-6.5325	0.861	-0.527	H	-6.4524	-0.7674	-1.1276
				H	5.1095	-2.8038	0.0164
				H	3.466	2.5099	-0.0252

Table S3. (cont.)

Scutellarin				3,5,7,8,3',4'-Hexahydroxyflavone			
N_i = 0; E = -1028.94282				N_i = 0; E =-1179.08417			
C	0.4861	1.6956	-0.0903	C	0.1421	1.8662	0.0645
C	1.0268	0.4542	-0.0001	C	-0.5111	0.6765	-0.0304
C	-0.9341	1.8871	-0.0954	C	1.5909	1.9323	0.09
O	-1.4676	3.0195	-0.1683	O	2.1501	3.0519	0.1803
C	-1.7298	0.6786	-0.0257	C	2.2942	0.6838	0.0239
C	-3.1404	0.7108	-0.039	C	3.7086	0.599	0.06
C	-3.8626	-0.467	0.0213	C	4.3308	-0.635	0.0041
C	-3.1868	-1.6927	0.0982	C	3.5558	-1.7971	-0.0912
C	-1.8012	-1.7535	0.1145	C	2.1612	-1.7473	-0.1172
C	-1.0971	-0.5645	0.0515	C	1.5501	-0.5017	-0.053
O	0.2634	-0.6527	0.0741	O	0.2005	-0.4863	-0.0903
C	2.4618	0.1449	0.0123	C	-1.9577	0.4356	-0.0587
C	3.3998	1.1102	0.3959	C	-2.8661	1.4327	-0.4288
C	4.7529	0.8242	0.3911	C	-4.2304	1.1636	-0.455
C	5.1907	-0.4399	-0.0018	C	-4.7059	-0.0953	-0.1158
C	4.2695	-1.4147	-0.3804	C	-3.7983	-1.1031	0.2501
C	2.9161	-1.1214	-0.3686	C	-2.4408	-0.8413	0.2775
O	-3.8874	-2.8493	0.1594	O	1.4648	-2.9321	-0.1522
O	-5.229	-0.5072	0.0143	O	4.4563	1.7198	0.1437
O	-3.8204	1.8756	-0.1105	O	4.187	-2.9899	-0.1548
O	6.5317	-0.6729	0.0065	O	-0.5048	3.0561	0.1587
H	1.1269	2.5621	-0.1774	H	5.4115	-0.7091	0.0246
H	-1.2897	-2.705	0.1747	H	-2.5158	2.4163	-0.7107
H	3.074	2.0919	0.7189	H	-1.7581	-1.6344	0.5635
H	5.4774	1.5697	0.6967	H	0.715	-2.8491	-0.7557
H	4.6168	-2.3956	-0.6867	H	3.835	2.4805	0.174
H	2.2092	-1.8836	-0.6718	H	3.5173	-3.6824	-0.2584
H	-4.8333	-2.6593	0.1356	H	0.1985	3.724	0.2391
H	-5.5855	0.3849	-0.0622	O	-6.043	-0.344	-0.1447
H	-3.1444	2.5924	-0.1515	H	-6.1891	-1.2642	0.114
H	6.7124	-1.5741	-0.2819	O	-4.3547	-2.3104	0.565
				H	-3.6644	-2.9456	0.7907
				H	-4.94	1.9302	-0.7466

Table S3. (cont.)

Epicatechin				Kaempferol			
N_i = 0; E = -1031.09796				N_i = 0; E = -1028.94154			
O	-0.7521	0.3034	-1.7342	O	1.8105	2.8523	-0.1725
C	0.3915	1.0369	-1.2833	C	1.3166	1.7046	-0.0832
C	-1.7673	0.05	-0.849	C	2.0938	0.5022	-0.0083
C	1.4416	0.1347	-0.6657	C	-0.1187	1.5436	-0.0731
C	-0.0565	2.187	-0.3571	C	1.4422	-0.735	0.0699
C	-1.8353	0.6506	0.4083	C	3.5113	0.5086	-0.0221
C	-2.7346	-0.8507	-1.2985	O	-0.8609	2.6767	-0.1856
C	2.7776	0.5543	-0.6978	C	-0.6853	0.3138	0.0168
C	1.1324	-1.0726	-0.0447	O	0.0915	-0.7998	0.0913
C	-0.7918	1.6389	0.8551	C	2.129	-1.936	0.1333
O	1.0284	3.0225	-0.0043	O	4.1849	1.6739	-0.098
C	-2.9154	0.2954	1.2273	C	4.2163	-0.6751	0.0411
C	-3.7977	-1.1614	-0.4586	C	-2.1165	-0.0048	0.014
C	3.781	-0.2027	-0.1087	C	3.5166	-1.8862	0.1166
C	2.138	-1.8392	0.5432	C	-2.5566	-1.2132	-0.5416
C	-3.8983	-0.597	0.8132	C	-3.0618	0.864	0.5676
O	-2.9543	0.8742	2.4618	O	4.173	-3.0697	0.1766
O	-4.7812	-2.0327	-0.8297	C	-3.9005	-1.5402	-0.5555
C	3.4594	-1.4142	0.522	C	-4.4095	0.5399	0.5625
O	4.4898	-2.118	1.0801	C	-4.8305	-0.662	-0.0019
H	0.8058	1.4842	-2.1903	O	-6.1423	-1.0294	-0.036
H	-0.7381	2.813	-0.9414	H	-0.2299	3.4071	-0.2775
H	-2.644	-1.2969	-2.283	H	1.5997	-2.8776	0.192
H	3.047	1.4831	-1.1943	H	3.5239	2.3974	-0.1445
H	0.1122	-1.4374	-0.0127	H	5.3002	-0.6646	0.0286
H	-0.0685	1.1625	1.5316	H	-1.8413	-1.9008	-0.9762
H	-1.252	2.4633	1.4062	H	-2.7479	1.794	1.0225
H	1.6058	2.5225	0.5867	H	5.1254	-2.9272	0.1495
H	-4.7288	-0.8533	1.4626	H	-4.2397	-2.4715	-0.9935
H	-3.7403	0.5694	2.9311	H	-5.1364	1.2147	1.0017
H	-4.6009	-2.3543	-1.7212	H	-6.6866	-0.3489	0.3742
H	4.1383	-2.9255	1.4743				
O	5.098	0.1622	-0.1141				
H	5.1905	0.998	-0.587				
H	1.8974	-2.7826	1.0253				

Table S3. (cont.)

Eriodictyol				Apigenin			
N_i = 0; E = -1030.16395				N_i = 0; E = -953.71779			
C	3.7126	1.7945	-0.0232	O	1.8051	2.9807	-0.1619
C	4.4184	0.5954	0.0553	C	1.2633	1.8522	-0.0928
C	3.71	-0.594	0.0795	C	2.0455	0.6365	-0.0226
C	2.2939	-0.6048	0.0185	C	-0.161	1.6784	-0.0935
C	1.6252	0.6377	-0.0775	C	1.3981	-0.6018	0.0511
C	2.3198	1.8313	-0.0939	C	3.4609	0.6381	-0.0324
C	1.5394	-1.8284	0.1567	C	-0.7151	0.4449	-0.0055
C	0.0454	-1.6856	0.2058	O	0.0388	-0.6721	0.0701
C	-0.4139	-0.4765	-0.594	C	2.0794	-1.8032	0.1129
O	0.276	0.7109	-0.1356	O	4.1359	1.8022	-0.1033
C	-1.8894	-0.2086	-0.4941	C	4.1675	-0.5484	0.0289
C	-2.686	-0.2355	-1.632	C	-2.1523	0.1471	0.0042
C	-4.0552	0.0014	-1.5404	C	3.4691	-1.757	0.0997
C	-4.6309	0.2749	-0.3115	C	-2.6161	-1.1185	-0.3733
C	-3.8316	0.3061	0.8384	C	-3.0825	1.12	0.382
C	-2.4731	0.0625	0.7456	O	4.1212	-2.9436	0.1587
O	-5.9745	0.5148	-0.2352	C	-3.9701	-1.4019	-0.3849
O	-4.4789	0.5835	2.0116	C	-4.4391	0.8435	0.3776
O	2.081	-2.9367	0.2723	C	-4.8848	-0.4204	-0.0078
O	4.3976	-1.7463	0.1772	O	-6.2048	-0.7517	-0.0308
O	4.3511	2.9875	-0.0458	H	-0.7904	2.5531	-0.182
H	5.5008	0.5875	0.106	H	1.5476	-2.7436	0.1687
H	1.7949	2.7742	-0.174	H	3.4621	2.5209	-0.1426
H	-0.423	-2.5925	-0.1789	H	5.2514	-0.5368	0.0197
H	-0.2357	-1.5772	1.2603	H	-1.9137	-1.8862	-0.6729
H	-0.143	-0.6157	-1.6458	H	-2.751	2.1012	0.7008
H	-2.2411	-0.4416	-2.5993	H	5.0741	-2.804	0.1345
H	-4.6844	-0.018	-2.4228	H	-4.329	-2.3791	-0.6853
H	-1.8715	0.0917	1.649	H	-5.155	1.5998	0.6807
H	-6.2126	0.6974	0.6813	H	-6.7424	-0.0055	0.2551
H	-3.8575	0.5803	2.7473				
H	3.7407	-2.4764	0.2219				
H	5.305	2.859	0.0001				

Table S3. (cont.)

Quercetin				Liquiritigenin			
N_i = 0; E = -1104.17117				N_i = 0; E = -879.69586			
C	0.6453	-0.2112	0.1594	C	-0.4742	0.4842	0.4414
O	-0.6431	0.2098	0.0482	C	-0.068	1.761	-0.2791
C	1.4203	2.0719	-0.2189	C	1.404	2.0355	-0.1163
C	-0.9592	1.5004	-0.2029	O	1.8498	3.1779	-0.1299
C	1.6697	0.6700	0.0278	C	2.2516	0.8487	-0.001
C	0.0464	2.4661	-0.3312	C	3.6525	0.946	0.0273
C	-2.3051	1.8054	-0.3195	C	4.4515	-0.1724	0.0336
C	-2.6466	3.1268	-0.5761	C	3.846	-1.438	0.0069
H	-3.0608	1.0379	-0.2189	C	2.4653	-1.5733	-0.0098
C	-1.6794	4.1316	-0.7087	C	1.6714	-0.4313	-0.0069
H	-1.9718	5.1560	-0.9084	O	0.3316	-0.6247	-0.024
C	-0.3450	3.8040	-0.5903	C	-1.9171	0.1143	0.2435
O	2.3857	2.8605	-0.3398	C	-2.7809	0.0537	1.3321
C	0.7398	-1.6558	0.3957	C	-4.1252	-0.2595	1.1635
C	0.8632	-4.4121	0.8284	C	-4.6114	-0.5248	-0.1123
C	-0.2858	-2.4908	-0.0691	C	-3.7563	-0.4806	-1.2127
C	1.8165	-2.2169	1.0857	C	-2.4213	-0.1574	-1.0302
C	1.8724	-3.5874	1.2992	O	4.667	-2.5165	0.0093
C	-0.2246	-3.8542	0.1452	O	-5.9234	-0.8358	-0.3461
H	-1.1345	-2.0827	-0.6059	H	-0.2719	0.5953	1.5124
H	2.6070	-1.5900	1.4731	H	-0.2642	1.6665	-1.3544
H	2.7016	-4.0289	1.8393	H	-0.6454	2.6063	0.0979
O	0.9400	-5.7549	1.0415	H	4.1027	1.932	0.0301
H	0.1742	-6.1858	0.6436	H	5.5317	-0.0973	0.0513
O	-1.1791	-4.7364	-0.2785	H	2.0019	-2.5532	-0.0121
H	-1.9024	-4.2689	-0.7085	H	-2.4046	0.2553	2.3297
O	-3.9671	3.4054	-0.6949	H	-4.7935	-0.2998	2.0174
H	-4.0990	4.3394	-0.8913	H	-4.1474	-0.6932	-2.2011
O	0.5851	4.7704	-0.7248	H	-1.7639	-0.1193	-1.8927
H	1.4660	4.3517	-0.6227	H	4.1501	-3.3298	-0.0098
O	2.9710	0.2864	0.0938	H	-6.4211	-0.8077	0.4778
H	3.4994	1.0823	-0.0705				

Table S3. (cont.)

Fisetin				Taxifolin			
N_i = 0; E = -1028.93476				N_i = 0; E = -1105.38614			
O	-5.0532	-2.509	-0.288	C	2.3585	0.4811	-0.0106
C	-4.1971	-1.4629	-0.1863	C	1.7196	-0.7792	-0.0477
C	-2.8251	-1.6326	-0.183	C	2.4412	-1.9536	-0.0051
C	-4.7714	-0.1809	-0.0829	C	3.8319	-1.8766	0.0908
C	-2.0206	-0.5016	-0.0702	C	4.5053	-0.6578	0.1602
C	-3.959	0.9181	0.0257	C	3.7695	0.5133	0.1124
C	-2.5578	0.7822	0.0347	C	1.5827	1.6815	-0.1619
O	-0.6851	-0.7138	-0.0737	C	0.1115	1.4906	-0.4733
C	-1.6603	1.9091	0.1572	C	-0.403	0.261	0.2728
C	0.1978	0.3123	0.0483	O	0.3722	-0.8828	-0.1408
O	-2.0167	3.0934	0.2748	C	-1.8494	-0.042	0.0035
C	-0.2457	1.59	0.161	C	-2.2913	-0.3428	-1.2826
C	1.5891	-0.1542	0.0759	C	-3.6334	-0.6124	-1.5164
O	0.6054	2.6376	0.3199	C	-4.545	-0.5737	-0.471
C	1.879	-1.4303	0.5662	C	-4.1062	-0.2691	0.8199
C	2.6338	0.6534	-0.3898	C	-2.7641	-0.009	1.0532
C	3.1881	-1.8876	0.6032	O	-5.0657	-0.253	1.7958
C	3.9366	0.1909	-0.3552	O	-5.8629	-0.8372	-0.7206
C	4.2214	-1.0842	0.1462	O	-0.6429	2.6206	-0.1097
O	5.0114	0.9115	-0.7965	O	2.0457	2.8236	-0.081
O	5.5016	-1.5483	0.1885	O	4.4287	1.6848	0.1699
H	-4.5658	-3.3385	-0.3446	O	4.4996	-3.0507	0.1311
H	-2.3753	-2.6152	-0.2626	H	1.9403	-2.9124	-0.028
H	-5.8502	-0.0848	-0.0898	H	5.5846	-0.6218	0.2493
H	-4.3879	1.9096	0.108	H	0.0458	1.3018	-1.5558
H	0.0431	3.4225	0.4155	H	-0.2462	0.4082	1.3471
H	1.0839	-2.0676	0.9309	H	-1.5901	-0.3722	-2.1092
H	2.4414	1.6391	-0.7944	H	-3.9885	-0.8516	-2.5123
H	3.4205	-2.8727	0.9907	H	-2.4389	0.221	2.0632
H	4.7322	1.7808	-1.1009	H	-4.6772	-0.0168	2.6445
H	6.0974	-0.8737	-0.159	H	-6.3631	-0.765	0.1007
				H	-0.0515	3.3833	-0.1504
				H	3.7726	2.409	0.1222
				H	5.4475	-2.8946	0.206

Table S3. (cont.)

Hesperetin				3,3',4'-Trihydroxyflavone			
N_i = 0; E = -1069.45336				N_i = 0; E = -953.46443			
O	-1.8939	-2.8917	0.5853	C	-0.8325	1.5388	-0.1318
C	-1.6412	-1.9675	-0.1993	C	-0.0944	0.4077	0.0351
C	-2.0347	-0.6002	0.0558	C	-2.2824	1.508	-0.1671
C	-0.9453	-2.2212	-1.5068	O	-2.9092	2.5689	-0.3303
C	-1.7513	0.4079	-0.8929	C	-2.8913	0.1965	-0.0212
C	-2.7693	-0.2365	1.2119	C	-4.2844	0.0062	-0.0403
C	-0.1646	-1.0011	-1.9634	C	-4.8129	-1.2605	0.0908
O	-1.0624	0.1344	-2.0263	C	-3.9571	-2.3655	0.2422
C	-2.1855	1.7072	-0.7173	C	-2.5852	-2.2046	0.2633
O	-3.0679	-1.1561	2.1491	C	-2.0605	-0.9161	0.1305
C	-3.2059	1.0626	1.4027	O	-0.7099	-0.8003	0.1636
C	1.0435	-0.6379	-1.1173	C	1.3685	0.2951	0.0783
C	-2.9148	2.0179	0.432	C	2.1742	1.3564	0.5014
C	1.6923	0.5725	-1.3903	C	3.5573	1.2144	0.5417
C	1.5303	-1.4462	-0.1002	C	4.1521	0.0192	0.162
O	-3.3622	3.2749	0.6592	C	3.3475	-1.0525	-0.2587
C	2.7916	0.961	-0.6539	C	1.9717	-0.917	-0.2969
C	2.6461	-1.0614	0.6476	O	4.0177	-2.1883	-0.6162
O	3.4088	2.148	-0.9349	O	5.5066	-0.1048	0.2026
C	3.2775	0.1407	0.3786	O	-0.2702	2.7637	-0.3017
O	4.364	0.6353	1.0312	H	-4.9248	0.8734	-0.1601
C	4.9026	-0.1283	2.1061	H	-5.8868	-1.4083	0.0767
H	-0.3139	-3.1065	-1.4349	H	-4.3765	-3.3607	0.3445
H	-1.7263	-2.4323	-2.2457	H	-1.9102	-3.0449	0.3794
H	0.1459	-1.1351	-2.9992	H	1.7282	2.2912	0.814
H	-1.9497	2.4668	-1.4528	H	1.3695	-1.7567	-0.6281
H	-2.7123	-2.0173	1.8396	H	-1.0267	3.3652	-0.4234
H	-3.7681	1.3293	2.2885	H	3.3926	-2.8671	-0.8982
H	1.3368	1.2252	-2.18	H	5.7425	-0.9928	-0.0986
H	1.0622	-2.3934	0.1372	H	4.1887	2.0312	0.8743
H	-3.1099	3.8564	-0.0667				
H	3.0054	-1.7082	1.4374				
H	4.1543	2.2572	-0.3316				
H	5.7443	0.4465	2.4877				
H	4.1595	-0.2621	2.897				
H	5.2524	-1.1025	1.7537				

Table S3. (cont.)

Luteolin				Morin			
N_i = 0; E = -1028.94743				N_i = 0; E = -1103.88849			
O	-4.1377	3.0981	-0.2216	C	4.3367	-0.6114	-0.1366
C	-3.5766	1.8669	-0.1406	C	3.6448	-1.8206	-0.3056
C	-4.362	0.7153	-0.0368	C	2.2531	-1.8775	-0.3286
C	-3.7453	-0.5185	0.0517	C	1.556	-0.6883	-0.1761
O	-4.5049	-1.6264	0.1563	C	2.2024	0.5454	-0.0018
C	-2.3338	-0.623	0.0339	C	3.6221	0.5625	0.0154
C	-1.6432	-1.8906	0.1393	O	0.2039	-0.7591	-0.209
O	-2.2656	-2.9736	0.245	C	-0.574	0.35	-0.0399
C	-0.2097	-1.8228	0.129	C	-0.0137	1.5714	0.1429
C	0.4335	-0.6379	-0.0042	C	1.4246	1.7386	0.1628
O	-0.2364	0.5293	-0.1074	C	-2.0145	0.0742	-0.1014
C	-1.597	0.5612	-0.0768	C	-2.8439	0.846	-0.922
C	-2.1874	1.8082	-0.1656	C	-4.2082	0.619	-1.0036
C	1.8897	-0.4503	-0.0451	C	-4.7677	-0.41	-0.2414
C	2.7384	-1.5098	-0.3705	C	-3.9691	-1.195	0.5874
C	4.1121	-1.3216	-0.4008	C	-2.6012	-0.9528	0.6624
C	4.6544	-0.0785	-0.1092	O	-6.0971	-0.6924	-0.2645
O	6.0037	0.0883	-0.1474	O	1.9123	2.8827	0.3359
C	3.8074	0.9891	0.2137	O	-0.7623	2.6901	0.3327
O	4.4218	2.1787	0.4862	O	4.2807	1.7242	0.1812
C	2.4382	0.8052	0.2432	O	4.3047	-2.9917	-0.4548
H	-5.098	3.0347	-0.1758	H	5.4212	-0.5923	-0.1219
H	-5.4437	0.7838	-0.0214	H	1.7311	-2.8167	-0.4601
H	-3.8867	-2.3917	0.2124	H	-4.8366	1.2232	-1.6498
H	0.3518	-2.7396	0.2407	H	-4.4195	-1.9837	1.1817
H	-1.5875	2.7048	-0.2476	H	-6.5477	-0.0755	-0.8546
H	2.3366	-2.4832	-0.6226	H	-0.1224	3.4099	0.4644
H	4.7772	-2.1363	-0.6614	H	3.601	2.431	0.2777
H	6.2187	1.0057	0.0603	H	5.2579	-2.8428	-0.4124
H	3.7683	2.8648	0.6571	O	-1.7976	-1.6752	1.4845
H	1.8027	1.6444	0.5013	H	-2.325	-2.3297	1.9593
				H	-2.4008	1.6386	-1.5154

Table S3. (cont.)

Epigallocatechin				5,3',4'-Trihydroxyflavone			
N_i = 0; E = -1106.59312				N_i = 0; E = -953.71227			
O	0.7627	3.1915	0.4934	C	0.15	-0.4605	-0.0111
C	-0.28	2.3793	-0.0155	C	-0.5884	-1.5833	0.171
C	0.2371	1.4216	-1.1063	C	-2.0201	-1.5325	0.1882
C	-1.009	1.6177	1.0767	O	-2.7308	-2.55	0.3465
O	-0.8639	0.7083	-1.6834	C	-2.6084	-0.2118	0.0239
C	1.3392	0.4913	-0.6428	C	-4.0061	0.0016	0.0327
C	-1.9877	0.6553	0.4615	C	-4.5155	1.2809	-0.1261
C	-1.8674	0.2559	-0.8674	C	-3.6427	2.3542	-0.2904
C	2.6575	0.9268	-0.7557	C	-2.2673	2.1831	-0.3004
C	1.0716	-0.7591	-0.0867	C	-1.773	0.8966	-0.1421
C	-3.0473	0.1104	1.1942	O	-0.4176	0.751	-0.1641
C	-2.7635	-0.6296	-1.4639	C	1.6165	-0.4028	-0.0574
C	3.6997	0.1252	-0.3075	C	2.2772	0.7855	0.2763
C	2.1186	-1.5573	0.3531	C	3.6577	0.8428	0.25
O	-3.1443	0.497	2.5011	C	4.4015	-0.2837	-0.1247
C	-3.9567	-0.7777	0.6359	C	3.747	-1.4584	-0.4646
C	-3.8026	-1.1378	-0.6996	C	2.3619	-1.5221	-0.4301
O	4.985	0.5733	-0.436	O	5.7604	-0.2416	-0.1631
C	3.4373	-1.1216	0.2508	O	4.3812	1.9543	0.5774
O	1.9457	-2.7974	0.9035	O	-4.8552	-1.0348	0.197
O	-4.7144	-2.0186	-1.2157	H	-0.1006	-2.536	0.3243
O	4.4997	-1.8772	0.6672	H	-5.5883	1.4297	-0.1191
H	1.3856	2.6315	0.9697	H	-4.0513	3.3505	-0.4135
H	-0.9751	3.0655	-0.505	H	-1.5847	3.0132	-0.4279
H	0.6251	2.0299	-1.9241	H	1.7209	1.6672	0.5734
H	-0.2781	1.0805	1.6952	H	4.3343	-2.3189	-0.7626
H	-1.5241	2.3257	1.729	H	1.8688	-2.4424	-0.7172
H	2.89	1.8856	-1.2052	H	6.062	0.6389	0.0907
H	0.0603	-1.138	0.0071	H	3.7964	2.6745	0.8348
H	-2.6344	-0.9178	-2.5008	H	-4.3044	-1.8445	0.2949
H	-3.9195	0.0927	2.9036				
H	-4.7691	-1.1857	1.2267				
H	5.5955	-0.108	-0.1313				
H	1.0115	-3.0302	0.9126				
H	-4.5019	-2.2065	-2.1358				
H	4.1876	-2.7423	0.9557				

Table S3. (cont.)

Ampelopsin				Myricetin			
N_i = 0; E = -1180.61604				N_i = 0; E = -1179.40046			
C	0.3165	1.4881	-0.47	C	1.8283	-1.7172	-0.2251
C	1.8054	1.6742	-0.2563	O	2.3459	-2.8437	-0.4009
C	2.5683	0.4758	-0.041	C	2.5788	-0.5096	-0.0456
C	1.9084	-0.7686	0.0761	C	0.3884	-1.5881	-0.2125
O	0.5562	-0.8522	0.0661	C	1.9012	0.7041	0.1246
C	-0.178	0.3402	0.4081	C	3.9964	-0.487	-0.0414
C	3.9845	0.489	-0.0095	O	-0.3251	-2.7253	-0.4162
C	4.7029	-0.6881	0.1036	C	-0.203	-0.3807	-0.0303
C	4.0079	-1.8935	0.1928	O	0.5494	0.7395	0.1354
C	2.6126	-1.949	0.188	C	2.5621	1.9092	0.2954
O	4.6578	-3.0732	0.3043	O	4.6951	-1.6283	-0.2028
O	4.6629	1.6466	-0.1065	C	4.6755	0.7009	0.1284
O	2.2914	2.8085	-0.3083	C	-1.6423	-0.0913	-0.013
O	-0.4004	2.6597	-0.1682	C	3.9507	1.8884	0.2939
C	-1.6418	0.0462	0.2352	C	-2.09	1.1551	-0.4575
C	-2.1346	-0.3562	-1.0038	C	-2.5568	-1.0369	0.4575
C	-3.4897	-0.6066	-1.1545	O	4.5814	3.0754	0.4618
C	-4.3609	-0.4487	-0.0773	C	-3.4444	1.4455	-0.4403
C	-3.8603	-0.0454	1.1564	C	-3.9085	-0.7313	0.4748
C	-2.5015	0.1998	1.3173	O	-3.8682	2.6645	-0.8862
O	-3.9648	-1.0041	-2.3726	C	-4.3632	0.5064	0.025
O	-5.6868	-0.704	-0.2898	O	-4.87	-1.5899	0.9262
O	-4.7794	0.0801	2.1603	O	-5.6814	0.852	0.0209
H	0.1901	1.207	-1.5269	H	0.3244	-3.4323	-0.5516
H	0.0313	0.5808	1.4562	H	2.0127	2.832	0.4252
H	5.7862	-0.6674	0.1196	H	4.0503	-2.3584	-0.3166
H	2.0961	-2.895	0.284	H	5.7593	0.7118	0.131
H	5.6115	-2.9345	0.3019	H	-1.3992	1.9021	-0.8264
H	4.014	2.3749	-0.188	H	-2.2315	-1.9999	0.8285
H	0.1912	3.4061	-0.3278	H	5.5374	2.9582	0.4334
H	-1.4787	-0.4801	-1.8579	H	-4.8277	2.7196	-0.8079
H	-2.1267	0.5114	2.2866	H	-4.4678	-2.4167	1.2119
H	-4.9192	-1.131	-2.3186	H	-6.2133	0.1249	0.3643
H	-6.1783	-0.5789	0.53				
H	-4.3489	0.3722	2.9701				

Table S3. (cont.)

Wogonin				7,8-Dihydroxyflavone			
N_i = 0; E = -992.99634				N_i = 0; E = -878.47167			
C	3.7176	0.1102	-0.095	O	0.1045	-0.4228	0.0472
C	2.9104	1.2297	-0.0409	O	-1.074	3.4631	-0.1687
C	1.5011	1.1022	-0.0335	C	-1.2209	-0.1282	0.0227
C	0.9536	-0.1845	-0.0819	C	-1.7012	1.1787	-0.0419
C	1.748	-1.3219	-0.1272	C	-0.7317	2.2734	-0.1066
C	3.1335	-1.1561	-0.1355	C	0.6541	1.8669	-0.1053
C	0.6125	2.244	0.0116	C	1.0181	0.5671	-0.0209
C	-0.794	1.9439	-0.0047	C	-3.088	1.3776	-0.0498
C	-1.2354	0.666	-0.0405	C	-2.075	-1.2279	0.0722
O	-0.3863	-0.3814	-0.0812	C	-3.4456	-1.0016	0.0643
O	1.0479	3.4165	0.0527	C	-3.948	0.303	0.0041
O	3.4815	2.4516	0.0019	C	2.3975	0.0494	-0.0053
O	3.943	-2.2355	-0.1871	O	-4.3219	-2.0372	0.1139
O	1.2205	-2.5851	-0.2135	O	-1.6296	-2.5127	0.1325
C	0.7502	-3.1127	1.0386	C	3.4614	0.869	0.3834
C	-2.6444	0.2378	-0.0547	C	4.7579	0.3736	0.3841
C	-3.6464	1.077	0.4416	C	5.0062	-0.941	0.0007
C	-4.9727	0.6698	0.4131	C	3.9504	-1.7621	-0.3808
C	-5.3123	-0.5754	-0.1075	C	2.6509	-1.2732	-0.3819
C	-4.318	-1.4163	-0.5964	H	1.4123	2.6337	-0.1873
C	-2.9888	-1.0164	-0.568	H	-3.4758	2.3873	-0.1007
H	4.7958	0.2095	-0.1039	H	-5.0221	0.4438	-0.0014
H	-1.4976	2.7646	-0.0021	H	-3.8438	-2.8748	0.1425
H	2.7493	3.1091	0.0329	H	-0.6668	-2.5361	0.0763
H	3.4002	-3.0344	-0.2342	H	3.2811	1.8883	0.7037
H	0.4097	-4.1267	0.8343	H	5.5757	1.0144	0.6926
H	1.5637	-3.1331	1.7685	H	6.02	-1.3251	0.0037
H	-0.0802	-2.5137	1.4199	H	4.1375	-2.787	-0.6798
H	-3.394	2.0407	0.8681	H	1.8337	-1.9166	-0.6843
H	-5.7422	1.3242	0.806				
H	-6.3493	-0.8907	-0.1281				
H	-4.5769	-2.3872	-1.003				
H	-2.2197	-1.6736	-0.9546				

Table S3. (cont.)

Chrysin				Pinocembrin			
N_i = 0; E = -878.48325				N_i = 0; E = -879.69920			
C	-0.8076	1.8407	-0.1086	C	0.2185	1.9405	0.3212
O	-1.3001	2.9896	-0.186	O	0.308	2.9623	-0.3719
C	-1.6374	0.6583	-0.0314	C	1.065	0.7854	0.12
C	0.6109	1.6079	-0.1027	C	-0.7513	1.8467	1.4644
C	-1.0414	-0.6056	0.0525	C	0.9282	-0.3492	0.9533
C	-3.0521	0.7179	-0.0401	C	2.1007	0.7777	-0.8453
C	1.1097	0.3543	-0.0076	C	-1.2037	0.4087	1.6643
O	0.3141	-0.7321	0.0715	O	-0.0421	-0.4141	1.8982
C	-1.7712	-1.777	0.1269	C	1.7833	-1.427	0.8538
O	-3.6792	1.9074	-0.12	O	2.2799	1.8282	-1.6674
C	-3.8065	-0.438	0.0324	C	2.9635	-0.3	-0.9599
C	2.5398	0.0019	0.0047	C	-2.0287	-0.1768	0.525
C	-3.1577	-1.6733	0.1154	C	2.7996	-1.3852	-0.1024
C	2.9574	-1.253	-0.4481	C	-2.7817	0.6288	-0.3282
C	3.4889	0.9207	0.4612	C	-2.0644	-1.562	0.3478
O	-3.8584	-2.8302	0.19	O	3.6147	-2.4635	-0.1669
C	4.3078	-1.5757	-0.4559	C	-3.5526	0.0618	-1.3395
C	4.8369	0.5902	0.4554	C	-2.8305	-2.1272	-0.6636
C	5.2496	-0.6565	-0.0051	C	-3.5784	-1.3163	-1.5124
H	1.2787	2.4536	-0.1916	H	-0.2191	2.1716	2.3654
H	-1.2788	-2.7379	0.1924	H	-1.5865	2.5292	1.3144
H	-2.978	2.5984	-0.1648	H	-1.7687	0.3318	2.5932
H	-4.889	-0.3821	0.024	H	1.6588	-2.2918	1.4923
H	2.2289	-1.97	-0.8061	H	1.607	2.5038	-1.4341
H	3.1755	1.8867	0.8391	H	3.7538	-0.2899	-1.7013
H	-4.8041	-2.6483	0.1756	H	-2.7787	1.7065	-0.2176
H	4.6246	-2.5475	-0.8166	H	-1.484	-2.2027	1.0014
H	5.5656	1.3055	0.8187	H	4.2776	-2.3414	-0.8556
H	6.3032	-0.9121	-0.0089	H	-4.1301	0.7044	-1.9946
				H	-2.8431	-3.2042	-0.789
				H	-4.1755	-1.7569	-2.3029

Table S3. (cont.)

Catechin				Eupatilin			
N_i = 0; E = -1031.09808				N_i = 0; E = -1222.03662			
O	-0.8696	3.2095	-0.6197	C	4.4539	0.4367	-0.0462
C	-0.0194	2.2182	-0.0626	C	3.8	1.6644	0.1412
C	0.4177	1.2178	-1.1423	C	2.4159	1.7588	0.1394
C	-0.6776	1.4741	1.0937	C	1.6939	0.5949	-0.0549
O	-0.7148	0.5047	-1.6498	C	2.2994	-0.6492	-0.255
C	1.5101	0.2816	-0.6633	C	3.7125	-0.7148	-0.255
C	-1.6963	0.4971	0.5661	O	0.3374	0.7106	-0.0378
C	-1.6614	0.0657	-0.7603	C	-0.4531	-0.3632	-0.2374
C	1.3129	-1.0847	-0.5003	C	0.0592	-1.5991	-0.456
C	2.7661	0.8258	-0.3688	C	1.4759	-1.8216	-0.468
C	-2.7233	-0.013	1.3729	O	4.5295	2.7847	0.3263
C	-2.6027	-0.8218	-1.2842	O	5.8275	0.4307	-0.0662
C	2.3522	-1.8945	-0.039	C	6.4272	-0.0271	1.1577
C	3.8024	0.0249	0.0865	O	4.3513	-1.8796	-0.4574
O	-2.7402	0.4107	2.6693	O	1.9813	-2.9525	-0.6595
C	-3.6763	-0.9015	0.8875	C	-1.8796	-0.0172	-0.1996
C	-3.607	-1.296	-0.4491	C	-2.2953	1.301	-0.3794
C	3.5944	-1.3534	0.2582	C	-3.6437	1.633	-0.3664
O	-4.5617	-2.1671	-0.889	C	-4.6055	0.6464	-0.1634
H	-1.7116	2.7943	-0.8421	C	-4.1879	-0.6839	0.0289
H	0.8658	2.751	0.2911	C	-2.8484	-1.0067	0.0103
H	0.7811	1.788	-2.0015	O	-5.1297	-1.6711	0.2067
H	0.0932	0.9541	1.6745	C	-5.5512	-1.819	1.5674
H	-1.1475	2.1998	1.7626	O	-5.9369	0.8619	-0.1379
H	2.9498	1.8894	-0.5042	C	-6.4055	2.1983	-0.3081
H	-2.5389	-1.1289	-2.3226	H	1.919	2.7084	0.2868
H	-3.5016	0.0262	3.1203	H	-0.5971	-2.4379	-0.6413
H	-4.4615	-1.2836	1.5314	H	5.4671	2.5562	0.2605
H	-4.4138	-2.3562	-1.8236	H	6.1141	0.6069	1.9917
H	0.3571	-1.5389	-0.7341	H	6.153	-1.0657	1.3551
H	2.2	-2.9626	0.0892	H	7.5044	0.0502	1.0183
O	5.0481	0.5053	0.3793	H	3.6526	-2.5649	-0.5806
O	4.6541	-2.0901	0.7099	H	-1.5665	2.0836	-0.546
H	4.3844	-3.014	0.7773	H	-3.9347	2.6635	-0.5198
H	5.0639	1.4555	0.2119	H	-2.5747	-2.0417	0.1782
				H	-6.3088	-2.6017	1.5753
				H	-4.7058	-2.1175	2.1948
				H	-5.9812	-0.8874	1.9456
				H	-7.4901	2.1399	-0.2428
				H	-6.0238	2.8478	0.484
				H	-6.1171	2.5907	-1.2867

Table S3. (cont.)

Baicalein				Pectolinarigenin			
N_i = 0; E = -953.70820				N_i = 0; E = -1107.52139			
C	-0.7443	-0.585	0.0453	C	0.9422	0.6233	-0.0505
C	-1.3259	0.6832	-0.0359	C	0.3422	1.8349	0.0538
C	-2.7342	0.7724	-0.043	C	-1.0856	1.9594	0.0325
C	-3.5033	-0.3742	0.0298	O	-1.6694	3.0661	0.1157
C	-2.8783	-1.6263	0.1107	C	-1.8241	0.7185	-0.0774
C	-1.4962	-1.7438	0.1186	C	-3.2381	0.6871	-0.0974
O	0.6118	-0.7283	0.0639	C	-3.8968	-0.5275	-0.1937
C	1.4157	0.3486	-0.0111	C	-3.1585	-1.7174	-0.2891
C	0.9293	1.6092	-0.1048	C	-1.7711	-1.7153	-0.2775
C	-0.4842	1.8586	-0.1122	C	-1.1323	-0.4931	-0.1679
C	2.8436	-0.0143	0.0042	O	0.2292	-0.516	-0.1628
C	3.2529	-1.2738	-0.4435	C	2.3891	0.3775	-0.0412
C	4.6009	-1.6067	-0.4471	C	2.8914	-0.8858	0.2729
C	5.5487	-0.6929	0.0024	C	4.2581	-1.129	0.3062
C	5.1443	0.5584	0.4577	C	5.1464	-0.0947	0.0131
C	3.7988	0.8988	0.4595	C	4.655	1.1742	-0.3125
O	-0.9692	3.011	-0.1887	C	3.2954	1.4052	-0.3381
O	-3.3687	1.9619	-0.1188	O	-5.2667	-0.61	-0.253
O	-4.87	-0.3559	0.0286	C	-5.914	-0.502	1.0259
O	-3.6258	-2.7518	0.1851	O	6.4948	-0.2264	0.0142
H	-1.0238	-2.715	0.1831	C	7.0482	-1.5015	0.3304
H	1.607	2.4474	-0.1907	O	-3.8092	-2.8947	-0.4001
H	2.5199	-1.9866	-0.8006	O	-3.9565	1.8207	-0.0274
H	4.9112	-2.582	-0.8037	H	0.936	2.7307	0.1707
H	6.6003	-0.9564	0.0018	H	-1.209	-2.6366	-0.3531
H	5.8775	1.2696	0.8199	H	2.2123	-1.6957	0.5083
H	3.4924	1.8686	0.8334	H	4.6125	-2.1185	0.5616
H	-2.6671	2.6524	-0.1642	H	5.3554	1.9661	-0.5502
H	-5.1858	0.5543	0	H	2.9387	2.3917	-0.6093
H	-4.5631	-2.5217	0.1762	H	-6.981	-0.6146	0.8398
				H	-5.5672	-1.2965	1.6924
				H	-5.7175	0.474	1.4752
				H	8.1277	-1.3789	0.2673
				H	6.773	-1.8064	1.3436
				H	6.7227	-2.2577	-0.389
				H	-4.7599	-2.7179	-0.4321
				H	-3.3069	2.5601	0.0433

Table S3. (cont.)

3,5-Dihydroxyflavone				Alpinetin			
N_i = 0; E = -878.47189				N_i = 0; E = -918.98149			
C	-0.1532	1.3062	-0.0909	C	1.362	-0.3214	0.403
C	-0.7951	0.1154	0.0205	O	0.6483	0.8555	-0.0225
C	1.2891	1.3756	-0.1005	C	-0.7025	0.78	-0.0047
O	1.856	2.4833	-0.213	C	-2.7452	2.0076	0.0399
C	1.9909	0.1218	0.0064	C	-1.359	2.002	0.0165
C	3.4055	0.0428	0.003	O	-3.4545	3.1607	0.0633
C	4.0284	-1.1878	0.1011	C	-3.4842	0.8244	0.0608
C	3.2555	-2.3463	0.2	C	-2.8204	-0.391	0.0343
C	1.8725	-2.3072	0.2043	O	-3.4776	-1.5624	0.055
C	1.2566	-1.0648	0.1063	C	-4.9035	-1.5515	0.0983
O	-0.097	-1.0424	0.1175	C	-1.3966	-0.4519	-0.0234
C	-2.2489	-0.1051	0.0176	C	-0.6391	-1.6884	-0.2125
C	-3.1208	0.813	0.6099	O	-1.1302	-2.8107	-0.2934
C	-4.4895	0.5766	0.6013	C	2.8325	-0.0661	0.2101
C	-5	-0.5694	0.0009	C	0.8526	-1.5175	-0.3752
C	-4.1349	-1.4879	-0.5865	C	3.3271	0.3348	-1.0318
C	-2.7657	-1.2622	-0.5755	C	3.7166	-0.2627	1.2674
O	4.1548	1.1621	-0.094	C	5.0831	-0.0667	1.0877
O	-0.8248	2.4776	-0.2269	C	5.5709	0.3379	-0.1495
H	5.1102	-1.2378	0.0986	C	4.6893	0.5414	-1.2087
H	3.7559	-3.3048	0.2753	H	1.1515	-0.4606	1.4691
H	1.2711	-3.2036	0.28	H	-0.7903	2.9239	0.0319
H	-2.7311	1.7007	1.0906	H	-2.8599	3.9183	0.0446
H	-5.1581	1.2897	1.0698	H	-4.5618	0.8889	0.0989
H	-6.0692	-0.7487	-0.0068	H	-5.2004	-2.5983	0.1068
H	-4.5273	-2.3826	-1.0559	H	-5.2626	-1.0605	1.0062
H	-2.0959	-1.9786	-1.0357	H	-5.3174	-1.0598	-0.7856
H	3.5457	1.9262	-0.1617	H	1.3537	-2.4285	-0.0459
H	-0.1542	3.1696	-0.332	H	1.0559	-1.3816	-1.4445
				H	2.6444	0.4886	-1.861
				H	3.335	-0.5709	2.2355
				H	5.764	-0.2244	1.9167
				H	6.6344	0.4963	-0.2899
				H	5.0653	0.8584	-2.1751

Table S3. (cont.)

Galangin				Genkwanin			
N_i = 0; E = -953.70715				N_i = 0; E = -993.00741			
O	-1.2853	2.8737	-0.189	C	-1.9203	-1.4677	0.0739
C	-0.862	1.6997	-0.0974	C	-3.3056	-1.2973	0.0666
C	-1.707	0.5448	-0.0199	C	-3.885	-0.0259	0.0081
C	0.5641	1.4539	-0.0792	C	-3.0633	1.0881	-0.0427
C	-1.1299	-0.7292	0.0636	C	-1.6572	0.9565	-0.0359
C	-3.1223	0.6349	-0.0295	C	-1.1289	-0.3395	0.023
O	1.3736	2.5386	-0.1855	C	-0.7657	2.0948	-0.0919
C	1.0527	0.1934	0.0121	C	0.6361	1.7889	-0.0911
O	0.2147	-0.8747	0.0809	C	1.0728	0.5082	-0.0153
C	-1.8864	-1.8865	0.139	O	0.2195	-0.5359	0.0426
O	-3.7269	1.8366	-0.1108	C	2.4775	0.0816	0.0018
C	-3.8957	-0.5045	0.0447	C	3.4873	0.9633	0.3998
C	2.468	-0.2056	0.0153	C	4.8135	0.5668	0.4025
C	-3.2684	-1.7539	0.1296	C	5.1492	-0.7262	0.0039
C	2.8489	-1.3897	-0.6249	C	4.1548	-1.6188	-0.3913
C	3.4356	0.5706	0.6594	C	2.831	-1.2159	-0.3869
O	-3.9951	-2.8936	0.208	O	6.4368	-1.1669	-0.0126
C	4.1802	-1.7819	-0.6309	O	-1.2002	3.2694	-0.1485
C	4.7651	0.168	0.6553	O	-3.6285	2.3101	-0.0996
C	5.1413	-1.0043	0.0083	O	-4.0282	-2.4359	0.1198
H	0.7907	3.309	-0.2658	C	-5.4526	-2.3459	0.1204
H	-1.414	-2.8574	0.2054	H	-1.4817	-2.4555	0.1212
H	-3.0264	2.5209	-0.1614	H	-4.9564	0.1179	0.0024
H	-4.9769	-0.4294	0.0364	H	1.3441	2.6024	-0.1671
H	2.1038	-1.9965	-1.1255	H	3.2435	1.9658	0.7306
H	3.1494	1.477	1.177	H	5.5915	1.2522	0.721
H	-4.9363	-2.6891	0.2002	H	4.428	-2.6209	-0.7001
H	4.4675	-2.6963	-1.1373	H	2.0662	-1.9147	-0.7019
H	5.5081	0.7714	1.1642	H	7.0297	-0.4649	0.2768
H	6.1805	-1.3134	0.0044	H	-2.8913	2.964	-0.1307
				H	-5.8103	-3.3721	0.1715
				H	-5.8068	-1.7897	0.992
				H	-5.8137	-1.8768	-0.7983

Table S3. (cont.)

Primuletin				Tectochrysin			
N_i = 0; E = -803.24804				$_i$ = 0; E = -917.77285			
C	1.2395	-1.528	-0.1224	C	0.7754	-0.3607	0.0291
O	1.8567	-2.611	-0.2197	C	1.2318	0.9624	-0.0395
C	1.9391	-0.2574	-0.0127	C	0.2803	2.05	-0.0983
C	-0.1931	-1.4513	-0.1248	C	-1.105	1.6667	-0.093
C	1.2024	0.9264	0.0906	C	-1.467	0.3659	-0.0102
C	3.3506	-0.1671	-0.0129	O	-0.5601	-0.6308	0.0532
C	-0.8293	-0.2617	-0.0064	C	1.6274	-1.4435	0.0844
O	-0.1605	0.9012	0.1019	C	3.0013	-1.1977	0.0712
C	1.8065	2.1711	0.1932	C	3.5102	0.1032	0.0032
O	4.107	-1.2805	-0.1114	C	2.6292	1.1704	-0.0518
C	3.9685	1.0692	0.0893	C	-2.8517	-0.1378	0.0067
C	-2.29	-0.0729	0.0028	O	0.6473	3.2458	-0.16
C	3.1915	2.2211	0.1905	O	3.785	-2.2942	0.1299
C	-3.1332	-1.1035	0.4277	C	-3.8969	0.6779	0.4502
C	-2.842	1.1394	-0.4212	C	-5.2005	0.2017	0.4491
C	-4.5095	-0.9242	0.4196	C	-5.4742	-1.0893	0.0077
C	-4.2197	1.3105	-0.4315	C	-4.4368	-1.9065	-0.4293
C	-5.0557	0.2808	-0.0119	C	-3.13	-1.4372	-0.4275
H	-0.7621	-2.3633	-0.2401	O	3.127	2.4206	-0.1178
H	1.1986	3.0629	0.2715	C	5.2026	-2.1264	0.1282
H	3.4895	-2.0436	-0.1751	H	-1.8579	2.4385	-0.1722
H	5.0503	1.1234	0.0887	H	1.2432	-2.4533	0.1393
H	3.6849	3.1829	0.2697	H	4.5722	0.305	-0.0075
H	-2.7172	-2.0389	0.7832	H	-3.6949	1.678	0.8151
H	-2.1958	1.942	-0.7547	H	-6.0036	0.8387	0.8012
H	-5.1562	-1.7255	0.758	H	-6.4935	-1.4586	0.0085
H	-4.6406	2.2502	-0.7699	H	-4.6443	-2.9128	-0.7744
H	-6.1311	0.4182	-0.0173	H	-2.3273	-2.0758	-0.7747
				H	2.3565	3.0342	-0.1471
				H	5.6157	-3.1312	0.186
				H	5.5365	-1.6445	-0.7941
				H	5.5263	-1.5457	0.9956

Table S4. Cartesian coordinates of optimized geometries (ωB97XD/6-311+G**) of 115 combinatorially-designed flavonoids. Total energy (E) in Hartrees. N_i is the number of imaginary frequencies.

Compound 1			
N_i = 0	E = -803.241795704		
C	4.6749	-0.1022	-0.1472
C	3.8715	-1.1475	0.2724
C	2.4844	-1.0067	0.3097
O	1.7894	-2.0886	0.7582
C	1.8945	0.214	-0.0702
C	2.7317	1.2537	-0.5011
C	4.1057	1.1076	-0.5418
C	0.4441	0.4508	-0.0112
C	-0.144	1.6465	0.2248
C	-1.5771	1.7947	0.2464
O	-2.1336	2.8832	0.4401
C	-2.3342	0.5525	0.0399
C	-3.7357	0.514	0.0602
C	-4.4039	-0.6781	-0.1266
C	-3.6821	-1.8607	-0.3367
C	-2.3012	-1.85	-0.3616
C	-1.644	-0.6379	-0.1729
O	-0.2786	-0.6723	-0.2161
H	5.7506	-0.2343	-0.1741
H	4.3004	-2.0948	0.578
H	0.8428	-1.9663	0.6153
H	2.2843	2.1862	-0.824
H	4.7282	1.9246	-0.8851
H	0.468	2.5167	0.418
H	-4.2791	1.4366	0.2269
H	-5.4868	-0.703	-0.1104
H	-4.2095	-2.7961	-0.4825
H	-1.7235	-2.7518	-0.5238

Compound 2			
N_i = 0	E = -803.243888406		
C	4.4767	0.1655	0.1754
C	3.6921	-0.9153	-0.2172
O	4.235	-2.1181	-0.5749
C	2.3075	-0.8038	-0.2601
C	1.7016	0.4052	0.0812
C	2.4824	1.4937	0.4795
C	3.8633	1.3628	0.5233
C	0.2332	0.526	0.0145
C	-0.4464	1.677	-0.1989
C	-1.8857	1.7066	-0.2463
O	-2.5283	2.7487	-0.4342
C	-2.5371	0.4026	-0.0686
C	-3.931	0.2484	-0.0981
C	-4.4995	-0.9969	0.0663
C	-3.6826	-2.1189	0.2641
C	-2.3081	-1.9943	0.2976
C	-1.7487	-0.7294	0.1299
O	-0.3874	-0.657	0.1775
H	5.5565	0.0648	0.2091
H	5.1954	-2.0712	-0.5267
H	1.7171	-1.6558	-0.5734
H	2.0192	2.4277	0.772
H	4.473	2.2014	0.8386
H	0.0945	2.6005	-0.3547
H	-4.5481	1.1255	-0.2531
H	-5.5764	-1.1115	0.0429
H	-4.1314	-3.097	0.3933
H	-1.6597	-2.8483	0.4505

Table S4. (cont.)

Compound 3				Compound 4			
N_i = 0	E = -803.245724740			N_i = 0	E = -803.236101342		
C	-4.3668	-0.3052	-0.0043	C	-4.6956	-0.7834	0.0063
O	-5.7023	-0.5689	-0.0361	C	-4.255	0.3853	0.6185
C	-3.8623	0.9338	0.3884	C	-2.9031	0.7047	0.6277
C	-2.4942	1.1471	0.393	C	-1.9778	-0.1516	0.0243
C	-1.6108	0.1337	0.0099	C	-2.424	-1.3325	-0.579
C	-2.1327	-1.109	-0.3672	C	-3.7769	-1.6415	-0.5907
C	-3.4988	-1.3288	-0.3798	C	-0.5402	0.1599	0.0219
C	-0.1616	0.3697	-0.0102	C	0.0239	1.3882	-0.1001
C	0.4376	1.5799	-0.137	C	1.4612	1.5642	-0.1276
C	1.8681	1.7198	-0.1418	O	1.9359	2.7026	-0.2534
O	2.4362	2.8177	-0.2398	C	2.2464	0.3475	-0.0168
C	2.6142	0.4588	-0.0347	C	3.6522	0.3543	-0.0282
C	4.0158	0.4116	-0.0432	C	4.3513	-0.8261	0.079
C	4.6748	-0.7955	0.0592	C	3.6599	-2.0431	0.1986
C	3.9424	-1.9852	0.1715	C	2.2818	-2.0767	0.2096
C	2.5616	-1.9662	0.1804	C	1.5824	-0.8734	0.1003
C	1.9106	-0.7388	0.0759	O	0.2302	-0.9509	0.1205
O	0.547	-0.7709	0.0969	H	-5.752	-1.0269	-0.0028
H	-6.2024	0.211	0.2278	H	-4.9652	1.0515	1.0947
H	-4.5417	1.7222	0.6937	H	-2.5674	1.6098	1.1171
H	-2.1168	2.1101	0.716	H	-1.7124	-2.002	-1.0472
H	-1.4668	-1.9079	-0.6689	H	-4.1146	-2.5537	-1.0691
H	-3.9027	-2.2874	-0.6833	O	-0.7241	2.5134	-0.2353
H	-0.1677	2.4686	-0.253	H	5.4345	-0.8206	0.0713
H	4.5665	1.3407	-0.1313	H	4.2147	-2.9703	0.2825
H	5.7577	-0.827	0.0529	H	1.7321	-3.0055	0.2997
H	4.462	-2.9329	0.252	H	4.1679	1.3026	-0.122
H	1.9778	-2.8745	0.265	H	-0.0916	3.2415	-0.3407

Table S4. (cont.)

Compound 5			
N_i = 0	E = -803.248088959		
C	-5.0557	0.2799	-0.0044
C	-4.5072	-0.9231	0.4298
C	-3.1309	-1.102	0.4331
C	-2.2899	-0.0731	-0.0001
C	-2.8439	1.1369	-0.4281
C	-4.2218	1.3076	-0.4331
C	-0.8292	-0.2612	-0.0123
C	-0.193	-1.4507	-0.1315
C	1.2396	-1.5276	-0.1276
O	1.8572	-2.6105	-0.2241
C	1.939	-0.2569	-0.0154
C	3.3506	-0.1669	-0.0101
O	4.1067	-1.281	-0.1039
C	3.9684	1.0691	0.0951
C	3.1913	2.2211	0.1949
C	1.8063	2.1715	0.1928
C	1.2023	0.9271	0.0866
O	-0.1607	0.902	0.0948
H	-6.1311	0.4173	-0.0055
H	-5.1523	-1.7228	0.7748
H	-2.7129	-2.0354	0.7916
H	-2.1994	1.9377	-0.769
H	-4.6443	2.2457	-0.7741
H	-0.7627	-2.3624	-0.2458
H	5.0502	1.1234	0.0989
H	3.6849	3.1825	0.2771
H	1.1987	3.0636	0.2698
H	3.4884	-2.0433	-0.1702

Compound 6			
N_i = 0	E = -803.242690040		
C	-5.1121	-0.5967	0.0131
C	-4.1908	-1.5424	-0.4255
C	-2.834	-1.247	-0.4273
C	-2.3895	0.0063	0.0036
C	-3.3179	0.9516	0.448
C	-4.6725	0.6487	0.4516
C	-0.9515	0.3285	-0.0181
C	-0.433	1.5739	-0.1501
C	0.9862	1.8056	-0.1621
O	1.4834	2.9357	-0.268
C	1.8113	0.5942	-0.0491
C	3.2099	0.6483	-0.0591
C	3.9445	-0.5142	0.0549
C	3.2951	-1.7525	0.1781
C	1.9183	-1.8189	0.187
C	1.1829	-0.6421	0.0719
O	-0.178	-0.7637	0.0935
H	-6.1707	-0.8306	0.0165
H	-4.5288	-2.513	-0.7698
H	-2.1213	-1.9838	-0.7769
H	-2.983	1.917	0.8091
H	-5.3856	1.3842	0.8055
H	-1.0961	2.4207	-0.2645
O	5.3075	-0.4154	0.0429
H	3.8835	-2.6595	0.2649
H	1.4034	-2.7669	0.2815
H	3.7093	1.6049	-0.1546
H	5.7051	-1.2868	0.139

Table S4. (cont.)

Compound 7					Compound 8			
N_i = 0	E = -803.245832575				N_i = 0	E = -803.240618920		
C	4.9635	-0.8984	0.0063		C	4.8247	-0.5244	0.0156
C	4.6466	0.3845	0.4428		C	4.422	0.747	-0.382
C	3.3274	0.8161	0.4444		C	3.0754	1.0822	-0.3897
C	2.3097	-0.0359	0.0059		C	2.1169	0.1439	0.0036
C	2.6315	-1.327	-0.4232		C	2.5247	-1.1356	0.3933
C	3.9535	-1.7517	-0.4257		C	3.8738	-1.4636	0.4014
C	0.9083	0.4211	-0.0133		C	0.6873	0.498	0.0151
C	0.5019	1.7066	-0.1192		C	0.1815	1.7502	0.1122
C	-0.897	2.0594	-0.1325		C	-1.2378	2	0.1141
O	-1.2868	3.2345	-0.213		O	-1.7156	3.1397	0.1836
C	-1.8176	0.9268	-0.0559		C	-2.0843	0.7998	0.0393
C	-3.2128	1.0783	-0.0763		C	-3.4841	0.8563	0.0525
C	-4.0458	-0.0123	0.0019		C	-4.2155	-0.3117	-0.0194
C	-3.4938	-1.301	0.1044		C	-3.5771	-1.5538	-0.1026
O	-4.2777	-2.4046	0.1881		C	-2.1983	-1.63	-0.1136
C	-2.1224	-1.4878	0.1238		O	-1.5845	-2.841	-0.1889
C	-1.3052	-0.368	0.0419		C	-1.4607	-0.4403	-0.0437
O	0.0361	-0.6043	0.0727		O	-0.1089	-0.5844	-0.0678
H	5.9946	-1.233	0.0069		H	5.8774	-0.7833	0.0207
H	5.428	1.0497	0.7914		H	5.158	1.4785	-0.6947
H	3.0912	1.8103	0.805		H	2.7723	2.069	-0.7188
H	1.8504	-1.9938	-0.767		H	1.7894	-1.8692	0.7006
H	4.1942	-2.7518	-0.7676		H	4.1825	-2.455	0.7123
H	1.2342	2.4969	-0.2133		H	0.8515	2.5943	0.203
H	-3.6296	2.0752	-0.1547		H	-3.9727	1.8201	0.1197
H	-5.1235	0.1067	-0.0141		H	-5.2982	-0.2759	-0.0104
H	-5.2068	-2.1508	0.176		H	-4.1517	-2.4712	-0.1561
H	-1.6923	-2.4781	0.2032		H	-0.6267	-2.7287	-0.1671

Table S4. (cont.)

Compound 9				Compound 10			
N_i = 0	E = -878.471944424			N_i = 0	E = -878.467411519		
C	5.0001	-0.5691	-0.0043	C	5.0161	-0.925	0.0235
C	4.491	0.5755	0.6001	C	4.6115	0.246	0.656
C	3.1221	0.8116	0.6124	C	3.2724	0.6157	0.656
C	2.249	-0.1057	0.0208	C	2.3243	-0.1921	0.0224
C	2.7644	-1.2626	-0.5741	C	2.7336	-1.3746	-0.6029
C	4.1337	-1.4876	-0.5896	C	4.0741	-1.7341	-0.6049
C	0.7953	0.1156	0.0235	C	0.8985	0.1707	0.0145
C	0.1535	1.3065	-0.088	C	0.3822	1.4213	-0.1077
C	-1.2888	1.3753	-0.1041	C	-1.0444	1.6559	-0.1364
O	-1.8554	2.4827	-0.221	O	-1.4753	2.8123	-0.2607
C	-1.991	0.1217	0.0035	C	-1.879	0.4706	-0.0282
C	-3.4057	0.043	0.0012	C	-3.2802	0.5452	-0.0414
C	-4.0286	-1.1874	0.1018	C	-4.0259	-0.6074	0.0667
C	-3.2558	-2.3458	0.2029	C	-3.389	-1.8559	0.1862
C	-1.8728	-2.3069	0.2063	C	-2.0157	-1.9418	0.1973
C	-1.2569	-1.0647	0.1053	C	-1.2618	-0.7726	0.0888
O	0.0968	-1.0423	0.1161	O	0.0886	-0.9074	0.1108
H	6.0694	-0.7478	-0.0159	H	6.0626	-1.2082	0.0225
H	5.1608	1.288	1.0678	H	5.3398	0.8739	1.1562
H	2.7333	1.6986	1.0953	H	2.9637	1.5216	1.1614
H	2.0936	-1.9792	-1.0326	H	2.0032	-2.0055	-1.0954
H	4.5247	-2.3817	-1.0613	H	4.384	-2.6473	-1.1
O	0.8251	2.4786	-0.2185	O	1.1782	2.5146	-0.2416
H	-5.1105	-1.2376	0.1004	O	-5.3879	-0.4977	0.0536
H	-3.7565	-3.3038	0.2808	H	-3.9895	-2.7555	0.2683
H	-1.2718	-3.2035	0.2831	H	-1.5134	-2.8968	0.2888
O	-4.155	1.1625	-0.0944	H	-3.7667	1.5085	-0.1347
H	0.1546	3.1718	-0.3166	H	0.5784	3.2695	-0.3485
H	-3.546	1.9262	-0.1643	H	-5.7926	-1.3664	0.1451

Table S4. (cont.)

Compound 11			
N_i = 0	E = -878.470877296		
C	4.8366	-1.2613	0.0111
C	4.5578	-0.0483	0.6317
C	3.2629	0.4546	0.6386
C	2.2311	-0.2592	0.0224
C	2.5143	-1.4844	-0.5914
C	3.8114	-1.9782	-0.5989
C	0.8503	0.2478	0.0182
C	0.4586	1.5413	-0.0831
C	-0.9448	1.9111	-0.1161
O	-1.2552	3.108	-0.2255
C	-1.8827	0.8135	-0.0348
C	-3.2767	0.9996	-0.0583
C	-4.1306	-0.0715	0.0159
C	-3.605	-1.3749	0.1159
C	-2.2401	-1.5935	0.1385
C	-1.3941	-0.492	0.0622
O	-0.0666	-0.752	0.094
H	5.8492	-1.6487	0.0053
H	5.35	0.5087	1.1187
H	3.0536	1.3923	1.1365
H	1.7205	-2.0443	-1.0714
H	4.0223	-2.9243	-1.0843
O	1.3526	2.5571	-0.1988
H	-5.2057	0.0689	-0.0014
O	-4.4143	-2.4595	0.1934
H	-1.8332	-2.5937	0.2156
H	-3.6694	2.0063	-0.1361
H	0.8256	3.3659	-0.2958
H	-5.338	-2.1867	0.1756

Compound 12			
N_i = 0	E = -878.465443388		
C	4.7409	-0.8651	0.0106
C	3.7668	-1.6734	0.5893
C	2.4361	-1.28	0.5765
C	2.0678	-0.0638	-0.0097
C	3.0484	0.7424	-0.5951
C	4.3775	0.3386	-0.584
C	0.6522	0.3323	-0.011
C	0.1497	1.59	0.0837
C	-1.278	1.8432	0.1045
O	-1.6886	3.008	0.2005
C	-2.134	0.6705	0.0252
C	-3.5361	0.7483	0.0393
C	-4.2792	-0.4087	-0.0363
C	-3.6546	-1.6608	-0.123
C	-2.2793	-1.7569	-0.1337
C	-1.5225	-0.5762	-0.0609
O	-0.1812	-0.7335	-0.0846
H	5.7798	-1.1747	0.0203
H	4.0438	-2.6127	1.0539
H	1.6819	-1.9122	1.03
H	2.774	1.6739	-1.0722
H	5.1304	0.9666	-1.0462
O	0.9537	2.6777	0.1924
H	-5.3614	-0.3608	-0.0277
O	-1.68	-2.9745	-0.2122
H	-4.0112	1.7187	0.1095
H	0.3605	3.441	0.2766
H	-4.2421	-2.5698	-0.1794
H	-0.7209	-2.8738	-0.1868

Table S4. (cont.)

Compound 13			
N_i = 0	E = -878.468497178		
C	4.4701	-1.1865	-0.1012
C	4.1434	0.0256	0.4812
C	2.8251	0.4816	0.4863
C	1.8146	-0.301	-0.0998
C	2.1708	-1.5247	-0.6882
C	3.4807	-1.9669	-0.6973
C	0.389	0.0628	-0.085
C	-0.1518	1.2883	-0.2891
C	-1.5783	1.5222	-0.2581
O	-2.0175	2.6675	-0.427
C	-2.3934	0.3381	-0.0439
C	-3.7976	0.3864	-0.0148
C	-4.5262	-0.7647	0.1806
C	-3.866	-1.9922	0.3522
C	-2.4894	-2.0659	0.328
C	-1.7601	-0.8928	0.1289
O	-0.4088	-1.012	0.1218
H	5.5009	-1.5221	-0.0949
H	4.9016	0.6441	0.9471
O	2.5915	1.66	1.1331
H	1.3956	-2.1273	-1.1461
H	3.7307	-2.912	-1.1639
O	0.6422	2.3661	-0.5454
H	-5.6085	-0.7274	0.2026
H	-4.2888	1.3425	-0.151
H	0.0601	3.1413	-0.5921
H	-4.4434	-2.8963	0.5061
H	-1.964	-3.0037	0.4599
H	1.8722	2.1459	0.6928

Compound 14			
N_i = 0	E = -878.468776164		
C	4.3163	-1.116	-0.2125
C	3.9672	0.1093	0.3477
C	2.6362	0.5061	0.4116
C	1.6417	-0.332	-0.0921
C	1.9842	-1.5693	-0.6487
C	3.3178	-1.9469	-0.7068
C	0.2262	0.0678	-0.0448
C	-0.2703	1.3229	-0.1879
C	-1.6963	1.5811	-0.1734
O	-2.1089	2.7403	-0.3223
C	-2.5455	0.4158	0.001
C	-3.9483	0.5033	0.0302
C	-4.7096	-0.6311	0.1944
C	-4.0849	-1.8816	0.3339
C	-2.7113	-1.994	0.3079
C	-1.9483	-0.8369	0.1394
O	-0.6029	-0.9915	0.1218
H	5.3591	-1.4118	-0.2587
O	4.8992	0.9717	0.857
H	1.216	-2.2219	-1.0432
H	3.588	-2.9005	-1.1457
O	0.5344	2.3968	-0.3911
H	-5.7904	-0.5634	0.217
H	-4.4123	1.4763	-0.0792
H	-0.0582	3.1561	-0.5071
H	-4.6885	-2.7723	0.4634
H	-2.2128	-2.9496	0.4141
H	2.3917	1.4583	0.8632
H	5.7813	0.6001	0.754

Table S4. (cont.)

Compound 15					Compound 16			
N_i = 0	E = -878.470418532				N_i = 0	E = -878.476247490		
C	4.3539	-0.5043	0.0036		C	5.3749	-0.4183	0.0099
C	3.4759	-1.4329	-0.5531		C	4.5087	-1.4124	-0.4339
C	2.1152	-1.184	-0.5379		C	3.1375	-1.1941	-0.4357
C	1.6069	-0.0044	0.0209		C	2.6232	0.0304	0.0003
C	2.4999	0.9133	0.5816		C	3.4964	1.0253	0.4485
C	3.8639	0.6669	0.5768		C	4.8658	0.7989	0.4523
C	0.1598	0.235	0.0178		C	1.1699	0.2702	-0.0175
C	-0.4697	1.4339	-0.0975		C	0.5814	1.4863	-0.1371
C	-1.9115	1.5367	-0.1214		C	-0.8447	1.6222	-0.1377
O	-2.4465	2.6495	-0.2453		O	-1.4237	2.7261	-0.2355
C	-2.635	0.2813	-0.0126		C	-1.5941	0.3786	-0.0273
C	-4.0391	0.2174	-0.0183		C	-3.0004	0.3599	-0.0224
C	-4.6774	-0.9977	0.0835		O	-3.732	1.4934	-0.1095
C	-3.9252	-2.179	0.1911		C	-3.6753	-0.8521	0.0786
C	-2.547	-2.1427	0.1964		C	-2.9569	-2.0391	0.1773
C	-1.9099	-0.9049	0.0937		C	-1.571	-2.0447	0.177
O	-0.5552	-0.9132	0.1106		C	-0.906	-0.8337	0.0737
O	5.6851	-0.7938	-0.0367		O	0.46	-0.8649	0.0849
H	2.1319	1.8208	1.0414		H	6.4449	-0.5929	0.0139
H	4.5507	1.3798	1.0204		H	4.9009	-2.3612	-0.7814
O	0.222	2.597	-0.2291		H	2.4678	-1.9685	-0.7891
H	-5.7595	-1.0467	0.0808		H	3.1078	1.9692	0.8127
H	-4.6023	1.1392	-0.1027		H	5.536	1.5722	0.8094
H	-0.4451	3.2938	-0.3311		H	1.1897	2.3732	-0.2481
H	-4.4322	-3.1334	0.2709		O	-5.042	-0.8986	0.0866
H	-1.951	-3.0434	0.277		H	-3.5049	-2.9709	0.2557
H	1.44	-1.9092	-0.9758		H	-1.0111	-2.9675	0.2544
H	3.8682	-2.3407	-0.9961		H	-3.0933	2.2392	-0.1764
H	6.1915	-0.0839	0.3723		H	-5.3934	-0.0048	0.0002

Table S4. (cont.)

Compound 17			
N_i = 0	E = -878.483254598		
C	-5.2499	-0.653	-0.0027
C	-4.3135	-1.5597	-0.489
C	-2.9625	-1.2395	-0.4822
C	-2.5398	0.001	0.0041
C	-3.483	0.9073	0.496
C	-4.8316	0.5787	0.4918
C	-1.1096	0.3523	-0.0101
C	-0.6117	1.6057	-0.1087
C	0.8067	1.8391	-0.1163
O	1.2983	2.9879	-0.1978
C	1.637	0.6573	-0.0352
C	3.0517	0.7178	-0.0426
O	3.6781	1.9076	-0.1256
C	3.8068	-0.4372	0.0355
C	3.1588	-1.6725	0.1238
C	1.7724	-1.7772	0.1348
C	1.0417	-0.6067	0.0533
O	-0.314	-0.7342	0.0713
H	-6.3038	-0.9068	-0.0056
H	-4.6352	-2.5194	-0.8767
H	-2.2379	-1.9459	-0.8683
H	-3.164	1.8608	0.9002
H	-5.5564	1.2834	0.8828
H	-1.2802	2.4507	-0.2005
O	3.8606	-2.8285	0.2022
H	1.2806	-2.7382	0.2043
H	2.9768	2.5982	-0.171
H	4.8893	-0.3806	0.0276
H	4.8061	-2.6451	0.1916

Compound 18			
N_i = 0	E = -878.474239485		
C	5.1073	0.2298	0.0134
C	4.2409	1.2332	0.4355
C	2.8692	1.0188	0.4311
C	2.3546	-0.211	0.0098
C	3.2279	-1.2148	-0.4183
C	4.5977	-0.9923	-0.415
C	0.9014	-0.4442	0.0209
C	0.2932	-1.6506	0.1123
C	-1.1385	-1.7682	0.1046
O	-1.7233	-2.869	0.1709
C	-1.8788	-0.5157	0.0245
C	-3.2879	-0.4601	0.0205
O	-4.0294	-1.5946	0.0819
C	-3.9229	0.768	-0.0521
C	-3.1772	1.9429	-0.1208
C	-1.794	1.9194	-0.1197
C	-1.1613	0.6805	-0.0461
O	0.1981	0.7009	-0.0568
H	6.1778	0.4011	0.0148
H	4.6334	2.186	0.7711
H	2.1995	1.8015	0.766
H	2.8398	-2.1628	-0.772
H	5.2682	-1.7727	-0.7556
H	0.8853	-2.5506	0.2025
O	-1.0907	3.0897	-0.1875
H	-3.4004	-2.3461	0.1241
H	-5.0052	0.808	-0.0559
H	-3.6808	2.901	-0.1779
H	-0.1446	2.9049	-0.1891

Table S4. (cont.)

Compound 19			
N_i = 0	E = -878.478627286		
C	-4.9448	-0.1132	-0.122
C	-4.207	0.9774	0.3025
C	-2.8134	0.9263	0.3258
C	-2.15	-0.2503	-0.0725
C	-2.9224	-1.338	-0.5071
C	-4.3029	-1.2797	-0.5353
C	-0.6879	-0.3936	-0.0288
C	-0.0193	-1.5513	0.1833
C	1.4159	-1.5881	0.1931
O	2.0593	-2.6436	0.3735
C	2.0854	-0.3107	0
C	3.4933	-0.1823	0.0133
O	4.2774	-1.2637	0.2018
C	4.0794	1.0615	-0.1651
C	3.275	2.1823	-0.3542
C	1.8912	2.0949	-0.3707
C	1.3229	0.8439	-0.193
O	-0.0409	0.7763	-0.2223
H	-6.027	-0.0511	-0.1378
H	-4.6935	1.8913	0.6229
O	-2.1862	2.0453	0.7816
H	-2.4195	-2.237	-0.8432
H	-4.8748	-2.1314	-0.8821
H	-0.5624	-2.4674	0.3685
H	3.6798	-2.0367	0.3141
H	5.1592	1.1451	-0.1539
H	3.7442	3.1495	-0.4925
H	1.2594	2.9612	-0.5177
H	-1.2349	1.9877	0.6289

Compound 20			
N_i = 0	E = -878.480741177		
C	-4.7521	-0.3158	0.1513
C	-4.0189	0.8016	-0.2392
C	-2.6304	0.7583	-0.2712
C	-1.9687	-0.417	0.084
C	-2.6972	-1.5405	0.4838
C	-4.0833	-1.4785	0.5137
C	-0.4963	-0.4658	0.0322
C	0.2418	-1.5842	-0.1606
C	1.6756	-1.5272	-0.1909
O	2.3867	-2.5411	-0.3585
C	2.2582	-0.2048	-0.0273
C	3.6549	0.0153	-0.0505
O	4.5081	-1.0155	-0.2261
C	4.1595	1.2967	0.1052
C	3.2835	2.3653	0.283
C	1.9092	2.1875	0.3091
C	1.4191	0.8994	0.1519
O	0.0647	0.7475	0.1871
H	-5.8359	-0.2683	0.175
O	-4.6169	1.9754	-0.6044
H	-2.1902	-2.4474	0.7889
H	-4.6533	-2.3443	0.8298
H	-0.244	-2.5383	-0.3112
H	3.9632	-1.829	-0.3208
H	5.2314	1.4509	0.0861
H	3.6886	3.3634	0.4036
H	1.2243	3.0139	0.4471
H	-2.0801	1.6365	-0.5849
H	-5.5742	1.8892	-0.5462

Table S4. (cont.)

Compound 21			
N_i = 0	E = -878.482737034		
C	-4.6637	0.178	0.0111
C	-3.845	1.2417	0.3859
C	-2.4703	1.0867	0.3706
C	-1.8911	-0.13	-0.0083
C	-2.726	-1.1848	-0.3894
C	-4.1023	-1.0362	-0.3825
C	-0.4331	-0.2949	0.0057
C	0.2273	-1.4748	0.1187
C	1.6585	-1.5267	0.1145
O	2.2968	-2.5996	0.2044
C	2.3377	-0.2434	0.013
C	3.7471	-0.1294	0.0123
O	4.5213	-1.2318	0.1005
C	4.3443	1.1178	-0.0823
C	3.548	2.2571	-0.1754
C	2.1639	2.1835	-0.1766
C	1.5811	0.928	-0.0814
O	0.2187	0.8794	-0.093
O	-6.0098	0.3767	0.0432
H	-2.3048	-2.129	-0.7138
H	-4.745	-1.8553	-0.6866
H	-0.324	-2.398	0.2295
H	3.9137	-2.0038	0.1596
H	5.425	1.1903	-0.083
H	4.0251	3.2274	-0.2496
H	1.5413	3.0658	-0.2483
H	-1.8422	1.9161	0.6707
H	-4.2935	2.1799	0.6901
H	-6.4698	-0.4262	-0.225

Compound 22			
N_i = 0	E = -878.475045845		
C	5.3071	-0.8686	0.0111
C	4.9778	0.4135	0.4404
C	3.6545	0.8325	0.4389
C	2.6451	-0.0311	0.004
C	2.9795	-1.3211	-0.4184
C	4.3055	-1.7336	-0.4172
C	1.2392	0.4126	-0.0167
C	0.8215	1.6961	-0.1209
C	-0.578	2.0392	-0.1329
O	-0.9788	3.2115	-0.2124
C	-1.4905	0.9001	-0.0552
C	-2.8871	1.0561	-0.0708
C	-3.7076	-0.0399	0.0081
C	-3.1514	-1.3367	0.1046
C	-1.7846	-1.5106	0.119
C	-0.9678	-0.3864	0.038
O	0.376	-0.6168	0.0664
H	6.3413	-1.1937	0.0143
H	5.7526	1.088	0.786
H	3.4092	1.8263	0.7943
H	2.2051	-1.997	-0.7596
H	4.5559	-2.7333	-0.7533
H	1.5476	2.4923	-0.2132
O	-5.0709	0.0068	0.0035
O	-3.9592	-2.42	0.1854
H	-1.3547	-2.5013	0.1952
H	-3.3145	2.0498	-0.1436
H	-5.3769	0.9189	-0.0321
H	-4.8812	-2.1338	0.1765

Table S4. (cont.)

Compound 23			
N_i = 0	E = -878.472305433		
C	-5.1642	-0.576	-0.0106
C	-4.2031	-1.5015	-0.4042
C	-2.8582	-1.1568	-0.399
C	-2.4647	0.1256	-0.0042
C	-3.4333	1.0501	0.3966
C	-4.7759	0.6983	0.3916
C	-1.039	0.4962	-0.017
C	-0.5489	1.7563	-0.1134
C	0.8648	2.0269	-0.1177
O	1.3285	3.1727	-0.1881
C	1.7298	0.8379	-0.0436
C	3.1249	0.9279	-0.0565
C	3.8748	-0.23	0.0189
C	3.2591	-1.4857	0.1018
C	1.883	-1.5812	0.1113
C	1.1206	-0.4073	0.0399
O	-0.2307	-0.5732	0.0643
H	-6.2136	-0.8479	-0.0135
H	-4.5005	-2.4952	-0.7188
H	-2.1148	-1.8797	-0.7126
H	-3.1414	2.0389	0.7297
H	-5.5197	1.4192	0.7104
H	-1.2307	2.5911	-0.2026
O	5.2351	-0.1109	0.0083
O	1.2988	-2.8039	0.1873
H	3.6073	1.8946	-0.123
H	5.6473	-0.9787	0.0714
H	3.8558	-2.3897	0.1556
H	0.3386	-2.7138	0.1651

Compound 24			
N_i = 0	E = -878.473365707		
C	5.0262	-0.22	-0.1545
C	4.4943	1.0046	-0.5553
C	3.1255	1.1938	-0.5139
C	2.2571	0.183	-0.0764
C	2.8093	-1.0534	0.3095
C	4.1912	-1.2377	0.2717
C	0.8147	0.4644	-0.0142
C	0.2659	1.6801	0.2231
C	-1.1595	1.8765	0.2538
O	-1.6805	2.9822	0.4501
C	-1.9598	0.659	0.0532
C	-3.3583	0.6796	0.0847
C	-4.0677	-0.4903	-0.0994
C	-3.3929	-1.7013	-0.3163
C	-2.0147	-1.7345	-0.3489
C	-1.3082	-0.5505	-0.1635
O	0.0572	-0.6327	-0.2146
H	6.0973	-0.3853	-0.1813
H	5.1417	1.7998	-0.9039
H	2.707	2.1382	-0.841
O	2.0808	-2.1104	0.7643
H	4.591	-2.1959	0.5823
H	0.9074	2.5299	0.4113
O	-5.4317	-0.4253	-0.0606
H	-3.8773	1.6151	0.2552
H	-5.8095	-1.2993	-0.2024
H	-3.9621	-2.6133	-0.4599
H	-1.4786	-2.6605	-0.5163
H	1.1385	-1.959	0.6206

Table S4. (cont.)

Compound 25			
N_i = 0	E = -878.475412727		
C	4.8451	0.006	0.1608
C	4.2926	1.2361	0.4967
C	2.9194	1.4337	0.4615
C	2.0831	0.3795	0.084
C	2.6275	-0.8614	-0.2458
C	4.0052	-1.0398	-0.2115
C	0.6213	0.5681	0.0239
C	-0.0051	1.751	-0.1876
C	-1.4396	1.8503	-0.2353
O	-2.0341	2.9222	-0.4163
C	-2.1535	0.5766	-0.0668
C	-3.5507	0.5003	-0.1093
C	-4.177	-0.7192	0.0466
C	-3.4202	-1.8834	0.2495
C	-2.0441	-1.8213	0.2935
C	-1.4174	-0.5881	0.1325
O	-0.0524	-0.5818	0.1869
H	5.9189	-0.1472	0.1877
H	4.9446	2.0486	0.7957
H	2.5049	2.3933	0.7441
O	4.4843	-2.2725	-0.5582
H	0.5795	2.6474	-0.3429
O	-5.542	-0.756	-0.0064
H	-4.1327	1.4002	-0.2674
H	-5.8496	-1.6597	0.1189
H	-3.9265	-2.8351	0.3721
H	-1.447	-2.7113	0.4492
H	1.9948	-1.688	-0.5439
H	5.446	-2.2724	-0.5189

Compound 26			
N_i = 0	E = -878.477203098		
C	-4.7283	-0.3981	-0.0024
C	-4.2625	0.8591	-0.3857
C	-2.9016	1.116	-0.3832
C	-1.9877	0.1299	-0.0009
C	-2.4714	-1.129	0.3736
C	-3.8294	-1.3927	0.3786
C	-0.5454	0.4065	0.0166
C	0.0199	1.6352	0.1378
C	1.444	1.8177	0.1419
O	1.982	2.9313	0.2355
C	2.2282	0.5779	0.0394
C	3.6277	0.5838	0.0494
C	4.3212	-0.6052	-0.0497
C	3.6299	-1.8214	-0.1598
C	2.2513	-1.84	-0.169
C	1.5575	-0.637	-0.0675
O	0.1933	-0.7112	-0.0879
O	-6.0533	-0.7112	0.0151
H	-4.9655	1.626	-0.6923
H	-2.5535	2.0918	-0.701
H	-0.6112	2.5064	0.2495
O	5.6872	-0.5554	-0.0355
H	4.1599	1.5236	0.1347
H	6.0509	-1.4429	-0.1172
H	4.1874	-2.7487	-0.2356
H	1.7036	-2.7706	-0.2519
H	-1.7813	-1.9066	0.6766
H	-4.204	-2.3646	0.6775
H	-6.58	0.0418	-0.2734

Table S4. (cont.)

Compound 27			
N_i = 0	E = -878.471656674		
C	5.0069	-0.942	-0.0048
C	4.7622	0.3801	0.354
C	3.4657	0.8751	0.3581
C	2.3977	0.048	-0.0026
C	2.6476	-1.2822	-0.3544
C	3.9472	-1.7712	-0.3567
C	1.0183	0.5661	-0.0163
C	0.6546	1.8662	-0.0996
C	-0.731	2.2738	-0.0991
O	-1.0725	3.4639	-0.1576
C	-1.701	1.1794	-0.0385
C	-3.0877	1.3789	-0.0495
C	-3.9483	0.3046	-0.0006
C	-3.4466	-1.0003	0.058
O	-4.3233	-2.0359	0.1023
C	-2.0761	-1.2271	0.0693
C	-1.2213	-0.1278	0.025
O	0.1039	-0.4232	0.0529
H	6.021	-1.3255	-0.0056
H	5.5832	1.0275	0.6395
H	3.2898	1.9008	0.6596
H	1.8277	-1.9318	-0.6353
H	4.1314	-2.8021	-0.6361
H	1.4128	2.6327	-0.1819
H	-3.4752	2.3888	-0.0988
H	-5.0223	0.4461	-0.0083
H	-3.8456	-2.8736	0.1335
O	-1.6311	-2.5122	0.1275
H	-0.6679	-2.5348	0.0788

Compound 28			
N_i = 0	E = -878.476559801		
C	-4.9107	-0.5252	-0.1894
C	-3.9947	-1.4425	0.2948
C	-2.6372	-1.1284	0.3491
C	-2.1935	0.1372	-0.0795
C	-3.1416	1.0439	-0.5753
C	-4.486	0.7262	-0.6327
C	-0.7843	0.5527	-0.0064
C	-0.3467	1.8154	0.1946
C	1.0616	2.1329	0.2322
O	1.4763	3.2894	0.3957
C	1.9588	0.9881	0.0797
C	3.3566	1.1066	0.1128
C	4.1654	0.004	-0.0322
C	3.5856	-1.2633	-0.2134
O	4.3446	-2.3759	-0.3648
C	2.2099	-1.4181	-0.2476
C	1.4216	-0.286	-0.1021
O	0.0725	-0.4844	-0.161
H	-5.9611	-0.7904	-0.2281
H	-4.3109	-2.4203	0.6391
O	-1.8229	-2.0924	0.8622
H	-2.8044	2.0089	-0.9352
H	-5.1967	1.4421	-1.0269
H	-1.0603	2.6137	0.346
H	3.7947	2.0873	0.2543
H	5.2454	0.0983	-0.0098
H	5.279	-2.1435	-0.3371
H	1.7561	-2.3908	-0.3895
H	-0.8964	-1.8599	0.7236

Table S4. (cont.)

Compound 29			
N_i = 0	E = -878.478733555		
C	-4.7477	-0.1953	0.178
C	-3.838	-1.1736	-0.2132
C	-2.4773	-0.8956	-0.2589
C	-2.0207	0.3772	0.0831
C	-2.9272	1.3631	0.4827
C	-4.2823	1.0671	0.5259
C	-0.5778	0.6745	0.0153
C	-0.0393	1.9021	-0.1605
C	1.389	2.1005	-0.213
O	1.9006	3.2211	-0.3576
C	2.1841	0.8804	-0.0926
C	3.5867	0.8806	-0.1387
C	4.3008	-0.2884	-0.0226
C	3.6161	-1.5053	0.143
O	4.2764	-2.6843	0.2606
C	2.2331	-1.5423	0.1927
C	1.5384	-0.346	0.0726
O	0.1804	-0.4344	0.1338
H	-5.8073	-0.4263	0.2123
O	-4.2328	-2.4337	-0.5672
H	-2.5793	2.3457	0.7759
H	-4.9884	1.8262	0.8418
H	-0.6858	2.761	-0.2786
H	4.1038	1.8239	-0.2685
H	5.3846	-0.2862	-0.0584
H	5.2276	-2.5398	0.2137
H	1.7012	-2.4765	0.3208
H	-1.7891	-1.6705	-0.5723
H	-5.191	-2.5027	-0.5052

Compound 30			
N_i = 0	E = -878.480009032		
C	-4.6125	-0.5882	0
C	-4.2653	0.7623	0
C	-2.931	1.1289	0
C	-1.9186	0.1624	0
C	-2.2878	-1.1886	0
C	-3.6196	-1.5646	0
C	-0.4987	0.5466	0
C	-0.0076	1.8104	0
C	1.4073	2.0766	0
O	1.8684	3.23	0
C	2.2608	0.8905	0
C	3.6623	0.9583	0
C	4.4278	-0.184	0
C	3.7972	-1.4402	0
O	4.5107	-2.5939	0
C	2.4168	-1.5438	0
C	1.6699	-0.3732	0
O	0.3176	-0.5282	0
O	-5.9087	-1.0033	0
H	-1.5276	-1.9588	0
H	-3.8988	-2.6116	0
H	-0.6783	2.6579	0
H	4.1381	1.9317	0
H	5.511	-0.1303	0
H	5.4536	-2.3979	0
H	1.9266	-2.5091	0
H	-2.6924	2.185	0
H	-5.0428	1.5188	0
H	-6.4997	-0.2425	0

Table S4. (cont.)

Compound 31			
$N_i = 0$	E = -878.470935916		
C	-4.7621	-0.1549	-0.184
C	-3.9301	-1.1163	0.3624
C	-2.5528	-0.9089	0.4246
C	-2.0028	0.2896	-0.0651
C	-2.8669	1.2451	-0.6189
C	-4.2321	1.0345	-0.6812
C	-0.5656	0.5943	0.0032
C	-0.0391	1.8234	0.2038
C	1.3849	2.0465	0.2341
O	1.8806	3.1673	0.3988
C	2.2127	0.8412	0.0699
C	3.6119	0.8821	0.0967
C	4.3325	-0.2842	-0.0621
C	3.6811	-1.5059	-0.2522
C	2.3007	-1.5683	-0.2821
O	1.6906	-2.7682	-0.4709
C	1.5752	-0.3811	-0.1146
O	0.2161	-0.4974	-0.1595
H	-5.83	-0.3364	-0.2259
H	-4.3293	-2.0446	0.754
O	-1.8278	-1.9031	1.0081
H	-2.4461	2.1595	-1.0204
H	-4.8778	1.785	-1.1202
H	-0.6973	2.667	0.3607
H	4.1081	1.8334	0.2425
H	5.4155	-0.2613	-0.0432
H	4.2454	-2.422	-0.3819
H	0.734	-2.6621	-0.514
H	-0.8834	-1.7304	0.9288

Compound 32			
$N_i = 0$	E = -878.473366613		
C	-4.5684	0.16	0.1701
C	-3.7381	-0.8743	-0.2535
C	-2.3599	-0.7011	-0.2944
C	-1.8074	0.5245	0.0771
C	-2.6336	1.5676	0.5036
C	-4.0071	1.374	0.547
C	-0.3468	0.7089	0.011
C	0.2927	1.8889	-0.1624
C	1.7311	1.9746	-0.2141
O	2.3312	3.0465	-0.3632
C	2.4385	0.6914	-0.0883
C	3.8352	0.5875	-0.1286
C	4.4314	-0.651	-0.0066
C	3.6592	-1.8065	0.1551
C	2.2814	-1.7245	0.1957
O	1.5367	-2.8509	0.354
C	1.6815	-0.464	0.0719
O	0.3229	-0.4527	0.1279
H	-5.6426	0.0108	0.2028
O	-4.2272	-2.0892	-0.6453
H	-2.2109	2.5136	0.8181
H	-4.6524	2.1764	0.8852
H	-0.2822	2.7971	-0.2813
H	4.427	1.4854	-0.2552
H	5.5108	-0.7395	-0.0357
H	4.1279	-2.7789	0.2516
H	0.5979	-2.6292	0.3617
H	-1.7334	-1.5182	-0.6305
H	-5.189	-2.0833	-0.6054

Table S4. (cont.)

Compound 33			
N_i = 0	E = -878.475166208		
C	-4.4576	-0.303	-0.0127
C	-4.0164	0.9711	0.3437
C	-2.6605	1.2513	0.3466
C	-1.7264	0.2721	-0.0046
C	-2.1857	-1.0037	-0.3533
C	-3.5387	-1.2908	-0.3623
C	-0.2892	0.5693	-0.0152
C	0.2744	1.7998	-0.1044
C	1.701	1.987	-0.1034
O	2.2301	3.1055	-0.1672
C	2.4963	0.7512	-0.0346
C	3.8971	0.747	-0.0456
C	4.5772	-0.4523	0.0186
C	3.8853	-1.6659	0.0936
C	2.5044	-1.6818	0.1039
O	1.8381	-2.8653	0.1736
C	1.8196	-0.4608	0.0393
O	0.4629	-0.546	0.0608
O	-5.7763	-0.6388	-0.0325
H	-1.4805	-1.7777	-0.6302
H	-3.8938	-2.2762	-0.6398
H	-0.3557	2.6745	-0.1914
H	4.4269	1.6896	-0.1053
H	5.6606	-0.4637	0.0108
H	4.4195	-2.6076	0.1431
H	0.8862	-2.7098	0.1714
H	-2.3338	2.2408	0.6431
H	-4.7352	1.733	0.6249
H	-6.3174	0.1106	0.238

Compound 34			
N_i = 0	E = -878.471785150		
C	-4.382	0.4438	0.2728
C	-3.6732	-0.6721	-0.1321
C	-2.274	-0.6417	-0.2045
O	-1.7026	-1.7971	-0.648
C	-1.5877	0.5348	0.1256
C	-2.3215	1.6551	0.543
C	-3.7014	1.6098	0.614
C	-0.1225	0.6287	0.0315
C	0.5732	1.7487	-0.2687
C	2.0142	1.7532	-0.3193
O	2.6697	2.772	-0.571
C	2.6497	0.4522	-0.0691
C	4.0398	0.2725	-0.1123
C	4.5902	-0.9717	0.1145
C	3.7595	-2.0662	0.3896
C	2.3876	-1.916	0.4401
C	1.8502	-0.653	0.2106
O	0.4896	-0.5497	0.2796
H	-5.4632	0.3885	0.3259
O	-4.3483	-1.8117	-0.465
H	-0.7542	-1.7987	-0.4675
H	-1.7948	2.5572	0.8289
H	-4.2557	2.4795	0.9448
H	0.0445	2.6658	-0.4894
H	4.6687	1.1277	-0.3294
H	5.6646	-1.1061	0.0794
H	4.1943	-3.0433	0.5651
H	1.7261	-2.7472	0.6519
H	-3.711	-2.5009	-0.6885

Table S4. (cont.)

Compound 35			
N_i = 0	E = -878.476712717		
C	4.3068	-0.0455	-0.0777
C	3.5055	-1.1211	0.2718
C	2.1214	-0.9905	0.2791
O	1.4328	-2.1002	0.6559
C	1.5188	0.2416	-0.0556
C	2.3633	1.3014	-0.4193
C	3.7362	1.1784	-0.4368
C	0.0717	0.4681	-0.0092
C	-0.5338	1.6628	0.2029
C	-1.9657	1.7978	0.2113
O	-2.5354	2.8853	0.3793
C	-2.712	0.5448	0.0283
C	-4.1128	0.4922	0.0487
C	-4.7687	-0.7108	-0.1124
C	-4.0347	-1.8901	-0.2959
C	-2.6538	-1.8654	-0.3206
C	-2.0099	-0.6425	-0.1582
O	-0.6442	-0.6629	-0.2005
O	5.6525	-0.238	-0.0648
H	0.487	-1.9745	0.5064
H	1.9234	2.2469	-0.7124
H	4.3657	2.0109	-0.7293
H	0.0652	2.5433	0.3897
H	-4.6659	1.4123	0.1956
H	-5.8513	-0.7465	-0.0957
H	-4.5522	-2.8341	-0.4208
H	-2.0662	-2.7644	-0.4616
H	3.9506	-2.0711	0.5419
H	6.1046	0.5761	-0.3115

Compound 36			
N_i = 0	E = -878.471699745		
C	-4.3355	-0.6227	0.0414
C	-3.4221	-1.6067	0.3745
C	-2.0529	-1.3524	0.3655
O	-1.246	-2.3934	0.7355
C	-1.5946	-0.0672	0.026
C	-2.5296	0.9166	-0.3232
C	-3.8849	0.6463	-0.3162
C	-0.1703	0.3029	0.0538
C	0.3125	1.5354	0.3331
C	1.727	1.812	0.3323
O	2.1877	2.9354	0.5711
C	2.5871	0.6557	0.0461
C	3.9863	0.7444	0.033
C	4.7542	-0.3722	-0.2236
C	4.1367	-1.6053	-0.4728
C	2.7602	-1.7193	-0.4681
C	2.0016	-0.5816	-0.2087
O	0.6443	-0.7381	-0.2234
H	-0.3274	-2.1997	0.5133
H	-2.1925	1.9024	-0.6203
O	-4.7414	1.6568	-0.6757
H	-0.37	2.3369	0.5799
H	4.4488	1.7043	0.2297
H	5.8349	-0.2988	-0.2322
H	4.7423	-2.4814	-0.6728
H	2.2619	-2.6619	-0.6589
H	-3.7624	-2.5983	0.6487
H	-5.3983	-0.8407	0.0476
H	-5.6486	1.3363	-0.6521

Table S4. (cont.)

Compound 37			
N_i = 0	E = -878.471241770		
C	4.5025	-0.5545	-0.0392
C	3.6258	-1.495	0.4773
C	2.2621	-1.2245	0.4965
O	1.4676	-2.1887	1.0414
C	1.7616	0.0018	0.0155
C	2.6787	0.9287	-0.514
C	4.0409	0.6533	-0.5419
C	0.325	0.3143	0.0795
C	-0.2188	1.4753	0.5027
C	-1.6472	1.6721	0.5449
O	-2.1626	2.7322	0.9215
C	-2.4471	0.5136	0.1266
C	-3.8496	0.5271	0.1311
C	-4.5594	-0.5899	-0.2563
C	-3.88	-1.7484	-0.657
C	-2.4997	-1.7884	-0.6694
C	-1.7987	-0.6517	-0.2761
O	-0.4354	-0.7358	-0.3127
H	0.5385	-2.0329	0.8354
O	2.1895	2.0926	-1.0153
H	0.4283	2.2789	0.8246
H	-4.3605	1.4293	0.446
H	-5.6426	-0.575	-0.2509
H	-4.4404	-2.6251	-0.96
H	-1.9545	-2.6733	-0.9742
H	3.9777	-2.4428	0.8653
H	5.5651	-0.768	-0.058
H	4.7236	1.3843	-0.9605
H	2.9141	2.6194	-1.3687

Compound 38			
N_i = 0	E = -878.475193551		
C	4.1157	0.1607	-0.1023
C	3.3189	-0.9491	0.2038
O	3.8867	-2.1592	0.4862
C	1.9429	-0.8261	0.2271
C	1.3399	0.4063	-0.0499
C	2.1394	1.5058	-0.3662
C	3.5207	1.3804	-0.3908
C	-0.1243	0.5308	-0.0023
C	-0.8123	1.6861	0.1739
C	-2.2497	1.7151	0.1979
O	-2.8976	2.7616	0.3478
C	-2.8989	0.4061	0.0474
C	-4.2926	0.2507	0.0644
C	-4.8576	-1.0001	-0.0697
C	-4.0372	-2.1261	-0.2237
C	-2.6623	-1.9999	-0.2446
C	-2.1068	-0.7296	-0.1083
O	-0.7454	-0.6558	-0.1428
O	5.4609	-0.0489	-0.0999
H	4.8464	-2.081	0.4301
H	1.3483	-1.6959	0.477
H	1.6955	2.4618	-0.6135
H	4.1478	2.229	-0.6422
H	-0.2786	2.6157	0.3163
H	-4.9127	1.131	0.1853
H	-5.9347	-1.1156	-0.0558
H	-4.4829	-3.1085	-0.3283
H	-2.0111	-2.8572	-0.363
H	5.9311	0.7729	-0.2777

Table S4. (cont.)

Compound 39			
N_i = 0	E = -878.476571497		
C	4.178	0.352	-0.0156
C	3.2867	1.3751	0.2889
O	3.7187	2.6342	0.5916
C	1.9147	1.1528	0.3
C	1.4348	-0.1236	0.0104
C	2.3105	-1.1629	-0.3013
C	3.6773	-0.9122	-0.311
C	-0.019	-0.3758	0.0466
C	-0.597	-1.5724	0.3014
C	-2.029	-1.7299	0.3214
O	-2.5803	-2.8172	0.5389
C	-2.7892	-0.4981	0.076
C	-4.1916	-0.4685	0.0774
C	-4.8653	0.7118	-0.1546
C	-4.1477	1.8922	-0.3935
C	-2.7672	1.8902	-0.4006
C	-2.1008	0.6897	-0.1639
O	-0.738	0.7373	-0.1867
H	4.6806	2.6654	0.5721
H	1.2441	1.9651	0.5474
H	1.952	-2.1523	-0.5551
O	4.5018	-1.9532	-0.6279
H	0.0214	-2.4339	0.5132
H	-4.731	-1.3895	0.2647
H	-5.9484	0.73	-0.1527
H	-4.6792	2.8189	-0.5761
H	-2.1948	2.791	-0.5847
H	5.2477	0.5377	-0.0279
H	5.4195	-1.6623	-0.6188

Compound 40			
N_i = 0	E = -953.699757227		
C	5.288	-0.7373	0.0084
C	4.3881	-1.6111	-0.5946
C	3.0293	-1.3291	-0.5849
C	2.5589	-0.1596	0.0222
C	3.4653	0.7128	0.6314
C	4.8231	0.4198	0.6246
C	1.1155	0.1224	0.0199
C	0.5294	1.3434	-0.0929
C	-0.9053	1.4817	-0.112
O	-1.4232	2.6131	-0.2277
C	-1.6654	0.261	-0.0074
C	-3.0748	0.2633	-0.0116
C	-3.7621	-0.9382	0.0835
C	-3.0557	-2.1359	0.1843
C	-1.6726	-2.1619	0.1915
C	-0.9881	-0.9578	0.0941
O	0.3683	-1.0011	0.1092
O	1.2572	2.4829	-0.2219
O	-3.7905	1.408	-0.1011
O	-5.1294	-0.976	0.0837
H	6.3491	-0.9599	0.001
H	4.7449	-2.5143	-1.076
H	2.332	-2.0106	-1.0573
H	3.1105	1.6091	1.1232
H	5.5188	1.0977	1.1056
H	-3.617	-3.0602	0.2576
H	-1.1258	-3.0923	0.2698
H	0.6194	3.2058	-0.3235
H	-3.1553	2.1506	-0.1713
H	-5.4767	-0.081	-0.0059

Table S4. (cont.)

Compound 41			
N_i = 0	E = -953.707189124		
C	-5.1419	-1.0033	0.0032
C	-4.1807	-1.7787	-0.6386
C	-2.849	-1.3874	-0.6295
C	-2.468	-0.2068	0.0169
C	-3.4354	0.5671	0.6638
C	-4.7653	0.1658	0.6559
C	-1.0526	0.1919	0.0158
C	-0.5648	1.4525	-0.0755
C	0.861	1.6993	-0.0968
O	1.2825	2.8737	-0.1904
C	1.707	0.545	-0.0206
C	3.1222	0.6356	-0.0335
C	3.8962	-0.5033	0.0415
C	3.2698	-1.7528	0.1316
C	1.8878	-1.8859	0.1449
C	1.1306	-0.7292	0.0667
O	-0.214	-0.8759	0.0854
O	-1.3749	2.5369	-0.1799
O	3.7268	1.8372	-0.1186
O	3.9974	-2.892	0.2088
H	-6.1812	-1.3116	-0.003
H	-4.4684	-2.6903	-1.1496
H	-2.104	-1.9922	-1.1327
H	-3.149	1.4705	1.1864
H	-5.5085	0.7673	1.1667
H	4.9774	-0.4279	0.0294
H	1.416	-2.8568	0.2149
H	-0.7919	3.3071	-0.2635
H	3.0269	2.5221	-0.1658
H	4.9384	-2.6863	0.2003

Compound 42			
N_i = 0	E = -953.698139027		
C	5.0418	0.551	0.0197
C	4.5777	-0.6164	-0.5771
C	3.2188	-0.9042	-0.5911
C	2.3102	-0.0159	-0.0083
C	2.7802	1.1643	0.5791
C	4.1399	1.4407	0.5958
C	0.8665	-0.2898	-0.0128
C	0.2594	-1.5011	0.0747
C	-1.182	-1.6189	0.0877
O	-1.7082	-2.7466	0.1766
C	-1.9313	-0.3888	0.0098
C	-3.3437	-0.3528	0.0144
C	-3.9905	0.8655	-0.0562
C	-3.2559	2.0509	-0.1299
C	-1.8748	2.0458	-0.1353
C	-1.2234	0.8132	-0.0646
O	0.1267	0.8439	-0.0797
O	0.9647	-2.655	0.1784
O	-4.0701	-1.4976	0.0815
O	-1.1842	3.2231	-0.2064
H	6.1036	0.7694	0.0327
H	5.2749	-1.3068	-1.0376
H	2.8657	-1.8086	-1.0688
H	2.0822	1.8594	1.0302
H	4.4959	2.3523	1.0618
H	-5.0732	0.8954	-0.0539
H	-3.772	3.0025	-0.1846
H	0.3136	-3.3687	0.2618
H	-3.4433	-2.246	0.1284
H	-0.2362	3.0485	-0.2068

Table S4. (cont.)

Compound 43			
N_i = 0	E = -953.704142476		
C	4.7926	-0.9768	-0.1208
C	3.8504	-1.8173	-0.7117
C	2.5156	-1.4584	-0.6939
C	2.087	-0.2596	-0.1019
C	3.0502	0.5849	0.4782
C	4.3944	0.2119	0.4651
C	0.6415	0.0127	-0.0771
C	0.0211	1.2022	-0.2677
C	-1.4149	1.3258	-0.2267
O	-1.9486	2.4421	-0.388
C	-2.146	0.1023	-0.0167
C	-3.5613	0.0648	0.021
C	-4.2144	-1.1399	0.2084
C	-3.4704	-2.311	0.3597
C	-2.0865	-2.3114	0.3298
C	-1.4404	-1.0961	0.1411
O	-0.0848	-1.1132	0.1247
O	0.7353	2.3339	-0.5227
O	-4.2824	1.1968	-0.1258
O	2.7488	1.7461	1.1276
H	5.8424	-1.2471	-0.1212
H	4.1565	-2.7442	-1.1812
H	1.7768	-2.1087	-1.1467
H	5.1153	0.8766	0.9267
H	-5.2968	-1.1598	0.2349
H	-3.9939	-3.2488	0.5053
H	-1.5085	-3.2184	0.4482
H	0.108	3.0731	-0.5527
H	-3.657	1.939	-0.2541
H	1.9982	2.1883	0.6937

Compound 44			
N_i = 0	E = -953.704664175		
C	-4.6371	-0.9405	-0.2167
C	-3.6932	-1.8248	-0.7245
C	-2.3392	-1.5281	-0.6691
C	-1.9223	-0.3187	-0.1026
C	-2.862	0.5735	0.4136
C	-4.2142	0.2559	0.3551
C	-0.4857	-0.0056	-0.0542
C	0.0851	1.2178	-0.1917
C	1.5208	1.3758	-0.1623
O	2.0205	2.5121	-0.3022
C	2.2941	0.1739	0.0152
C	3.7102	0.1846	0.0601
C	4.4037	-1.0004	0.2248
C	3.7001	-2.2003	0.3462
C	2.3178	-2.2481	0.3076
C	1.6304	-1.0514	0.1414
O	0.2786	-1.1129	0.1103
O	-0.6481	2.3411	-0.3934
O	4.3915	1.3447	-0.0556
O	-5.0932	1.1625	0.8809
H	-5.6962	-1.1726	-0.2598
H	-4.0224	-2.7557	-1.1715
H	-1.6123	-2.2215	-1.0722
H	-2.5579	1.5044	0.8738
H	5.4861	-0.9832	0.2576
H	4.2554	-3.1223	0.4742
H	1.7709	-3.1771	0.4015
H	-0.0131	3.0659	-0.5001
H	3.7395	2.0663	-0.172
H	-5.9943	0.8353	0.7903

Table S4. (cont.)

Compound 45			
N_i = 0	E = -953.706366441		
C	-4.6447	-0.3762	-0.0035
C	-4.1044	0.7697	0.5756
C	-2.731	0.956	0.5824
C	-1.8793	0.0018	0.0183
C	-2.4383	-1.1512	-0.5478
C	-3.8084	-1.3396	-0.5652
C	-0.4234	0.1745	0.0205
C	0.2607	1.3438	-0.0885
C	1.7017	1.3655	-0.0979
O	2.3077	2.4538	-0.2108
C	2.3629	0.0886	0.0068
C	3.7738	-0.0368	0.0038
C	4.356	-1.2874	0.1004
C	3.5456	-2.4202	0.1972
C	2.1645	-2.3354	0.1996
C	1.5903	-1.073	0.1031
O	0.2378	-1.0053	0.1141
O	-0.374	2.5378	-0.2236
O	4.5589	1.0584	-0.0898
O	-5.9869	-0.6068	-0.0443
H	-4.7598	1.5098	1.0224
H	-2.3239	1.8441	1.0473
H	-1.7949	-1.903	-0.9886
H	-4.2399	-2.227	-1.0129
H	5.4357	-1.3729	0.0989
H	4.0141	-3.3946	0.2726
H	1.5342	-3.2119	0.273
H	0.318	3.2092	-0.321
H	3.9732	1.8407	-0.1587
H	-6.46	0.1202	0.3742

Compound 46			
N_i = 0	E = -953.700078213		
C	5.1674	-1.2334	0.0203
C	4.1519	-1.9567	-0.5984
C	2.8505	-1.4739	-0.5968
C	2.5533	-0.2537	0.0202
C	3.5754	0.4667	0.6449
C	4.8746	-0.0251	0.6437
C	1.1678	0.2409	0.0125
C	0.7656	1.5326	-0.0884
C	-0.6379	1.8933	-0.1192
O	-0.9582	3.0891	-0.227
C	-1.5688	0.7905	-0.0364
C	-2.9643	0.9816	-0.0552
C	-3.8062	-0.095	0.0208
C	-3.2767	-1.4068	0.1155
C	-1.9162	-1.6132	0.1321
C	-1.0705	-0.5074	0.0561
O	0.2594	-0.7627	0.0855
O	1.6534	2.5546	-0.2035
O	-5.168	-0.0236	0.0148
O	-4.1104	-2.4692	0.1919
H	6.1832	-1.6122	0.0191
H	4.3737	-2.8991	-1.0861
H	2.0644	-2.0385	-1.0836
H	3.3551	1.4007	1.145
H	5.6593	0.537	1.1371
H	-3.3681	1.9851	-0.1281
H	-1.5098	-2.6139	0.2064
H	1.1207	3.3598	-0.2994
H	-5.4592	0.8937	-0.0159
H	-5.0252	-2.1605	0.1804

Table S4. (cont.)

Compound 47			
N_i = 0	E = -953.697011491		
C	5.0543	-0.938	0.004
C	4.7103	0.2692	-0.5949
C	3.3887	0.6969	-0.6028
C	2.396	-0.0886	-0.0098
C	2.7447	-1.3082	0.5813
C	4.0681	-1.7257	0.5906
C	0.987	0.3311	-0.0094
C	0.5065	1.599	0.0849
C	-0.9138	1.8798	0.1085
O	-1.3043	3.0519	0.2057
C	-1.7937	0.723	0.0302
C	-3.1903	0.8411	0.0454
C	-3.9571	-0.3021	-0.032
C	-3.3611	-1.5702	-0.1181
C	-1.9894	-1.6926	-0.1285
C	-1.2027	-0.5313	-0.056
O	0.1369	-0.7171	-0.0815
O	1.3334	2.6705	0.1932
O	-5.3157	-0.1654	-0.0207
O	-1.4265	-2.9243	-0.207
H	6.0875	-1.2664	0.0109
H	5.4726	0.881	-1.0634
H	3.1292	1.631	-1.0833
H	1.9808	-1.9241	1.0411
H	4.3299	-2.6677	1.0588
H	-3.6542	1.8166	0.1151
H	-3.9746	-2.4628	-0.1735
H	0.757	3.4461	0.2797
H	-5.7384	-1.0282	-0.0827
H	-0.4649	-2.8514	-0.1814

Compound 48			
N_i = 0	E = -953.699831399		
C	4.773	-1.3035	-0.0733
C	3.767	-2.0472	-0.6882
C	2.4721	-1.5624	-0.6933
C	2.148	-0.3324	-0.1
C	3.1753	0.413	0.5049
C	4.478	-0.0854	0.5137
C	0.7342	0.0761	-0.0972
C	0.2355	1.321	-0.3011
C	-1.1799	1.6054	-0.2779
O	-1.58	2.7659	-0.444
C	-2.0387	0.4493	-0.0729
C	-3.437	0.5582	-0.0514
C	-4.206	-0.569	0.1373
C	-3.5949	-1.8242	0.3057
C	-2.224	-1.9432	0.2866
C	-1.4467	-0.8005	0.0967
O	-0.0983	-0.9699	0.0978
O	1.0715	2.3706	-0.5456
O	-5.5646	-0.4281	0.1547
O	2.9706	1.5937	1.1571
H	5.7923	-1.6723	-0.0558
H	3.9926	-2.9965	-1.1585
H	1.6839	-2.1362	-1.166
H	5.2497	0.5046	0.9942
H	-3.9031	1.5269	-0.1847
H	-4.2133	-2.7032	0.4525
H	-1.7419	-2.9039	0.4175
H	0.521	3.1684	-0.5909
H	-5.9854	-1.2842	0.2853
H	2.2757	2.1065	0.7079

Table S4. (cont.)

Compound 49			
N_i = 0	E = -953.700074784		
C	-4.6472	-1.1755	-0.1968
C	-3.6432	-1.9688	-0.7394
C	-2.3167	-1.5646	-0.6967
C	-1.9887	-0.3388	-0.1076
C	-2.9885	0.4621	0.4434
C	-4.3117	0.0382	0.3962
C	-0.5799	0.0861	-0.0702
C	-0.1095	1.3523	-0.2142
C	1.3083	1.6422	-0.2048
O	1.6978	2.8098	-0.3556
C	2.1844	0.495	-0.0308
C	3.5816	0.6244	-0.0022
C	4.3665	-0.495	0.1637
C	3.7741	-1.763	0.3019
C	2.4057	-1.9026	0.2738
C	1.6117	-0.7673	0.1062
O	0.2679	-0.9546	0.0901
O	-0.9424	2.4068	-0.4129
O	5.7233	-0.3352	0.1909
O	-5.2512	0.8596	0.9567
H	-5.6846	-1.4916	-0.2294
H	-3.9033	-2.9133	-1.2031
H	-1.5428	-2.1875	-1.1269
H	-2.7526	1.4039	0.9212
H	4.0335	1.6029	-0.1098
H	4.4058	-2.6352	0.4318
H	1.9378	-2.8734	0.3803
H	-0.3708	3.1813	-0.5316
H	6.155	-1.1884	0.3049
H	-6.1243	0.4626	0.8735

Compound 50			
N_i = 0	E = -953.701694282		
C	-4.683	-0.6328	0.0089
C	-3.7691	-1.526	-0.5478
C	-2.4198	-1.2213	-0.5369
C	-1.9589	-0.0204	0.0176
C	-2.8878	0.8631	0.575
C	-4.2407	0.5612	0.5738
C	-0.5217	0.274	0.0151
C	0.0612	1.498	-0.0985
C	1.4952	1.6603	-0.1223
O	1.9854	2.7951	-0.2405
C	2.2707	0.4349	-0.0205
C	3.6736	0.4396	-0.034
C	4.3601	-0.7503	0.0632
C	3.6619	-1.9661	0.1733
C	2.2856	-1.9829	0.1848
C	1.5918	-0.7763	0.0863
O	0.2363	-0.8422	0.1078
O	-0.6765	2.6336	-0.2279
O	5.7261	-0.711	0.0472
O	-6.0009	-0.9788	-0.0236
H	-4.1247	-2.4508	-0.9866
H	-1.7168	-1.9199	-0.9742
H	-2.5567	1.7873	1.0295
H	-4.9551	1.2476	1.0155
H	4.2075	1.3783	-0.1198
H	4.2176	-2.8947	0.2474
H	1.7359	-2.9122	0.2673
H	-0.0355	3.3548	-0.3287
H	6.0827	-1.6016	0.1266
H	-6.5339	-0.2918	0.3905

Table S4. (cont.)

Compound 51			
N_i = 0	E = -953.696628343		
C	-4.8583	-1.3446	0.002
C	-3.7848	-2.0269	-0.5628
C	-2.5174	-1.4617	-0.5565
C	-2.3123	-0.1986	0.011
C	-3.3924	0.4797	0.5836
C	-4.6572	-0.0947	0.5776
C	-0.9613	0.3805	0.0077
C	-0.6258	1.6919	-0.0749
C	0.7605	2.129	-0.0991
O	1.0116	3.3399	-0.1902
C	1.756	1.08	-0.0312
C	3.1383	1.3272	-0.0438
C	4.0286	0.2816	0.0121
C	3.5661	-1.0408	0.0773
C	2.206	-1.3126	0.0846
C	1.3134	-0.2408	0.0365
O	0.0055	-0.5719	0.0652
O	-1.5627	2.6686	-0.1754
O	4.4785	-2.0437	0.1354
O	1.7992	-2.6101	0.1466
H	-5.8477	-1.7879	-0.0037
H	-3.9344	-3.0021	-1.0116
H	-1.6861	-1.9968	-0.9998
H	-3.2443	1.4459	1.0473
H	-5.4869	0.4361	1.0302
H	3.4915	2.3493	-0.0974
H	5.098	0.4545	0.0067
H	-1.0719	3.5021	-0.253
H	4.0315	-2.8972	0.1871
H	0.8369	-2.6617	0.0964

Compound 52			
N_i = 0	E = -953.703406058		
C	-4.5621	-1.6255	-0.0869
C	-3.4935	-2.2481	-0.7297
C	-2.254	-1.6352	-0.7344
C	-2.0489	-0.3932	-0.1135
C	-3.1393	0.2295	0.5189
C	-4.3852	-0.3979	0.5271
C	-0.685	0.1583	-0.1104
C	-0.3162	1.4488	-0.2811
C	1.0701	1.8712	-0.2499
O	1.3466	3.0726	-0.3811
C	2.0329	0.8048	-0.0824
C	3.4208	1.0304	-0.061
C	4.3002	-0.0125	0.0843
C	3.8071	-1.3259	0.212
C	2.4484	-1.5826	0.1957
C	1.5765	-0.5097	0.0487
O	0.2546	-0.8086	0.051
O	-1.2504	2.418	-0.4962
O	4.6418	-2.3838	0.3555
O	-3.0465	1.4082	1.2003
H	-5.5383	-2.0966	-0.0694
H	-3.6274	-3.2041	-1.2211
H	-1.4172	-2.1153	-1.2274
H	-5.2067	0.0993	1.0296
H	3.7874	2.0447	-0.1632
H	5.3711	0.1576	0.1005
H	2.0664	-2.5906	0.2958
H	-0.7805	3.2669	-0.5182
H	5.5589	-2.0885	0.352
H	-2.4126	2.0011	0.7595

Table S4. (cont.)

Compound 53			
N_i = 0	E = -953.703612755		
C	4.4682	-1.4364	-0.2105
C	3.3898	-2.1437	-0.7284
C	2.1059	-1.6207	-0.6785
C	1.8955	-0.3612	-0.1058
C	2.9712	0.3531	0.4219
C	4.2503	-0.1888	0.3663
C	0.5324	0.1916	-0.0638
C	0.176	1.495	-0.1717
C	-1.2176	1.905	-0.161
O	-1.4975	3.1084	-0.2779
C	-2.1836	0.8375	-0.0307
C	-3.5716	1.065	-0.011
C	-4.4534	0.0209	0.1062
C	-3.963	-1.2963	0.2069
C	-2.6052	-1.5553	0.1896
C	-1.7305	-0.4803	0.0696
O	-0.411	-0.7783	0.0612
O	1.0927	2.4826	-0.3343
O	-4.8009	-2.3556	0.3245
O	5.2665	0.5526	0.9046
H	5.4722	-1.846	-0.2492
H	3.5575	-3.1154	-1.1785
H	1.2745	-2.1788	-1.0896
H	2.8284	1.3183	0.8889
H	-3.9363	2.0822	-0.09
H	-5.5241	0.1925	0.1217
H	-2.2254	-2.566	0.2671
H	0.5865	3.3047	-0.4305
H	-5.7172	-2.0579	0.3245
H	6.0993	0.0775	0.8167

Compound 54			
N_i = 0	E = -953.705068250		
C	-4.5536	-0.8237	0.0019
C	-3.582	-1.6285	-0.5899
C	-2.258	-1.2271	-0.5736
C	-1.8814	-0.0168	0.022
C	-2.8672	0.7759	0.6165
C	-4.1949	0.3769	0.61
C	-0.4701	0.384	0.0184
C	0.0211	1.6445	-0.082
C	1.4457	1.9069	-0.1139
O	1.8476	3.0777	-0.2199
C	2.2994	0.7419	-0.0365
C	3.7031	0.8225	-0.0633
C	4.4745	-0.3098	0.0117
C	3.8521	-1.5689	0.117
C	2.4747	-1.6845	0.1424
C	1.714	-0.5227	0.063
O	0.3704	-0.6815	0.0947
O	-0.7941	2.7274	-0.1927
O	4.5785	-2.711	0.1954
O	-5.845	-1.2586	-0.0389
H	-3.8728	-2.5601	-1.061
H	-1.5094	-1.8562	-1.0398
H	-2.6001	1.7047	1.1034
H	-4.9554	0.9921	1.0792
H	4.1698	1.7968	-0.145
H	5.557	-0.2501	-0.0101
H	1.9941	-2.6512	0.2222
H	-0.205	3.4924	-0.2878
H	5.5192	-2.5054	0.1757
H	-6.4209	-0.6178	0.3918

Table S4. (cont.)

Compound 55			
N_i = 0	E = -953.697822379		
C	4.4911	-1.2931	-0.1333
C	4.2455	-0.0719	0.4693
C	2.9606	0.4708	0.4831
C	1.9018	-0.232	-0.1188
C	2.1753	-1.468	-0.7261
C	3.4525	-1.9968	-0.7411
C	0.5051	0.2265	-0.1008
C	0.0402	1.4882	-0.2716
C	-1.37	1.8131	-0.23
O	-1.7332	2.9886	-0.3587
C	-2.2667	0.6794	-0.0577
C	-3.664	0.8119	-0.0314
C	-4.4477	-0.3109	0.1198
C	-3.8688	-1.5811	0.2434
C	-2.4981	-1.7311	0.2177
C	-1.7003	-0.5855	0.0698
O	-0.3643	-0.797	0.0697
O	0.903	2.5178	-0.4981
O	-1.9444	-2.9667	0.3341
O	2.8046	1.6479	1.1537
H	5.4974	-1.6962	-0.1333
H	5.043	0.486	0.9462
H	1.3618	-2.0105	-1.1932
H	3.6395	-2.9493	-1.2219
H	-4.1037	1.796	-0.1333
H	-5.527	-0.2206	0.1418
H	-4.4882	-2.4631	0.3582
H	0.3787	3.334	-0.5177
H	-0.9838	-2.9079	0.266
H	2.1169	2.1893	0.7274

Compound 56			
N_i = 0	E = -953.698175129		
C	4.3651	-1.1852	0.2298
C	4.0916	0.0582	-0.3326
C	2.7879	0.5373	-0.3934
C	1.7444	-0.237	0.1133
C	2.0105	-1.4929	0.671
C	3.3182	-1.9522	0.7271
C	0.3558	0.2446	0.0627
C	-0.0764	1.5275	0.167
C	-1.4885	1.8616	0.1465
O	-1.8361	3.0448	0.2592
C	-2.4065	0.7414	0.0109
C	-3.8018	0.8987	-0.0201
C	-4.6054	-0.2127	-0.1454
C	-4.0497	-1.4965	-0.2377
C	-2.6824	-1.6707	-0.2054
C	-1.8638	-0.5362	-0.0825
O	-0.5338	-0.7688	-0.0669
O	0.7844	2.5621	0.3339
O	-2.1508	-2.9189	-0.2905
O	5.0736	0.8582	-0.8495
H	5.3876	-1.5453	0.2736
H	2.6022	1.5006	-0.8487
H	1.2037	-2.0976	1.0651
H	3.5296	-2.9206	1.1657
H	-4.2239	1.8929	0.055
H	-5.6828	-0.1033	-0.172
H	-4.6853	-2.3692	-0.3329
H	0.2364	3.357	0.4283
H	-1.1892	-2.8749	-0.2276
H	5.9294	0.4253	-0.7643

Table S4. (cont.)

Compound 57			
N_i = 0	E = -953.699808922		
C	4.4288	-0.522	0.0084
C	4.001	0.6834	-0.5433
C	2.6512	0.9983	-0.5483
C	1.7108	0.1165	-0.007
C	2.1573	-1.0963	0.5344
C	3.5034	-1.4136	0.5485
C	0.2776	0.4229	-0.0083
C	-0.3073	1.6469	0.0811
C	-1.7453	1.8093	0.099
O	-2.2308	2.9465	0.1926
C	-2.5268	0.585	0.0216
C	-3.9309	0.5748	0.0317
C	-4.5998	-0.6273	-0.0389
C	-3.8972	-1.8378	-0.1181
C	-2.5183	-1.847	-0.1265
C	-1.8378	-0.6208	-0.0566
O	-0.489	-0.6927	-0.0777
O	0.4266	2.7853	0.1863
O	-1.8435	-3.0249	-0.1997
O	5.7429	-0.8803	0.0436
H	4.7243	1.3687	-0.9722
H	2.3319	1.9312	-0.9931
H	1.4458	-1.795	0.9579
H	3.8479	-2.3476	0.9767
H	-4.4656	1.5143	0.0949
H	-5.6829	-0.6477	-0.0332
H	-4.4263	-2.7821	-0.1723
H	-0.2155	3.508	0.2699
H	-0.8927	-2.8628	-0.1956
H	6.2842	-0.193	-0.3592

Compound 58			
N_i = 0	E = -953.699565001		
C	4.1406	-1.3696	-0.2878
C	3.8482	-0.1493	0.2936
C	2.5304	0.326	0.3379
C	1.4992	-0.443	-0.2114
C	1.8081	-1.6795	-0.7989
C	3.1137	-2.1332	-0.8378
C	0.0914	-0.0214	-0.1632
C	-0.3946	1.2238	-0.3823
C	-1.8071	1.5273	-0.3103
O	-2.1985	2.6859	-0.5007
C	-2.6675	0.391	-0.026
C	-4.065	0.5107	0.0611
C	-4.8403	-0.5964	0.3203
C	-4.2346	-1.8507	0.4986
C	-2.8657	-1.9942	0.4194
C	-2.0887	-0.8652	0.1565
O	-0.7459	-1.0529	0.0962
O	0.4423	2.2544	-0.6904
O	2.3581	1.5116	0.9939
O	4.8514	0.597	0.8404
H	5.1688	-1.7116	-0.3095
H	1.0109	-2.2726	-1.2292
H	3.3418	-3.0863	-1.2995
H	-4.514	1.4865	-0.0811
H	-5.9174	-0.5038	0.387
H	-4.8487	-2.72	0.703
H	-2.3817	-2.9534	0.5563
H	-0.1021	3.0559	-0.7473
H	1.7061	2.0587	0.5185
H	4.4691	1.4211	1.169

Table S4. (cont.)

Compound 59			
N_i = 0	E = -953.703022402		
C	-4.2051	-0.779	-0.0613
C	-3.7945	0.4277	0.4833
C	-2.4498	0.7837	0.4686
C	-1.4913	-0.0822	-0.0947
C	-1.9443	-1.2935	-0.6405
C	-3.2769	-1.6489	-0.637
C	-0.0452	0.1698	-0.0856
C	0.5964	1.3487	-0.2838
C	2.0347	1.4671	-0.2476
O	2.5666	2.5743	-0.4125
C	2.7534	0.2215	-0.0381
C	4.1567	0.1572	-0.0081
C	4.7905	-1.0505	0.1774
C	4.034	-2.2227	0.3364
C	2.6558	-2.1855	0.3109
C	2.0234	-0.9559	0.1234
O	0.667	-0.9653	0.1175
O	-0.1047	2.4903	-0.5447
O	-2.1387	1.9611	1.0779
O	-5.5337	-1.0699	-0.0141
H	-4.5165	1.1029	0.9264
H	-1.222	-1.9697	-1.0813
H	-3.6041	-2.5867	-1.0714
H	4.7232	1.072	-0.1356
H	5.8723	-1.1002	0.2005
H	4.5371	-3.1716	0.4815
H	2.0566	-3.0794	0.4325
H	0.5399	3.2154	-0.5776
H	-1.371	2.3721	0.6408
H	-5.6954	-1.9243	-0.4283

Compound 60			
N_i = 0	E = -953.698926826		
C	4.3377	-0.4533	0.1774
C	3.8305	0.7429	0.6535
C	2.4686	1.0262	0.588
C	1.5973	0.0776	0.0295
C	2.1207	-1.1288	-0.4555
C	3.4764	-1.3919	-0.3864
C	0.1377	0.2571	-0.031
C	-0.5361	1.3859	-0.358
C	-1.9816	1.4447	-0.3816
O	-2.5497	2.5092	-0.6581
C	-2.6552	0.1919	-0.0852
C	-4.0554	0.0705	-0.0937
C	-4.6469	-1.1414	0.1808
C	-3.8505	-2.2616	0.469
C	-2.4752	-2.1683	0.4845
C	-1.8855	-0.9345	0.2065
O	-0.5304	-0.8879	0.2441
O	0.1322	2.5263	-0.6885
O	2.0525	2.2046	1.151
O	3.9231	-2.589	-0.886
H	5.4012	-0.6597	0.2376
H	4.4914	1.4795	1.0949
H	1.4585	-1.8662	-0.8927
H	-4.6531	0.9451	-0.3212
H	-5.726	-1.2351	0.174
H	-4.3207	-3.2144	0.6825
H	-1.8455	-3.0212	0.706
H	-0.5344	3.2221	-0.8049
H	1.3332	2.587	0.6198
H	4.8784	-2.6444	-0.7839

Table S4. (cont.)

Compound 61			
N_i = 0	E = -953.698263633		
C	-4.463	-0.8139	0.1396
C	-3.573	-1.6788	-0.4785
C	-2.2252	-1.3504	-0.5409
C	-1.747	-0.1384	0.0044
C	-2.6816	0.7237	0.6152
C	-4.0268	0.3827	0.6868
C	-0.3123	0.1769	-0.0396
C	0.2545	1.3598	-0.3708
C	1.6894	1.5623	-0.3636
O	2.1518	2.6742	-0.6429
C	2.4796	0.387	-0.0363
C	3.8842	0.4063	-0.0193
C	4.5883	-0.7385	0.2785
C	3.9033	-1.9297	0.5657
C	2.5248	-1.9744	0.5566
C	1.8232	-0.8081	0.2553
O	0.4662	-0.8937	0.2708
O	-0.5162	2.4277	-0.7129
O	-1.4197	-2.2438	-1.1827
O	-2.3126	1.8945	1.2144
H	-5.5135	-1.0758	0.192
H	-3.9039	-2.613	-0.9152
H	-4.7134	1.0673	1.169
H	4.3958	1.3339	-0.247
H	5.6713	-0.7237	0.2909
H	4.4621	-2.8285	0.799
H	1.9786	-2.8831	0.7774
H	0.0847	3.1785	-0.8454
H	-0.4904	-2.0732	-0.9917
H	-1.6383	2.3466	0.6795

Compound 62			
N_i = 0	E = -953.699845708		
C	4.0398	-0.8026	-0.1369
C	3.6192	0.4324	0.3689
C	2.2734	0.7459	0.4029
C	1.3249	-0.1692	-0.0671
C	1.7503	-1.405	-0.5611
C	3.1022	-1.7155	-0.5974
C	-0.1084	0.1473	-0.0415
C	-0.679	1.3717	-0.1893
C	-2.1144	1.5487	-0.1758
O	-2.5933	2.6838	-0.3233
C	-2.8977	0.3374	-0.0049
C	-4.303	0.3452	0.0206
C	-4.9993	-0.8315	0.1776
C	-4.305	-2.0451	0.3126
C	-2.9269	-2.0791	0.2901
C	-2.2307	-0.8797	0.1303
O	-0.8785	-0.9568	0.1193
O	0.0627	2.4926	-0.3899
O	4.5248	1.3421	0.8391
O	5.3839	-1.026	-0.1366
H	1.9768	1.7024	0.812
H	1.0303	-2.1238	-0.9306
H	3.44	-2.6692	-0.9887
H	-4.8208	1.291	-0.0865
H	-6.0823	-0.8254	0.1973
H	-4.8572	-2.9695	0.4357
H	-2.3754	-3.0055	0.3921
H	-0.5756	3.2148	-0.5009
H	5.4148	0.9829	0.747
H	5.5806	-1.8877	-0.5185

Table S4. (cont.)

Compound 63			
N_i = 0	E = -953.701525563		
C	-4.174	-0.5267	0.0504
C	-3.6828	0.6961	0.4934
C	-2.3207	0.9739	0.475
C	-1.4399	0.0011	0.0035
C	-1.9122	-1.2362	-0.4386
C	-3.2787	-1.4844	-0.4142
C	0.0107	0.2546	-0.0211
C	0.6258	1.4375	-0.2737
C	2.0711	1.5506	-0.2947
O	2.5941	2.6469	-0.5406
C	2.8028	0.3245	-0.032
C	4.2078	0.2738	-0.0169
C	4.8549	-0.9152	0.2298
C	4.1112	-2.0836	0.4652
C	2.7332	-2.06	0.4561
C	2.0865	-0.8482	0.2064
O	0.7327	-0.868	0.2135
O	-0.0696	2.5689	-0.5505
O	-4.5146	1.6697	0.9688
O	-3.7057	-2.7012	-0.8643
H	-5.2404	-0.731	0.0663
H	-1.9675	1.9276	0.8414
H	-1.2366	-1.9964	-0.8086
H	4.7644	1.1847	-0.2023
H	5.9373	-0.9541	0.2424
H	4.6255	-3.018	0.6581
H	2.1438	-2.9504	0.6373
H	0.5948	3.2547	-0.7218
H	-5.4265	1.3617	0.9431
H	-4.665	-2.7541	-0.8028

Compound 64			
N_i = 0	E = -953.708225588		
C	-5.5491	-0.69	0.0028
C	-4.6049	-1.5965	-0.4686
C	-3.2564	-1.2659	-0.4648
C	-2.8436	-0.0154	0.004
C	-3.7947	0.8904	0.4815
C	-5.1408	0.5518	0.4798
C	-1.4157	0.3467	-0.0122
C	-0.9301	1.6072	-0.1093
C	0.4833	1.8574	-0.1172
O	0.9673	3.0099	-0.1967
C	1.3256	0.6826	-0.0376
C	2.7339	0.7725	-0.0443
C	3.5036	-0.3736	0.0302
C	2.8793	-1.6257	0.1164
C	1.4973	-1.7438	0.126
C	0.7446	-0.5859	0.0474
O	-0.6117	-0.7301	0.0658
O	3.3682	1.9621	-0.1206
O	4.8702	-0.3546	0.0275
H	-6.6011	-0.9517	0.0019
H	-4.9187	-2.5643	-0.8426
H	-2.5259	-1.9725	-0.8393
H	-3.4844	1.8522	0.8725
H	-5.8713	1.2568	0.8593
O	3.6275	-2.7508	0.1921
H	1.0255	-2.715	0.1946
H	2.6668	2.6526	-0.166
H	5.1857	0.5551	-0.0154
H	-1.6082	2.4447	-0.1987
H	4.5647	-2.5203	0.1842

Table S4. (cont.)

Compound 65			
N_i = 0	E = -953.702726759		
C	5.4116	0.3412	0.0068
C	4.9339	-0.897	-0.4119
C	3.5702	-1.1548	-0.413
C	2.6712	-0.1708	0.0076
C	3.1539	1.0752	0.419
C	4.5195	1.3252	0.4211
C	1.2243	-0.4419	0.0211
C	0.6517	-1.668	0.1128
C	-0.7734	-1.8304	0.1075
O	-1.3297	-2.9459	0.1743
C	-1.5512	-0.5991	0.0288
C	-2.9535	-0.6011	0.0264
C	-3.6342	0.6092	-0.042
C	-2.9348	1.8103	-0.1089
C	-1.551	1.8293	-0.1096
C	-0.8698	0.6173	-0.0406
O	0.4911	0.6806	-0.0545
O	-3.6814	-1.7469	0.0837
O	-4.9991	0.6495	-0.0476
H	6.4773	0.5399	0.0068
H	5.6243	-1.6625	-0.7464
H	3.207	-2.1154	-0.7588
H	2.4639	1.8428	0.7477
H	4.8875	2.2905	0.749
O	-0.8971	3.0252	-0.176
H	-3.0399	-2.488	0.1263
H	-5.3453	-0.2487	0.0095
H	1.272	-2.5491	0.2013
H	-3.4833	2.7436	-0.1612
H	0.0557	2.8783	-0.1644

Compound 66			
N_i = 0	E = -953.706886532		
C	-5.2642	-0.0328	-0.1515
C	-4.4906	-1.0897	0.2941
C	-3.1002	-0.986	0.3311
C	-2.4774	0.2099	-0.0746
C	-3.2851	1.2629	-0.5294
C	-4.6621	1.1524	-0.5713
C	-1.0216	0.4069	-0.0216
C	-0.401	1.5932	0.1903
C	1.0296	1.6905	0.2095
O	1.6336	2.7685	0.3925
C	1.7506	0.4392	0.019
C	3.1553	0.3828	0.0342
C	3.8006	-0.8371	-0.1456
C	3.0541	-1.9955	-0.3312
C	1.6681	-1.9649	-0.3458
C	1.0367	-0.7459	-0.1724
O	-0.3315	-0.7361	-0.205
O	3.9157	1.4844	0.2206
O	5.1649	-0.9189	-0.142
H	-6.343	-0.1364	-0.1786
H	-4.9458	-2.0176	0.6202
O	-2.4359	-2.0756	0.8053
H	-2.8119	2.1757	-0.8713
H	-5.2618	1.9777	-0.9347
H	3.2975	2.2411	0.3325
H	5.5392	-0.0383	-0.0218
H	-0.9845	2.4857	0.3677
H	3.5789	-2.934	-0.4673
H	1.0847	-2.8647	-0.4913
H	-1.4857	-1.9829	0.6639

c

Table S4. (cont.)

Compound 67			
N_i = 0	E = -953.709029716		
C	-5.0874	0.1497	0.1579
C	-4.3011	-0.9255	-0.2474
C	-2.9161	-0.8162	-0.2736
C	-2.3116	0.3852	0.0956
C	-3.0935	1.4687	0.5048
C	-4.4749	1.3392	0.5334
C	-0.8429	0.5023	0.0432
C	-0.1594	1.6565	-0.155
C	1.2735	1.6705	-0.1958
O	1.9388	2.7139	-0.3722
C	1.9191	0.3759	-0.0324
C	3.3179	0.2358	-0.0677
C	3.8913	-1.0215	0.0903
C	3.0795	-2.1349	0.28
C	1.6988	-2.0216	0.3147
C	1.1354	-0.7659	0.1568
O	-0.2275	-0.6804	0.1999
O	4.1409	1.2921	-0.255
O	5.2485	-1.1842	0.0605
H	-6.1677	0.0514	0.1786
O	-4.8407	-2.1199	-0.636
H	-2.631	2.3967	0.817
H	-5.0862	2.1733	0.8577
H	3.5673	2.0856	-0.3506
H	5.6701	-0.3258	-0.0638
H	-0.6934	2.5844	-0.3053
H	3.5493	-3.1041	0.4016
H	1.0658	-2.8869	0.4619
H	-2.324	-1.6631	-0.5974
H	-5.8018	-2.0717	-0.6062

Compound 68			
N_i = 0	E = -953.710917861		
C	-4.9924	-0.2571	0.0152
C	-4.1497	-1.2903	-0.3918
C	-2.7785	-1.0952	-0.396
C	-2.2294	0.1305	-0.0072
C	-3.0894	1.1573	0.3988
C	-4.4593	0.9687	0.4122
C	-0.776	0.334	-0.0267
C	-0.1509	1.5356	-0.1365
C	1.2759	1.6332	-0.1298
O	1.8867	2.7224	-0.2162
C	1.9929	0.3693	-0.0269
C	3.3977	0.3128	-0.0178
C	4.0403	-0.917	0.0776
C	3.2908	-2.0853	0.1645
C	1.905	-2.0539	0.1575
C	1.2727	-0.8247	0.0613
O	-0.0935	-0.8195	0.0679
O	4.16	1.4272	-0.0944
O	5.4054	-0.9979	0.0906
O	-6.3463	-0.3924	0.0492
H	-2.6889	2.1085	0.7291
H	-5.124	1.76	0.7377
H	3.5402	2.1896	-0.156
H	5.778	-0.1113	0.0196
H	-0.7324	2.4406	-0.244
H	3.8135	-3.0318	0.2393
H	1.3207	-2.9622	0.2253
H	-2.1323	-1.9023	-0.7181
H	-4.5707	-2.2401	-0.7033
H	-6.6003	-1.2777	-0.2325

Table S4. (cont.)

Compound 69			
N_i = 0	E = -953.706244557		
C	5.2882	-0.6334	0.0227
C	4.3121	-1.5378	0.4279
C	2.9726	-1.1723	0.4147
C	2.6004	0.1102	0.0004
C	3.5839	1.0134	-0.4126
C	4.9207	0.6408	-0.3994
C	1.1819	0.505	0.007
C	0.7113	1.7703	0.0875
C	-0.7018	2.0444	0.0867
O	-1.1586	3.207	0.146
C	-1.5695	0.8863	0.0265
C	-2.9777	0.979	0.038
C	-3.7512	-0.165	-0.0111
C	-3.139	-1.4161	-0.0714
C	-1.7512	-1.5446	-0.088
C	-0.9924	-0.3875	-0.0393
O	0.3553	-0.5592	-0.0628
O	-3.5871	2.1862	0.0995
H	3.3083	2.0014	-0.7617
H	5.6762	1.3451	-0.7275
H	-2.8764	2.8637	0.1297
H	1.3987	2.601	0.1687
O	-3.9293	-2.5155	-0.1108
O	-1.2184	-2.8019	-0.1525
H	2.2175	-1.8787	0.7374
H	4.5933	-2.5312	0.7577
H	-4.832	-0.0979	0
H	6.3332	-0.9218	0.0316
H	-3.3855	-3.3126	-0.1337
H	-0.2574	-2.7582	-0.0947

Compound 70			
N_i = 0	E = -953.713980135		
C	5.1697	-0.2662	-0.1615
C	4.6511	0.9426	-0.6229
C	3.2855	1.1531	-0.5804
C	2.4079	0.1787	-0.0825
C	2.946	-1.0437	0.3631
C	4.325	-1.2483	0.3249
C	0.9713	0.4853	-0.0245
C	0.4388	1.7142	0.1571
C	-0.9862	1.9086	0.1806
O	-1.5058	3.0368	0.3325
C	-1.7888	0.7134	0.0343
C	-3.2041	0.737	0.0586
C	-3.9299	-0.4323	-0.073
C	-3.253	-1.6451	-0.2277
C	-1.8635	-1.7128	-0.2573
C	-1.1659	-0.5285	-0.1263
O	0.195	-0.6145	-0.1704
O	-3.8598	1.9033	0.211
O	2.2076	-2.0656	0.8779
H	4.7146	-2.1943	0.6825
H	-3.1766	2.6087	0.2893
H	1.0834	2.5696	0.3037
O	-3.9242	-2.8138	-0.3581
H	2.8762	2.084	-0.9547
H	5.3063	1.7083	-1.0197
H	-5.0134	-0.4042	-0.0537
H	6.2382	-0.4477	-0.1881
H	-4.8747	-2.6609	-0.3238
H	-1.3465	-2.6551	-0.3803
H	1.2668	-1.9141	0.7264

Table S4. (cont.)

Compound 71			
N_i = 0	E = -953.716078346		
C	4.9973	-0.0026	0.1841
C	4.4482	-1.2144	0.5852
C	3.0767	-1.4224	0.546
C	2.24	-0.3964	0.0986
C	2.7803	0.8265	-0.2983
C	4.1567	1.0144	-0.2603
C	0.7815	-0.5999	0.0366
C	0.165	-1.7913	-0.1316
C	-1.2693	-1.8815	-0.1794
O	-1.8698	-2.9703	-0.3275
C	-1.98	-0.6272	-0.0569
C	-3.393	-0.5447	-0.0967
C	-4.0316	0.6751	0.0212
C	-3.2675	1.8354	0.1775
C	-1.8779	1.7991	0.2213
C	-1.265	0.5659	0.1013
O	0.096	0.5558	0.1521
O	-4.1321	-1.6606	-0.25
O	4.6343	2.2262	-0.6752
H	-3.5012	-2.4141	-0.3179
H	0.7492	-2.6933	-0.2495
O	-3.8501	3.0533	0.2933
H	2.663	-2.3658	0.8799
H	5.1014	-2.0032	0.9398
H	-5.1138	0.7265	-0.0107
H	6.0698	0.1585	0.2141
H	-4.8092	2.9763	0.2434
H	-1.2947	2.7021	0.3425
H	2.1456	1.6295	-0.6517
H	5.5956	2.2334	-0.6241

Compound 72			
N_i = 0	E = -953.717513680		
C	-4.8899	-0.4066	0
C	-3.9594	-1.4431	0
C	-2.6057	-1.1506	0
C	-2.1547	0.1739	0
C	-3.1062	1.2025	0
C	-4.4591	0.9197	0
C	-0.715	0.469	0
C	-0.1452	1.6998	0
C	1.2803	1.8592	0
O	1.832	2.9853	0
C	2.0527	0.6358	0
C	3.4678	0.6244	0
C	4.1623	-0.5709	0
C	3.4516	-1.7745	0
C	2.0615	-1.8075	0
C	1.3927	-0.5974	0
O	0.0336	-0.6542	0
O	4.1548	1.7838	0
H	3.4889	2.5105	0
H	-0.7561	2.591	0
O	4.0923	-2.9689	0
H	-1.8953	-1.967	0
H	-4.2987	-2.4733	0
H	5.2463	-0.5703	0
O	-6.2316	-0.6325	0
H	5.0466	-2.8372	0
H	1.5195	-2.7438	0
H	-2.8002	2.2411	0
H	-5.1905	1.7192	0
H	-6.4107	-1.5791	0

Table S4. (cont.)

Compound 73			
N_i = 0	E = -953.704692897		
C	-5.0151	-0.138	0.1172
C	-4.409	-1.3224	0.5344
C	-3.0316	-1.4248	0.5008
C	-2.2254	-0.3655	0.056
C	-2.8527	0.8305	-0.3451
C	-4.2443	0.9253	-0.3163
C	-0.7695	-0.5582	0.0111
C	-0.135	-1.7432	-0.1532
C	1.2983	-1.828	-0.1579
O	1.9055	-2.9103	-0.2911
C	2.0146	-0.5662	-0.0158
C	3.4232	-0.485	-0.0282
C	4.0371	0.749	0.095
C	3.2709	1.9061	0.2234
C	1.8873	1.8581	0.2335
C	1.2775	0.6113	0.1182
O	-0.0837	0.5962	0.1508
O	4.1832	-1.6008	-0.1586
H	3.5656	-2.3589	-0.2401
H	-0.7051	-2.6499	-0.2984
H	-2.5572	-2.3373	0.8414
H	-5.0063	-2.1531	0.8895
H	5.1184	0.8094	0.0877
O	1.0769	2.9503	0.3449
O	-2.1953	1.9295	-0.8047
H	-4.702	1.8534	-0.6382
H	-6.0946	-0.0404	0.138
H	3.7619	2.8691	0.3154
H	1.6137	3.7483	0.39
H	-1.2451	1.8473	-0.654

Compound 74			
N_i = 0	E = -953.706978362		
C	4.816	-0.3762	-0.1374
C	4.1825	-1.5527	-0.5192
C	2.7989	-1.6565	-0.4952
C	2.0367	-0.5606	-0.0822
C	2.6622	0.6285	0.2924
C	4.0489	0.7127	0.2683
C	0.5671	-0.6495	-0.0333
C	-0.1515	-1.7864	0.1206
C	-1.588	-1.7617	0.1567
O	-2.2749	-2.795	0.2897
C	-2.2048	-0.4467	0.0428
C	-3.6009	-0.2517	0.0789
C	-4.1145	1.0294	-0.0286
C	-3.2605	2.1217	-0.168
C	-1.887	1.9611	-0.2031
C	-1.3768	0.6695	-0.0962
O	-0.023	0.555	-0.1435
O	-4.4476	-1.3023	0.2188
H	-3.8946	-2.1097	0.2848
H	0.3522	-2.736	0.2361
H	2.3203	-2.5741	-0.8135
H	4.7789	-2.3968	-0.8452
H	-5.1873	1.1762	-0.002
O	-1.0729	3.0507	-0.3393
O	4.6094	1.8966	0.6587
H	5.898	-0.2977	-0.1552
H	-3.6694	3.1221	-0.2514
H	-0.1504	2.7706	-0.345
H	2.0851	1.4858	0.6164
H	5.5694	1.8342	0.6211

Table S4. (cont.)

Compound 75			
N_i = 0	E = -953.708908455		
C	4.7343	0.1256	-0.0214
C	4.2156	-1.1209	0.3275
C	2.8453	-1.3146	0.3392
C	1.9727	-0.2733	0.0077
C	2.5093	0.9764	-0.3253
C	3.8779	1.1768	-0.3441
C	0.5212	-0.4827	-0.0024
C	-0.1152	-1.6785	-0.0836
C	-1.5462	-1.7671	-0.0797
O	-2.155	-2.8571	-0.1372
C	-2.2637	-0.4995	-0.0176
C	-3.671	-0.4152	-0.0268
C	-4.2816	0.8262	0.0272
C	-3.5129	1.9867	0.09
C	-2.1305	1.9344	0.1
C	-1.523	0.682	0.045
O	-0.1639	0.6751	0.0638
O	-4.4343	-1.5357	-0.0837
H	-3.8181	-2.2988	-0.1124
H	0.4553	-2.5932	-0.1633
H	2.4599	-2.2857	0.6259
H	4.8867	-1.931	0.5914
H	-5.363	0.8881	0.0209
O	-1.4023	3.0902	0.16
O	6.0723	0.3696	-0.0584
H	-3.9971	2.9555	0.1324
H	-0.4608	2.8834	0.1714
H	1.8525	1.7976	-0.5846
H	4.2929	2.1411	-0.6122
H	6.563	-0.4307	0.1587

Compound 76			
N_i = 0	E = -953.708578284		
C	-4.636	-0.5949	0.2502
C	-3.9804	0.5501	-0.1628
C	-2.5806	0.5916	-0.2162
C	-1.8392	-0.5432	0.1392
C	-2.5196	-1.6945	0.5635
C	-3.9005	-1.719	0.6177
C	-0.3713	-0.5648	0.061
C	0.3822	-1.6528	-0.2172
C	1.816	-1.5706	-0.2543
O	2.5388	-2.5605	-0.4918
C	2.3827	-0.2515	-0.0169
C	3.7749	-0.0072	-0.0512
C	4.2614	1.2724	0.1691
C	3.3729	2.3139	0.4238
C	2.0016	2.1117	0.4654
C	1.5332	0.8268	0.244
O	0.1803	0.6456	0.2964
O	-2.0656	1.7689	-0.6698
O	-4.7068	1.6484	-0.5239
H	-5.7192	-0.5953	0.2884
H	-1.9509	-2.5645	0.8679
H	-4.414	-2.6113	0.9543
O	4.6412	-1.011	-0.2992
H	5.3301	1.4455	0.1401
H	3.7647	3.3101	0.5932
H	1.3052	2.9163	0.6625
H	-1.1212	1.8255	-0.4779
H	-4.1	2.363	-0.7531
H	-0.0914	-2.6011	-0.4297
H	4.1096	-1.8277	-0.4306

Table S4. (cont.)

Compound 77					Compound 78			
N_i = 0	E = -953.713639254				N_i = 0	E = -953.708629162		
C	4.5806	-0.0939	-0.0707		C	4.6338	0.433	-0.0595
C	3.8345	1.0131	0.3025		C	3.7857	1.4762	-0.383
C	2.4457	0.9531	0.3043		C	2.4024	1.3136	-0.365
C	1.7825	-0.2388	-0.06		C	1.8619	0.0611	-0.0243
C	2.5722	-1.3332	-0.4442		C	2.7326	-0.9839	0.3156
C	3.9494	-1.2797	-0.4566		C	4.1024	-0.8045	0.2985
C	0.3266	-0.3897	-0.0241		C	0.4172	-0.2144	-0.0392
C	-0.3432	-1.5525	0.1733		C	-0.1479	-1.4184	-0.2883
C	-1.7756	-1.5942	0.1745		C	-1.5737	-1.5868	-0.2679
O	-2.4187	-2.654	0.3395		O	-2.1228	-2.6885	-0.4787
C	-2.4505	-0.3167	-0.0046		C	-2.3516	-0.3857	-0.0047
C	-3.8585	-0.1932	0.0075		C	-3.7654	-0.3881	0.0178
C	-4.4487	1.0511	-0.1548		C	-4.458	0.7897	0.254
C	-3.6476	2.1773	-0.3261		C	-3.7545	1.9724	0.4678
C	-2.2634	2.0943	-0.3408		C	-2.3683	2.0118	0.4557
C	-1.6918	0.8425	-0.1796		C	-1.6927	0.8255	0.2196
O	-0.3278	0.7787	-0.2086		O	-0.3281	0.881	0.2212
O	1.8128	2.0858	0.7083		O	1.6656	2.4065	-0.7282
O	5.9338	0.0298	-0.0513		O	4.8928	-1.8705	0.6478
H	4.3269	1.9325	0.5952		H	5.7089	0.5785	-0.0731
H	2.0853	-2.2485	-0.7584		H	4.1889	2.4438	-0.6575
H	4.5372	-2.1363	-0.7658		H	2.3343	-1.9461	0.6135
O	-4.6388	-1.2805	0.1785		O	-4.4513	-1.5307	-0.1914
H	-5.5288	1.1309	-0.1448		H	-5.5409	0.7745	0.2677
H	-4.1197	3.1448	-0.4521		H	-4.3064	2.8872	0.6501
H	-1.6341	2.9647	-0.474		H	-1.8155	2.9271	0.6231
H	0.8624	2.0135	0.5535		H	0.7361	2.2714	-0.5084
H	6.3453	-0.7963	-0.3273		H	5.8196	-1.6153	0.6029
H	0.1996	-2.4691	0.3565		H	0.4713	-2.272	-0.5257
H	-4.0368	-2.0519	0.2816		H	-3.789	-2.242	-0.3426

Table S4. (cont.)

Compound 79			
N_i = 0	E = -953.708057197		
C	-4.7913	-0.342	-0.0153
C	-4.2606	0.827	0.5102
C	-2.8834	1.0131	0.5091
C	-2.0187	0.034	-0.0159
C	-2.5902	-1.1521	-0.5201
C	-3.9689	-1.3321	-0.5282
C	-0.5652	0.2539	-0.0518
C	0.0603	1.3898	-0.4286
C	1.4947	1.4783	-0.4417
O	2.0988	2.518	-0.7786
C	2.2085	0.2722	-0.0528
C	3.6208	0.2008	-0.0346
C	4.252	-0.9776	0.3325
C	3.4885	-2.0893	0.6799
C	2.1024	-2.0566	0.672
C	1.4872	-0.8702	0.3044
O	0.1216	-0.8553	0.3128
O	-2.3307	2.1369	1.0339
O	-1.8513	-2.1604	-1.0618
H	-5.8656	-0.486	-0.0169
H	-4.9006	1.596	0.9282
H	-4.3748	-2.2504	-0.9339
H	5.3343	-1.0187	0.3427
H	3.9932	-3.0052	0.9649
H	1.504	-2.917	0.9417
H	-3.028	2.7039	1.3801
H	-0.9181	-2.0664	-0.8371
O	4.3644	1.2739	-0.3746
H	3.7386	1.997	-0.6053
H	-0.5213	2.2478	-0.7334

Compound 80			
N_i = 0	E = -953.712046115		
C	4.3989	-0.266	-0.0713
C	3.6431	0.8867	0.1748
C	2.2634	0.819	0.1838
C	1.6145	-0.3995	-0.0488
C	2.3741	-1.5433	-0.302
C	3.759	-1.4735	-0.3122
C	0.1466	-0.4628	-0.0198
C	-0.5953	-1.5909	0.1141
C	-2.0265	-1.54	0.1257
O	-2.7382	-2.5631	0.2384
C	-2.6143	-0.2141	0.0121
C	-4.0118	-0.0005	0.0286
C	-4.5197	1.285	-0.0746
C	-3.6455	2.3635	-0.1924
C	-2.2702	2.1914	-0.21
C	-1.7773	0.8989	-0.1061
O	-0.4225	0.7523	-0.1311
O	4.2553	2.0843	0.4128
O	5.7504	-0.109	-0.0584
H	1.7012	1.7219	0.3858
H	1.8983	-2.4932	-0.5095
H	4.3541	-2.3572	-0.5152
H	-5.5923	1.4348	-0.0611
H	-4.0526	3.3648	-0.2722
H	-1.5871	3.0258	-0.3016
H	5.2116	1.9661	0.3715
H	6.1916	-0.952	-0.209
H	-0.114	-2.5517	0.2291
O	-4.8616	-1.0427	0.1458
H	-4.3112	-1.8562	0.211

Table S4. (cont.)

Compound 81			
N_i = 0	E = -953.713391332		
C	-4.4711	0.2109	-0.0137
C	-3.6315	1.2743	-0.3266
C	-2.2497	1.1243	-0.3173
C	-1.7081	-0.1198	0.0026
C	-2.5312	-1.198	0.3249
C	-3.9093	-1.0192	0.3141
C	-0.2435	-0.2958	-0.0172
C	0.3999	-1.4614	-0.259
C	1.8342	-1.5256	-0.2682
O	2.4592	-2.5869	-0.4802
C	2.5229	-0.2672	-0.0307
C	3.9335	-0.1638	-0.0312
C	4.5411	1.0607	0.1973
C	3.7546	2.1879	0.4247
C	2.3702	2.125	0.4306
C	1.7766	0.8926	0.2009
O	0.4142	0.8541	0.2154
O	-4.1247	2.5031	-0.6583
O	-4.6825	-2.0951	0.6422
H	-5.5493	0.3383	-0.0206
H	-1.6193	1.9652	-0.5744
H	-2.1238	-2.1618	0.6024
O	4.6989	-1.2522	-0.2559
H	5.6222	1.1254	0.1947
H	4.2401	3.1407	0.6016
H	1.7549	2.9975	0.607
H	-5.0871	2.4864	-0.6465
H	-5.6146	-1.8555	0.6116
H	-0.1627	-2.3599	-0.4711
H	4.0875	-2.0098	-0.3959

Compound 82			
N_i = 0	E = -953.700442243		
C	-5.3376	-0.8724	-0.0103
C	-4.2929	-1.7231	-0.3559
C	-2.984	-1.2597	-0.3506
C	-2.7091	0.0663	-0.0016
C	-3.762	0.9151	0.3528
C	-5.068	0.4457	0.3456
C	-1.3195	0.557	-0.0129
C	-0.9309	1.8515	-0.0952
C	0.4596	2.2347	-0.0946
O	0.8247	3.4184	-0.1505
C	1.4115	1.1228	-0.037
C	2.7992	1.3141	-0.0527
C	3.6408	0.2271	-0.0038
C	3.1179	-1.0779	0.0593
C	1.7495	-1.2826	0.0731
C	0.9061	-0.1709	0.0278
O	-0.4248	-0.446	0.0565
H	-3.5672	1.9382	0.6516
H	-5.8771	1.1101	0.6261
H	-1.6748	2.6321	-0.1766
O	4.009	-2.1001	0.1037
O	1.2926	-2.5613	0.1326
H	-2.176	-1.9261	-0.6268
H	-4.4962	-2.7511	-0.633
O	4.9913	0.4135	-0.0189
H	-6.3589	-1.2359	-0.0137
H	3.5513	-2.949	0.1291
H	0.3289	-2.5725	0.0868
H	3.2098	2.3144	-0.1049
H	5.4354	-0.4422	0.0097

Table S4. (cont.)

Compound 83			
N_i = 0	E = -953.705746910		
C	5.2389	-0.4972	-0.181
C	4.3286	-1.4245	0.2946
C	2.9682	-1.1222	0.3441
C	2.5156	0.1413	-0.0811
C	3.4581	1.0583	-0.5684
C	4.8055	0.7524	-0.6207
C	1.1025	0.5447	-0.011
C	0.6551	1.8051	0.1916
C	-0.7533	2.114	0.2294
O	-1.1778	3.2676	0.395
C	-1.6433	0.9638	0.0753
C	-3.0423	1.0874	0.1127
C	-3.8395	-0.0198	-0.0304
C	-3.2571	-1.2954	-0.2131
C	-1.8864	-1.4377	-0.2516
C	-1.0973	-0.3018	-0.1074
O	0.2539	-0.4951	-0.1679
H	3.1146	2.0221	-0.9254
H	5.5119	1.4763	-1.0082
H	1.3632	2.6079	0.3451
O	-4.041	-2.3891	-0.3545
O	2.1596	-2.0953	0.8491
H	4.6515	-2.4011	0.6361
O	-5.2028	-0.0024	-0.0112
H	6.2918	-0.7531	-0.2158
H	-4.9694	-2.1276	-0.3111
H	-3.4897	2.0647	0.2548
H	-5.5269	0.8938	0.1261
H	-1.434	-2.411	-0.3937
H	1.2318	-1.8685	0.7097

Compound 84			
N_i = 0	E = -953.707895384		
C	5.0772	-0.2291	0.1709
C	4.156	-1.2009	-0.2094
C	2.7984	-0.9084	-0.2554
C	2.3556	0.3717	0.0775
C	3.2735	1.3511	0.4666
C	4.6257	1.0411	0.509
C	0.9147	0.6808	0.0135
C	0.383	1.9137	-0.1562
C	-1.0424	2.1235	-0.2039
O	-1.548	3.2485	-0.3439
C	-1.8473	0.9097	-0.084
C	-3.2524	0.9341	-0.1238
C	-3.9678	-0.2306	-0.0072
C	-3.2944	-1.4646	0.1498
C	-1.9181	-1.5088	0.1917
C	-1.2091	-0.317	0.0732
O	0.1492	-0.4195	0.1293
H	2.9372	2.3399	0.7525
H	5.3404	1.7954	0.8167
H	1.035	2.7688	-0.2723
O	-3.9982	-2.6154	0.262
O	-5.3288	-0.3158	-0.0291
H	6.1343	-0.471	0.2052
H	-4.9425	-2.4204	0.2154
H	-3.7684	1.8798	-0.2462
H	-5.7228	0.5561	-0.1354
H	-1.3988	-2.4508	0.3137
H	2.1016	-1.6789	-0.5604
O	4.5355	-2.4687	-0.5524
H	5.4927	-2.5491	-0.4898

Table S4. (cont.)

Compound 85			
N_i = 0	E = -953.709097155		
C	4.9558	-0.5686	0
C	3.967	-1.5493	0
C	2.6321	-1.1786	0
C	2.2582	0.1695	0
C	3.2681	1.1405	0
C	4.6027	0.7801	0
C	0.8367	0.5486	0
C	0.3432	1.8129	0
C	-1.07	2.0788	0
O	-1.5341	3.2318	0
C	-1.9229	0.8923	0
C	-3.3254	0.9741	0
C	-4.0855	-0.1676	0
C	-3.4601	-1.4358	0
C	-2.0856	-1.5361	0
C	-1.331	-0.3664	0
O	0.0227	-0.5243	0
H	3.0244	2.1954	0
H	5.3789	1.5362	0
H	1.0129	2.6611	0
O	-4.2071	-2.5655	0
O	-5.4494	-0.192	0
H	-5.1435	-2.3314	0
H	-3.8047	1.9466	0
H	-5.8002	0.7046	0
H	-1.6026	-2.505	0
H	1.8757	-1.9525	0
H	4.2454	-2.5977	0
O	6.2821	-0.8743	0
H	6.4015	-1.8303	0

Compound 86			
N_i = 0	E = -953.702703229		
C	-5.0909	-0.2081	-0.1907
C	-4.2469	-1.157	0.3595
C	-2.8729	-0.9302	0.4244
C	-2.3386	0.2749	-0.0666
C	-3.2143	1.2177	-0.6234
C	-4.5764	0.9879	-0.6884
C	-0.905	0.5962	0.0023
C	-0.3926	1.8328	0.2034
C	1.0268	2.0746	0.2382
O	1.5095	3.2008	0.4056
C	1.8718	0.879	0.0753
C	3.2666	0.9499	0.1058
C	4.0037	-0.208	-0.0543
C	3.3723	-1.4428	-0.2451
C	1.9943	-1.5209	-0.2756
C	1.2466	-0.3473	-0.1101
O	-0.1122	-0.4837	-0.1587
H	-2.805	2.1374	-1.0247
H	-5.2317	1.7288	-1.1297
H	-1.0615	2.6682	0.3586
O	5.3643	-0.1064	-0.0209
H	3.7588	1.9028	0.2522
H	5.7685	-0.9712	-0.1465
H	-4.6341	-2.09	0.7518
H	3.957	-2.347	-0.3743
O	1.408	-2.7298	-0.4644
H	0.4491	-2.6407	-0.5001
O	-2.1346	-1.9124	1.0117
H	-1.1926	-1.7253	0.9345
H	-6.1561	-0.4044	-0.2349

Table S4. (cont.)

Compound 87			
N_i = 0	E = -953.705070422		
C	-4.9124	0.0417	0.1444
C	-4.0422	-0.9732	-0.2444
C	-2.6706	-0.7536	-0.2763
C	-2.1638	0.498	0.0737
C	-3.0301	1.521	0.4683
C	-4.3969	1.2822	0.5
C	-0.7093	0.7298	0.0185
C	-0.1067	1.9316	-0.1491
C	1.3263	2.0667	-0.1939
O	1.8928	3.1587	-0.3313
C	2.0775	0.8062	-0.0765
C	3.4744	0.7629	-0.1216
C	4.1127	-0.4569	-0.0069
C	3.3842	-1.643	0.1514
C	2.006	-1.607	0.1954
C	1.3564	-0.3703	0.0789
O	-0.0035	-0.4063	0.1373
H	-2.6437	2.4876	0.7665
H	-5.0727	2.0698	0.8121
H	-0.7103	2.8208	-0.2688
O	5.4771	-0.4737	-0.0562
H	4.0426	1.6761	-0.2452
H	5.7991	-1.3757	0.0415
O	-4.4858	-2.2135	-0.6099
H	3.896	-2.595	0.2404
O	1.311	-2.7621	0.3493
H	0.3634	-2.5809	0.3522
H	-5.9812	-0.144	0.1685
H	-2.0132	-1.5565	-0.5864
H	-5.4469	-2.2428	-0.5645

Compound 88			
N_i = 0	E = -953.706915900		
C	4.8004	-0.3612	-0.0102
C	4.3795	0.9216	0.3387
C	3.0283	1.2234	0.3403
C	2.0783	0.2575	-0.005
C	2.5174	-1.0274	-0.3463
C	3.8657	-1.3364	-0.3534
C	0.6457	0.578	-0.0162
C	0.1042	1.8201	-0.1049
C	-1.3165	2.0364	-0.1035
O	-1.8252	3.1646	-0.1667
C	-2.1373	0.8157	-0.0349
C	-3.5347	0.8529	-0.0462
C	-4.2402	-0.3334	0.0179
C	-3.5777	-1.5657	0.0907
C	-2.1987	-1.6083	0.0996
C	-1.4817	-0.4058	0.0378
O	-0.125	-0.5207	0.0593
H	1.8001	-1.7918	-0.6186
H	4.2047	-2.3292	-0.6247
H	0.7507	2.6828	-0.1918
O	-5.6042	-0.2651	0.0061
H	-4.0533	1.8013	-0.1049
H	-5.9834	-1.1484	0.0575
H	-4.1403	-2.4917	0.1365
O	-1.5669	-2.8079	0.1651
H	-0.611	-2.6784	0.1504
O	6.1139	-0.7171	-0.0281
H	2.7182	2.22	0.6311
H	5.1106	1.6737	0.6149
H	6.6657	0.0263	0.2376

Table S4. (cont.)

Compound 89			
N_i = 0	E = -953.703338902		
C	-4.7497	0.2939	0.247
C	-3.9874	-0.7972	-0.1261
C	-2.59	-0.7063	-0.1873
C	-1.9594	0.5067	0.122
C	-2.7479	1.6008	0.5097
C	-4.1247	1.4959	0.5692
C	-0.4994	0.6681	0.0348
C	0.1436	1.8248	-0.2517
C	1.5807	1.903	-0.2973
O	2.1874	2.9563	-0.5308
C	2.2808	0.6304	-0.0667
C	3.6754	0.5312	-0.122
C	4.2859	-0.69	0.0822
C	3.5153	-1.832	0.3474
C	2.1407	-1.7469	0.4072
C	1.534	-0.5127	0.1987
O	0.1679	-0.4784	0.2731
O	-1.9657	-1.8469	-0.5953
O	-4.6069	-1.9741	-0.4365
H	-5.8278	0.1911	0.2919
H	-2.2662	2.5317	0.7818
H	-4.7199	2.3471	0.8763
H	4.0092	-2.7851	0.5051
H	1.531	-2.6185	0.6114
H	-1.0191	-1.8002	-0.4107
H	-3.9365	-2.6388	-0.6362
H	-0.4276	2.7175	-0.4657
H	4.2688	1.4132	-0.3301
O	5.6488	-0.748	0.0133
H	5.948	-1.6475	0.182

Compound 90			
N_i = 0	E = -953.708248305		
C	-4.6619	-0.1303	-0.0785
C	-3.832	-1.1851	0.2676
C	-2.4517	-1.0183	0.2724
C	-1.882	0.2296	-0.0612
C	-2.7547	1.2672	-0.4221
C	-4.124	1.1083	-0.4373
C	-0.4413	0.4946	-0.0139
C	0.1298	1.7081	0.1966
C	1.555	1.8863	0.2128
O	2.0936	2.99	0.3799
C	2.3403	0.6543	0.0386
C	3.7387	0.6558	0.0704
C	4.4312	-0.5286	-0.0851
C	3.7396	-1.7345	-0.2732
C	2.3608	-1.7485	-0.3063
C	1.672	-0.5504	-0.1494
O	0.3055	-0.6137	-0.1987
O	-1.7337	-2.1107	0.6449
O	-6.0022	-0.3577	-0.0623
H	-4.2516	-2.1467	0.5374
H	-2.3407	2.2246	-0.7142
H	-4.7754	1.9247	-0.7272
H	4.2959	-2.6578	-0.3929
H	1.8111	-2.6705	-0.4509
H	-0.7915	-1.9582	0.4974
H	-6.476	0.4456	-0.3039
H	-0.496	2.5712	0.3768
H	4.2713	1.5874	0.2192
O	5.7962	-0.4836	-0.046
H	6.1593	-1.3663	-0.1719

Table S4. (cont.)

Compound 91			
N_i = 0	E = -953.703240724		
C	4.6831	-0.6622	0.0257
C	3.7599	-1.6337	0.3682
C	2.394	-1.3627	0.3656
C	1.9494	-0.0733	0.0241
C	2.894	0.8978	-0.3345
C	4.246	0.6104	-0.3352
C	0.529	0.3122	0.0591
C	0.0613	1.5513	0.3413
C	-1.3481	1.8459	0.3518
O	-1.795	2.9742	0.5946
C	-2.2258	0.6992	0.0726
C	-3.6202	0.8153	0.0764
C	-4.4033	-0.2941	-0.1721
C	-3.8077	-1.5382	-0.43
C	-2.4349	-1.6651	-0.4382
C	-1.6539	-0.5419	-0.1862
O	-0.297	-0.7172	-0.2121
O	1.5763	-2.3924	0.7437
O	5.1134	1.6076	-0.7057
H	5.7431	-0.8934	0.0268
H	4.0896	-2.6283	0.6446
H	2.5671	1.8864	-0.6334
H	-4.4343	-2.4021	-0.6245
H	-1.959	-2.618	-0.6353
H	0.6592	-2.1882	0.5247
H	6.0159	1.2734	-0.69
H	0.7553	2.3446	0.5828
H	-4.0787	1.7758	0.2783
O	-5.7605	-0.1385	-0.1567
H	-6.1932	-0.9838	-0.3162

Compound 92			
N_i = 0	E = -953.702802856		
C	-4.8326	-0.6428	-0.064
C	-4.3951	0.5708	-0.5748
C	-3.0399	0.878	-0.5378
C	-2.1064	-0.0222	0.0087
C	-2.582	-1.2547	0.4979
C	-3.9389	-1.5577	0.4691
C	-0.6779	0.3247	0.0832
C	-0.1685	1.5002	0.5136
C	1.2514	1.7375	0.5696
O	1.7356	2.8107	0.9511
C	2.088	0.6009	0.1583
C	3.486	0.6643	0.1825
C	4.2304	-0.4341	-0.1969
C	3.5929	-1.6149	-0.6082
C	2.2167	-1.6896	-0.6365
C	1.4731	-0.5781	-0.2514
O	0.1109	-0.7021	-0.3029
O	-2.5714	2.0477	-1.0461
O	-1.7695	-2.194	1.0592
H	-5.8897	-0.881	-0.0894
H	-5.0911	1.2819	-1.0055
H	-4.2722	-2.5094	0.8639
H	4.1904	-2.4711	-0.9029
H	1.7091	-2.5932	-0.9505
H	-3.303	2.5565	-1.411
H	-0.8418	-2.0116	0.8703
H	-0.8405	2.2849	0.831
H	3.9771	1.5751	0.503
O	5.5924	-0.3332	-0.1542
H	5.9941	-1.1582	-0.4457

Table S4. (cont.)

Compound 93			
N_i = 0	E = -953.706652797		
C	4.4831	0.0484	-0.0914
C	3.6395	-1.031	0.1981
C	2.2696	-0.8529	0.2112
C	1.7184	0.4043	-0.0612
C	2.5638	1.4731	-0.3635
C	3.9393	1.2926	-0.378
C	0.2598	0.5858	-0.0174
C	-0.382	1.7684	0.1629
C	-1.8152	1.8575	0.1906
O	-2.4214	2.9295	0.3372
C	-2.519	0.5745	0.0487
C	-3.9156	0.4875	0.0857
C	-4.5309	-0.741	-0.0421
C	-3.7636	-1.9034	-0.2115
C	-2.3875	-1.8304	-0.2492
C	-1.7722	-0.5883	-0.1161
O	-0.407	-0.5714	-0.162
O	4.154	-2.2659	0.4759
O	5.8189	-0.2144	-0.0755
H	1.639	-1.7	0.4501
H	2.1604	2.4481	-0.6062
H	4.6018	2.1175	-0.6169
H	-4.2608	-2.8624	-0.3121
H	-1.7822	-2.7192	-0.3774
H	5.1163	-2.2296	0.4235
H	6.3238	0.5887	-0.2419
H	0.1903	2.6745	0.3063
H	-4.5057	1.3864	0.2173
O	-5.8962	-0.7874	0.0055
H	-6.1956	-1.6971	-0.092

Compound 94			
N_i = 0	E = -953.708043980		
C	-4.5301	-0.4365	-0.0239
C	-3.6116	-1.4351	0.2818
C	-2.2463	-1.1748	0.2969
C	-1.8007	0.1143	0.0088
C	-2.7039	1.1295	-0.3036
C	-4.0634	0.8413	-0.3167
C	-0.354	0.4055	0.0479
C	0.1898	1.6204	0.2997
C	1.6146	1.8203	0.3264
O	2.1353	2.9234	0.5418
C	2.4129	0.609	0.091
C	3.8122	0.6323	0.1053
C	4.5216	-0.5302	-0.1167
C	3.846	-1.7367	-0.3576
C	2.4681	-1.7724	-0.3736
C	1.758	-0.596	-0.147
O	0.3951	-0.6854	-0.1772
O	-4.0082	-2.7065	0.5813
O	-4.9153	1.8598	-0.6339
H	-5.5942	-0.6516	-0.0393
H	-1.5542	-1.9685	0.5451
H	-2.3721	2.1287	-0.5554
H	4.4155	-2.643	-0.5331
H	1.9334	-2.6958	-0.5587
H	-4.9685	-2.7675	0.5534
H	-5.8244	1.5431	-0.6289
H	-0.4541	2.465	0.5034
H	4.3311	1.5647	0.2923
O	5.8863	-0.4645	-0.092
H	6.2628	-1.3362	-0.2508

Table S4. (cont.)

Compound 95			
N_i = 0	E = -953.702073130		
C	4.9865	-0.5827	0.1612
C	4.0303	-1.457	-0.3239
C	2.6863	-1.0873	-0.3684
C	2.2978	0.1942	0.0684
C	3.2862	1.0558	0.5669
C	4.6162	0.6818	0.6162
C	0.9082	0.6723	0.0024
C	0.5229	1.9572	-0.167
C	-0.8698	2.3371	-0.1965
O	-1.2321	3.5143	-0.329
C	-1.8226	1.2309	-0.0769
C	-3.21	1.4145	-0.1129
C	-4.0591	0.3348	-0.0049
C	-3.5411	-0.9564	0.1396
C	-2.1674	-1.1699	0.1773
C	-1.3275	-0.0642	0.0695
O	0.0069	-0.3287	0.1292
H	2.9931	2.0314	0.9363
H	5.3574	1.3646	1.0128
H	1.268	2.7297	-0.2986
H	-3.6082	2.4148	-0.2272
H	4.3032	-2.4453	-0.6755
O	-1.613	-2.4021	0.3162
H	-2.3119	-3.0636	0.3804
O	1.8322	-2.0167	-0.8792
H	0.9156	-1.7526	-0.7312
H	6.0249	-0.8923	0.1928
H	-5.1352	0.4664	-0.0306
O	-4.3163	-2.0654	0.2506
H	-5.2495	-1.828	0.2326

Compound 96			
N_i = 0	E = -953.704569509		
C	-4.8205	-0.2071	0.1297
C	-3.8619	-1.1604	-0.2026
C	-2.5137	-0.8251	-0.2338
C	-2.1177	0.4786	0.0656
C	-3.0739	1.4392	0.4075
C	-4.4157	1.0869	0.4351
C	-0.6868	0.83	0.0149
C	-0.179	2.0754	-0.1235
C	1.2443	2.3195	-0.1608
O	1.7189	3.4592	-0.2668
C	2.0829	1.1234	-0.0766
C	3.4825	1.1624	-0.1234
C	4.2162	-0.0007	-0.0501
C	3.5704	-1.236	0.0709
C	2.1837	-1.304	0.1233
C	1.4584	-0.1166	0.0485
O	0.1084	-0.2555	0.1081
H	-2.7775	2.4477	0.6663
H	-5.1597	1.8274	0.7043
H	-0.8461	2.9211	-0.2204
H	3.98	2.1191	-0.2218
O	-4.1904	-2.4509	-0.5114
O	1.6006	-2.528	0.2429
H	0.6402	-2.4464	0.2134
H	-5.87	-0.4813	0.152
H	-1.7878	-1.5832	-0.5006
H	-5.1449	-2.5663	-0.466
H	5.2988	0.0154	-0.0883
O	4.3246	-2.3632	0.1298
H	3.7548	-3.1393	0.191

Table S4. (cont.)

Compound 97			
N_i = 0	E = -953.706295835		
C	-4.685	-0.5752	-0.009
C	-3.6751	-1.4838	-0.3191
C	-2.3548	-1.0695	-0.3118
C	-2.0192	0.2541	-0.002
C	-3.0447	1.1513	0.3123
C	-4.3678	0.7446	0.309
C	-0.6158	0.6859	-0.0138
C	-0.1687	1.9626	-0.0906
C	1.2368	2.2828	-0.0864
O	1.6541	3.4502	-0.1346
C	2.1385	1.1304	-0.0349
C	3.5345	1.245	-0.0503
C	4.3283	0.12	-0.007
C	3.7478	-1.1514	0.0513
C	2.3658	-1.2941	0.0672
C	1.5793	-0.1448	0.0247
O	0.2379	-0.3576	0.0514
H	-2.8182	2.1763	0.5803
H	-5.1577	1.4439	0.5605
H	-0.876	2.7767	-0.1695
H	3.9827	2.2295	-0.0986
O	1.8463	-2.551	0.1234
H	0.8833	-2.516	0.0894
O	-5.9687	-1.027	-0.026
H	-1.5776	-1.7824	-0.5583
H	-3.9331	-2.507	-0.565
H	-6.5725	-0.3158	0.2137
H	5.4089	0.1951	-0.0193
O	4.5593	-2.2396	0.0877
H	4.0308	-3.0465	0.1127

Compound 98			
N_i = 0	E = -953.706600899		
C	-4.6739	0.0883	0.2994
C	-3.846	-0.9335	-0.1263
C	-2.4596	-0.7437	-0.2111
C	-1.9075	0.4986	0.1304
C	-2.7616	1.5208	0.5719
C	-4.1263	1.319	0.6525
C	-0.4649	0.7681	0.0245
C	0.09	1.9746	-0.2237
C	1.5219	2.1519	-0.2836
O	2.0457	3.2567	-0.4859
C	2.3046	0.9303	-0.1038
C	3.7062	0.9109	-0.1603
C	4.4066	-0.2612	0.0012
C	3.7096	-1.4617	0.2234
C	2.3259	-1.4789	0.2863
C	1.6489	-0.2795	0.1223
O	0.2878	-0.3426	0.2051
O	-1.7683	-1.8243	-0.6717
O	-4.3896	-2.1392	-0.4668
H	-5.7411	-0.0911	0.3612
H	-2.3389	2.4722	0.8695
H	-4.7721	2.1155	1.0012
H	1.7819	-2.3987	0.4603
H	-0.8232	-1.7211	-0.503
H	-3.679	-2.7501	-0.6965
H	-0.5443	2.8338	-0.3925
H	4.2338	1.8409	-0.3361
H	5.49	-0.2735	-0.0422
O	4.3549	-2.6428	0.3862
H	5.3079	-2.5157	0.3237

Table S4. (cont.)

Compound 99			
N_i = 0	E = -953.711448908		
C	4.5645	-0.3219	-0.122
C	3.6705	-1.2843	0.322
C	2.3082	-1.0116	0.3575
C	1.8223	0.2504	-0.0461
C	2.756	1.1953	-0.4951
C	4.1093	0.9317	-0.5387
C	0.4043	0.6179	0.0087
C	-0.0828	1.8677	0.1907
C	-1.4992	2.1354	0.2102
O	-1.9585	3.2787	0.3551
C	-2.3556	0.958	0.0657
C	-3.7565	1.0278	0.0896
C	-4.5257	-0.104	-0.0474
C	-3.9008	-1.3515	-0.2123
C	-2.5202	-1.4586	-0.2364
C	-1.773	-0.298	-0.0976
O	-0.4173	-0.449	-0.1437
O	1.5224	-2.0163	0.8299
O	5.8816	-0.6595	-0.1395
H	4.0263	-2.2564	0.6412
H	2.4028	2.1608	-0.8371
H	4.8092	1.6756	-0.901
O	-4.6199	-2.4917	-0.3569
H	-2.0321	-2.4164	-0.3648
H	0.5904	-1.808	0.6888
H	6.4095	0.073	-0.4747
H	0.5996	2.6925	0.3432
H	-4.2289	1.9943	0.2185
H	-5.6085	-0.0474	-0.0307
H	-5.5618	-2.291	-0.3361

Compound 100			
N_i = 0	E = -953.706461603		
C	4.5515	-0.9046	0.0056
C	3.566	-1.7671	-0.441
C	2.2296	-1.3783	-0.4725
C	1.8798	-0.0835	-0.0534
C	2.8849	0.7767	0.4067
C	4.2077	0.3752	0.435
C	0.4971	0.4196	-0.1012
C	0.1361	1.6976	-0.3479
C	-1.2501	2.1054	-0.3536
O	-1.5952	3.2769	-0.5629
C	-2.2114	1.0313	-0.1095
C	-3.5989	1.2407	-0.0967
C	-4.4708	0.2018	0.1287
C	-3.9675	-1.0922	0.3484
C	-2.6041	-1.3356	0.34
C	-1.7506	-0.2663	0.1126
O	-0.416	-0.5525	0.1288
O	1.3438	-2.2994	-0.9621
O	5.1373	1.2706	0.9025
H	5.5874	-1.226	0.0297
H	3.8233	-2.7653	-0.7755
H	2.6276	1.7673	0.7621
O	-4.7878	-2.1469	0.5785
H	-2.2085	-2.3291	0.5089
H	0.4379	-2.0234	-0.7799
H	6.0142	0.8752	0.8738
H	0.8946	2.4393	-0.5576
H	-3.9772	2.2416	-0.2673
H	-5.5425	0.3664	0.1411
H	-5.7079	-1.8612	0.5778

Table S4. (cont.)

Compound 101			
N_i = 0	E = -953.705974250		
C	-4.6914	-0.9381	0.0798
C	-4.3487	0.2712	0.6676
C	-3.0288	0.7044	0.6182
C	-2.035	-0.0661	-0.0122
C	-2.4126	-1.3004	-0.5757
C	-3.7352	-1.7275	-0.5393
C	-0.6463	0.4143	-0.0967
C	-0.2539	1.6525	-0.4583
C	1.1433	2.0167	-0.5232
O	1.5169	3.1539	-0.8435
C	2.0776	0.9403	-0.2005
C	3.4704	1.109	-0.234
C	4.3184	0.0677	0.0598
C	3.7842	-1.1872	0.4008
C	2.4148	-1.3896	0.4433
C	1.5849	-0.3192	0.1423
O	0.2436	-0.5647	0.2073
O	-2.6555	1.8767	1.1952
O	-1.532	-2.1189	-1.2187
H	-5.7214	-1.274	0.1137
H	-5.0917	0.8829	1.1674
H	-3.993	-2.6759	-0.9941
O	4.5807	-2.2428	0.6998
H	1.997	-2.3533	0.7053
H	-3.424	2.2918	1.6006
H	-0.6217	-1.8778	-1.0105
H	-0.9992	2.3931	-0.7113
H	3.8718	2.0798	-0.4992
H	5.3945	0.1997	0.0331
H	5.5079	-1.9874	0.6433

Compound 102			
N_i = 0	E = -953.709944028		
C	-4.3935	-0.0827	0.1256
C	-3.4949	-1.0931	-0.2386
C	-2.1385	-0.8331	-0.2696
C	-1.657	0.44	0.055
C	-2.5562	1.4415	0.4234
C	-3.9183	1.1783	0.4581
C	-0.2132	0.7107	-0.0002
C	0.3547	1.929	-0.163
C	1.7849	2.0988	-0.1969
O	2.3226	3.2102	-0.3269
C	2.5553	0.862	-0.0796
C	3.9576	0.8344	-0.1147
C	4.6472	-0.3499	-0.0036
C	3.9377	-1.5542	0.1454
C	2.5536	-1.5637	0.1834
C	1.8841	-0.3525	0.0686
O	0.5239	-0.4135	0.1178
O	-3.942	-2.3413	-0.5687
O	-5.7098	-0.4302	0.1313
H	-1.4638	-1.6287	-0.56
H	-2.2027	2.4258	0.7042
H	-4.6225	1.9491	0.7515
O	4.5735	-2.7478	0.2567
H	2.0018	-2.4881	0.2978
H	-4.9038	-2.3628	-0.5035
H	-6.2585	0.3136	0.4014
H	-0.2721	2.8018	-0.2852
H	4.4947	1.7682	-0.2318
H	5.7311	-0.3685	-0.031
H	5.5279	-2.6233	0.2145

Table S4. (cont.)

Compound 103			
N_i = 0	E = -953.711369518		
C	-4.4151	-0.6301	0.0306
C	-4.0433	0.6712	0.3533
C	-2.7103	1.0634	0.33
C	-1.7364	0.1292	-0.0212
C	-2.0867	-1.1828	-0.3377
C	-3.4277	-1.5484	-0.3105
C	-0.317	0.5323	-0.0662
C	0.1345	1.7896	-0.2702
C	1.5463	2.0901	-0.3009
O	1.9777	3.2396	-0.4755
C	2.4244	0.936	-0.1206
C	3.8243	1.0377	-0.1302
C	4.6173	-0.0708	0.0476
C	4.0182	-1.3278	0.2401
C	2.641	-1.4659	0.253
C	1.8652	-0.3283	0.0725
O	0.5161	-0.514	0.1047
O	-4.9659	1.6108	0.7132
O	-3.7287	-2.8403	-0.6333
H	-5.4589	-0.9275	0.0526
H	-2.4521	2.0775	0.6066
H	-1.3398	-1.9148	-0.6146
O	4.7619	-2.4467	0.4234
H	2.1752	-2.4316	0.4032
H	-5.8469	1.2226	0.7203
H	-4.6795	-2.9832	-0.5823
H	-0.5694	2.5952	-0.4287
H	4.2757	2.0113	-0.2793
H	5.6987	0.0099	0.043
H	5.6994	-2.2262	0.4013

Compound 104			
N_i = 0	E = -953.699972499		
C	4.4464	0.3827	-0.4839
C	3.753	-0.5923	0.2113
C	2.3661	-0.4969	0.3818
C	1.6815	0.5931	-0.1603
C	2.3932	1.5774	-0.8573
C	3.7634	1.4727	-1.0178
C	0.2239	0.7358	-0.0072
C	-0.4042	1.8894	0.3145
C	-1.8421	1.9761	0.3889
O	-2.4359	3.0282	0.656
C	-2.5553	0.7152	0.1362
C	-3.9514	0.6099	0.1945
C	-4.5552	-0.6074	-0.0452
C	-3.7909	-1.7411	-0.3419
C	-2.4141	-1.658	-0.4024
C	-1.8063	-0.4174	-0.1656
O	-0.4501	-0.4018	-0.2571
O	1.702	-1.4424	1.0981
O	4.3398	-1.688	0.78
H	5.5203	0.2849	-0.6034
H	1.8537	2.4137	-1.285
H	4.3071	2.2328	-1.5651
O	-1.6779	-2.7646	-0.6896
H	2.3403	-2.0752	1.4495
H	5.2951	-1.6541	0.6653
H	0.1846	2.7727	0.5232
H	-4.5373	1.4896	0.4297
H	-5.634	-0.6964	-0.0026
H	-4.2653	-2.6981	-0.5256
H	-0.7365	-2.5555	-0.6586

Table S4. (cont.)

Compound 105			
N_i = 0	E = -953.705730650		
C	-4.4125	-0.0625	-0.119
C	-3.5897	-1.052	0.3975
C	-2.2133	-0.8651	0.4382
C	-1.6413	0.333	-0.036
C	-2.5044	1.3078	-0.5566
C	-3.8713	1.1297	-0.6059
C	-0.2041	0.6157	0.0193
C	0.3457	1.8391	0.206
C	1.7706	2.041	0.2213
O	2.2864	3.1557	0.3738
C	2.5803	0.8226	0.0597
C	3.9798	0.8448	0.0744
C	4.6836	-0.3321	-0.0828
C	4.0138	-1.5453	-0.2616
C	2.6323	-1.5892	-0.2804
C	1.924	-0.3918	-0.1105
O	0.5628	-0.4883	-0.14
O	-1.5009	-1.8834	0.9899
O	-5.7493	-0.309	-0.134
H	-4.0128	-1.9747	0.7754
H	-2.0822	2.2249	-0.95
H	-4.5174	1.8936	-1.0228
H	-0.555	-1.722	0.9042
H	-6.2194	0.4428	-0.51
H	-0.2976	2.6935	0.3662
H	4.4897	1.7904	0.2096
H	5.7669	-0.3239	-0.0728
H	4.5642	-2.4697	-0.3928
O	2.0071	-2.7831	-0.4603
H	1.0533	-2.6649	-0.5234

Compound 106			
N_i = 0	E = -953.700772588		
C	-4.4243	-0.6622	0.0322
C	-3.4879	-1.5583	0.5165
C	-2.1342	-1.2356	0.5488
C	-1.7167	0.0224	0.0852
C	-2.6729	0.9202	-0.4062
C	-4.0143	0.5858	-0.4316
C	-0.3097	0.4528	0.1151
C	0.1167	1.7107	0.3643
C	1.5175	2.0558	0.3523
O	1.9224	3.2037	0.5675
C	2.4362	0.9389	0.081
C	3.8269	1.0985	0.0514
C	4.6348	0.0077	-0.1984
C	4.0807	-1.2554	-0.425
C	2.7106	-1.4338	-0.403
C	1.8967	-0.3229	-0.1447
O	0.5527	-0.5565	-0.1395
O	-1.3016	-2.181	1.0823
O	-4.8963	1.5119	-0.9297
H	-5.475	-0.9313	0.0102
H	-3.799	-2.5292	0.8839
H	-2.3612	1.8851	-0.7881
H	-0.3794	-1.929	0.9664
H	-5.7919	1.1614	-0.8923
H	-0.6049	2.4834	0.5918
H	4.2483	2.0804	0.2266
H	5.7116	0.1232	-0.2222
H	4.7136	-2.1125	-0.6237
O	2.1914	-2.6703	-0.626
H	1.228	-2.6416	-0.6174

Table S4. (cont.)

Compound 107			
N_i = 0	E = -953.701177418		
C	-4.5716	-0.5536	-0.0581
C	-4.0742	0.4234	-0.9085
C	-2.7227	0.7528	-0.8507
C	-1.8697	0.1071	0.0518
C	-2.3994	-0.876	0.8977
C	-3.7487	-1.2093	0.8478
C	-0.4382	0.4531	0.1303
C	0.051	1.6494	0.5148
C	1.4709	1.9067	0.5721
O	1.938	2.9982	0.9177
C	2.3241	0.7684	0.2012
C	3.7238	0.8367	0.2189
C	4.4663	-0.2704	-0.1362
C	3.8399	-1.4626	-0.5158
C	2.4622	-1.5503	-0.5388
C	1.712	-0.423	-0.1758
O	0.3609	-0.5746	-0.224
O	-2.1776	1.6929	-1.6685
O	-1.5377	-1.4622	1.7726
H	-5.624	-0.8095	-0.1018
H	-4.7186	0.9294	-1.6187
H	-4.1396	-1.9688	1.5156
H	-2.8584	2.059	-2.243
H	-2.0092	-2.0885	2.3319
H	-0.6381	2.436	0.7918
H	4.2028	1.7618	0.5141
H	5.5487	-0.2252	-0.1246
H	4.4234	-2.332	-0.7957
O	1.8611	-2.7122	-0.9093
H	0.902	-2.6128	-0.882

Compound 108			
N_i = 0	E = -953.704594122		
C	-4.2221	0.1743	-0.0864
C	-3.3769	-0.8997	0.2187
C	-2.0077	-0.7181	0.2341
C	-1.4594	0.5377	-0.0517
C	-2.3059	1.6011	-0.3692
C	-3.6807	1.4166	-0.3861
C	-0.0027	0.7215	-0.0074
C	0.6498	1.9015	0.1344
C	2.0869	1.9801	0.1619
O	2.6962	3.0527	0.2754
C	2.7876	0.6906	0.0609
C	4.1837	0.5781	0.0939
C	4.7706	-0.6677	0.0018
C	3.9897	-1.8219	-0.123
C	2.6121	-1.7307	-0.1563
C	2.0222	-0.4631	-0.0629
O	0.6637	-0.4442	-0.1092
O	-3.889	-2.1324	0.5092
O	-5.5568	-0.0919	-0.0717
H	-1.3752	-1.5612	0.4837
H	-1.9038	2.5743	-0.6215
H	-4.3447	2.2368	-0.6366
H	-4.8513	-2.099	0.455
H	-6.0638	0.7072	-0.251
H	0.086	2.8177	0.2431
H	4.7827	1.4749	0.1915
H	5.8495	-0.7629	0.0263
H	4.4509	-2.7999	-0.195
O	1.8567	-2.8549	-0.2767
H	0.9202	-2.6234	-0.2888

Table S4. (cont.)

Compound 109			
N_i = 0	E = -953.705967192		
C	4.2722	0.3898	-0.0054
C	3.8429	-0.9019	0.2842
C	2.4921	-1.2279	0.2792
C	1.5589	-0.2371	-0.0235
C	1.9671	1.065	-0.31
C	3.3245	1.3638	-0.3009
C	0.1206	-0.5644	-0.0506
C	-0.4088	-1.7955	-0.2361
C	-1.834	-2.0166	-0.2491
O	-2.3338	-3.1384	-0.3987
C	-2.6559	-0.8085	-0.0845
C	-4.0567	-0.8357	-0.096
C	-4.7636	0.339	0.0609
C	-4.0997	1.5592	0.2285
C	-2.7196	1.6066	0.2405
C	-2.0073	0.4096	0.0844
O	-0.6531	0.5238	0.1165
O	4.7255	-1.897	0.5899
O	3.6813	2.6479	-0.595
H	5.3299	0.6346	0.0038
H	2.1913	-2.2374	0.5285
H	1.2526	1.8415	-0.5493
H	5.6247	-1.5529	0.5823
H	4.6391	2.7398	-0.5689
H	0.2437	-2.6435	-0.3933
H	-4.5645	-1.7827	-0.2293
H	-5.8469	0.3261	0.0542
H	-4.6553	2.4817	0.3499
O	-2.0805	2.7955	0.4028
H	-1.1253	2.6659	0.367

Compound 110			
N_i = 0	E = -953.703205638		
C	4.0566	0.3904	-0.183
C	3.3247	-0.7397	0.1626
C	1.9323	-0.6948	0.2093
C	1.2562	0.5046	-0.0785
C	2.0199	1.6265	-0.4314
C	3.3982	1.5792	-0.4843
C	-0.204	0.6175	-0.0067
C	-0.8976	1.7515	0.2562
C	-2.3366	1.7723	0.2766
O	-2.9877	2.8039	0.4914
C	-2.9833	0.4724	0.0471
C	-4.3754	0.3073	0.0713
C	-4.9346	-0.9375	-0.1305
C	-4.1108	-2.0474	-0.3605
C	-2.7366	-1.9116	-0.3912
C	-2.191	-0.6478	-0.1881
O	-0.8288	-0.5586	-0.2388
O	1.3499	-1.8673	0.5816
O	4.015	-1.8829	0.4506
H	1.5172	2.5501	-0.6893
H	3.977	2.4489	-0.7692
H	0.4018	-1.8451	0.3979
H	3.3908	-2.6023	0.6038
H	-0.3675	2.6688	0.4721
H	-4.9994	1.1741	0.2542
H	-6.0107	-1.0603	-0.1102
H	-4.5525	-3.0247	-0.5163
H	-2.0796	-2.7545	-0.568
O	5.4138	0.346	-0.2433
H	5.7188	-0.5416	-0.0202

Table S4. (cont.)

Compound 111			
N_i = 0	E = -953.702145144		
C	4.1386	-0.0979	0.098
C	3.3147	-1.1623	-0.2183
C	1.9231	-1.013	-0.2622
C	1.3641	0.2389	0.014
C	2.2008	1.3126	0.3479
C	3.5724	1.1414	0.3847
C	-0.0882	0.4712	-0.053
C	-0.6764	1.6384	-0.3989
C	-2.1108	1.7848	-0.4197
O	-2.6684	2.8498	-0.7122
C	-2.8644	0.5698	-0.0821
C	-4.2661	0.5306	-0.0807
C	-4.9311	-0.636	0.2337
C	-4.2065	-1.7921	0.553
C	-2.8254	-1.7804	0.5593
C	-2.171	-0.5941	0.2408
O	-0.8054	-0.6257	0.2704
O	1.2366	-2.1383	-0.6311
O	3.88	-2.3717	-0.4934
H	1.7789	2.2776	0.5981
H	0.309	-2.0652	-0.3757
H	3.1789	-3.0154	-0.6538
H	-0.0654	2.4857	-0.6781
H	-4.8124	1.4319	-0.332
H	-6.0141	-0.6614	0.2336
H	-4.732	-2.7074	0.7991
H	-2.245	-2.6616	0.8041
H	5.2132	-0.2416	0.1307
O	4.3415	2.2247	0.7219
H	5.2712	1.976	0.7311

Compound 112			
N_i = 0	E = -953.698597308		
C	-4.1681	-0.9213	0.121
C	-3.7907	0.3299	-0.328
C	-2.4432	0.6934	-0.3316
C	-1.4634	-0.2002	0.1228
C	-1.8687	-1.4777	0.56
C	-3.2103	-1.8258	0.5615
C	-0.0489	0.2057	0.1547
C	0.4278	1.3829	0.6116
C	1.8406	1.6756	0.6183
O	2.2955	2.7515	1.0258
C	2.701	0.5939	0.1218
C	4.0986	0.7046	0.0787
C	4.8679	-0.3419	-0.3846
C	4.2544	-1.5267	-0.8135
C	2.8806	-1.662	-0.7807
C	2.1189	-0.5948	-0.3127
O	0.7642	-0.7718	-0.3089
O	-2.0801	1.9155	-0.7975
O	-4.6633	1.2802	-0.796
H	-2.8716	2.3756	-1.1037
H	-5.562	0.9358	-0.7927
H	-0.2615	2.124	0.9911
H	4.5587	1.6255	0.4168
H	5.9469	-0.251	-0.4166
H	4.8613	-2.3482	-1.1758
H	2.3856	-2.5683	-1.1074
H	-5.218	-1.1939	0.1183
H	-3.4994	-2.8092	0.9108
O	-0.995	-2.4127	1.0479
H	-0.0894	-2.206	0.7912

Table S4. (cont.)

Compound 113			
N_i = 0	E = -953.703666852		
C	4.0217	-0.4359	0.032
C	3.1431	-1.4635	0.3189
C	1.767	-1.248	0.3071
C	1.2601	0.0297	0.0129
C	2.1746	1.0545	-0.2901
C	3.5315	0.8399	-0.2847
C	-0.1697	0.3523	0.0427
C	-0.6978	1.5753	0.2948
C	-2.118	1.8036	0.2922
O	-2.6178	2.9176	0.5026
C	-2.9417	0.6119	0.0429
C	-4.343	0.652	0.0391
C	-5.0738	-0.4964	-0.185
C	-4.4159	-1.7129	-0.4098
C	-3.0362	-1.7793	-0.4119
C	-2.316	-0.6102	-0.1853
O	-0.9543	-0.7197	-0.2068
O	1.0044	-2.3325	0.6299
H	0.0745	-2.1512	0.4464
H	-0.0447	2.406	0.5237
H	-4.8368	1.5999	0.2174
H	-6.1565	-0.4604	-0.1869
H	-4.992	-2.6139	-0.5852
H	-2.5068	-2.7085	-0.5846
H	3.5221	-2.4513	0.5559
H	1.8138	2.0412	-0.5542
O	5.3719	-0.5794	0.021
H	5.6235	-1.4827	0.242
O	4.3828	1.8688	-0.5953
H	5.2905	1.5451	-0.5819

Compound 114			
N_i = 0	E = -953.705948370		
C	4.1831	-0.3495	-0.0244
C	3.3341	-1.3437	0.4439
C	1.9648	-1.1182	0.4522
C	1.416	0.1072	0.0172
C	2.3153	1.0824	-0.4615
C	3.6832	0.8633	-0.4849
C	-0.026	0.3667	0.0794
C	-0.6198	1.518	0.4688
C	-2.0526	1.6615	0.501
O	-2.6103	2.7119	0.8469
C	-2.809	0.4632	0.1139
C	-4.2108	0.4262	0.1097
C	-4.8773	-0.7273	-0.2471
C	-4.1539	-1.8724	-0.6063
C	-2.7728	-1.8627	-0.609
C	-2.1163	-0.6892	-0.2484
O	-0.7509	-0.7221	-0.2795
O	1.2064	-2.1348	0.9441
H	0.2759	-2.0029	0.7244
H	-0.0076	2.3546	0.7727
H	-4.7561	1.3188	0.3926
H	-5.9604	-0.751	-0.2498
H	-4.6803	-2.7775	-0.8861
H	-2.1941	-2.7361	-0.8838
H	3.7225	-2.2922	0.7951
O	1.8007	2.2475	-0.9274
O	5.5311	-0.5165	-0.0649
H	5.7693	-1.3882	0.2685
H	4.3536	1.6246	-0.8666
H	2.5146	2.809	-1.2479

Table S4. (cont.)

Compound 115			
$N_i = 0$	E = -953.704588977		
C	3.8605	-0.2279	-0.0118
C	2.9917	-1.283	0.2627
C	1.6213	-1.081	0.2787
C	1.1102	0.1939	0.0232
C	1.9733	1.2535	-0.2587
C	3.3421	1.0385	-0.2755
C	-0.344	0.4138	0.0567
C	-0.9557	1.6013	0.2855
C	-2.389	1.7253	0.2886
O	-2.9693	2.8039	0.4789
C	-3.1195	0.4713	0.0629
C	-4.5205	0.4083	0.0534
C	-5.1643	-0.793	-0.1561
C	-4.4172	-1.9613	-0.3598
C	-3.0369	-1.9263	-0.3557
C	-2.4014	-0.7048	-0.1439
O	-1.0378	-0.7199	-0.1578
H	-0.3619	2.4823	0.4872
H	-5.0834	1.3202	0.2143
H	-6.2467	-0.8368	-0.1632
H	-4.925	-2.9047	-0.5239
H	-2.4417	-2.8174	-0.513
O	5.2125	-0.3836	-0.042
H	5.4362	-1.3057	0.1312
H	0.9666	-1.9142	0.5028
H	1.6022	2.2442	-0.4893
O	3.5872	-2.4868	0.5068
H	2.922	-3.1669	0.6551
O	4.1749	2.081	-0.5643
H	5.0882	1.7712	-0.5757
